# RASSF10 Is a TGFβ-Target That Regulates ASPP2 and E-Cadherin Expression and Acts as Tumor Suppressor That Is Epigenetically Downregulated in Advanced Cancer

**DOI:** 10.3390/cancers11121976

**Published:** 2019-12-08

**Authors:** Antje M. Richter, Miriam M. Küster, Michelle L. Woods, Sara K. Walesch, Mira Y. Gökyildirim, Marcus Krueger, Reinhard H. Dammann

**Affiliations:** 1 Institute for Genetics, Justus-Liebig-University Giessen, 35452 Giessen, Germany; Miriam.Kuester@gen.bio.uni-giessen.de (M.M.K.); michelle.woods@bio.uni-giessen.de (M.L.W.); sara.walesch@gen.bio.uni-giessen.de (S.K.W.); mira.y.goekyildirim@innere.med.uni-giessen.de (M.Y.G.); 2 Institute for Genetics, University of Cologne, 50931 Köln, Germany; marcus.krueger@uni-koeln.de; 3 German Center for Lung Research (DZL), Universities of Giessen and Marburg Lung Center, 35452 Giessen, Germany

**Keywords:** RASSF, ASPP, TGFβ, EMT, E-cadherin, epigenetic, tumor suppressor

## Abstract

The *Ras Association Domain Family* (RASSF) encodes members of tumor suppressor genes which are frequently inactivated in human cancers. Here, the function and the regulation of RASSF10, that contains a RA (Ras-association) and two coiled domains, was investigated. We utilized mass spectrometry and immuno-precipitation to identify interaction partners of RASSF10. Additionally, we analyzed the up- and downstream pathways of RASSF10 that are involved in its tumor suppressive function. We report that RASSF10 binds ASPP1 (Apoptosis-stimulating protein of p53) and ASPP2 through its coiled-coils. Induction of RASSF10 leads to increased protein levels of ASPP2 and acts negatively on cell cycle progression. Interestingly, we found that RASSF10 is a target of the EMT (epithelial mesenchymal transition) driver TGFβ (Transforming growth factor beta) and that negatively associated genes of RASSF10 are significantly over-represented in an EMT gene set collection. We observed a positive correlation of RASSF10 expression and E-cadherin that prevents EMT. Depletion of RASSF10 by CRISPR/Cas9 technology induces the ability of lung cancer cells to proliferate and to invade an extracellular matrix after TGFβ treatment. Additionally, knockdown of RASSF10 or ASPP2 induced constitutive phosphorylation of SMAD2 (Smad family member 2). Moreover, we found that epigenetic reduction of RASSF10 levels correlates with tumor progression and poor survival in human cancers. Our study indicates that RASSF10 acts a TGFβ target gene and negatively regulates cell growth and invasion through ASPP2. This data suggests that epigenetic loss of RASSF10 contributes to tumorigenesis by promoting EMT induced by TGFβ.

## 1. Introduction

Cancer is caused by a multistep genetic and epigenetic transformation of normal cells into highly invasive and immortal tumor cells. Epithelial cells are immobile cells that are responsible for organ integrity and structure. A key event of the transformation of epithelial cells into invasive tumor cells is associated with increased motility and disruption of cell-adhesion referred to as epithelial to mesenchymal transition (EMT). EMT disrupts cell polarity and contact inhibition of epithelial cells transforming them in a mesenchymal phenotype with increased invasion and cell motility. Different genetic and epigenetic alterations have been identified that are associated with EMT [1]. *Epithelial cadherin* (CDH1) is a master mediator of cell–cell adherens junctions and loss of CDH1 expression is associated with disruption of apical-basal polarity and integrity of epithelial cells [2]. Aberrant signaling by transforming growth factor beta (TGFβ) and RAS (rat sarcoma) induces EMT by activating the expression of SNAI1 (snail family transcriptional repressor 1) that acts as a repressor of CDH1 transcription [3,4].

The *Ras Association Domain Family* (RASSF) consists of ten members and several of them are epigenetically silenced in different tumor entities [5]. The RASSFs differ substantially in their tumor-suppressor pathways [5,6,7]. All RASSFs harbor an eponymous RAS-association domain (RA). However, the presence of the RA domain does not necessarily imply RAS binding for all members [8]. For the first six members the RA domain is located upstream of the C-terminal SARAH (Sav-RASSF-Hippo) domain that encodes an interaction module connecting the members to the Hippo pathway through the Hippo kinases MST1 (Mammalian sterile 20-like 1) or MST2 [9,10,11,12]. For example, it has been shown that RASSF1A regulates organ size through inhibition of the protooncogene YAP (Yes-associated protein) [13,14,15]. Thus, RASSF1A is an important tumor suppressor gene that is frequently hypermethylated in human cancers [5,16].

RASSF10 encodes an N-terminal RA domain and harbors central coiled domains (Figure S1) and lacks catalytically active domains [5,6]. *RASSF10* is located at Chr. 11p15.3, contains a large CpG island promoter >2 kb (Figure S1). Epigenetic inactivation of RASSF10 through promoter hypermethylation has been reported in various tumor entities including lung cancer, thyroid cancer, melanoma and several others [17,18,19,20,21,22]. Functional studies have shown that RASSF10 signaling is linked to the cAMP-PKA (Protein kinase A) pathway [19], MMP2 (Matrix metallopeptidase 2) [23], p53 [24] or JNK (c-Jun N-terminale kinase) pathway [25]. 

In our present study, we observed that RASSF10 is activated by TGFβ and prevents EMT through induction of CDH1. Mass spectrometry and protein analysis revealed that RASSF10 interacts and stabilizes the *Apoptosis-Stimulating Protein of p53 2* (ASPP2) which is encoded by the TP53BP2 gene. ASPP2 is a tumor suppressor gene that controls epithelial plasticity and inhibits EMT [26,27]. Furthermore, we found that RASSF10, but not ASPP2, is frequently hypermethylated in human cancers and the loss of RASSF10 is associated with advanced tumor stages and impaired survival of cancer patients. 

## 2. Results

### 2.1. RASSF10 Inhibts Cell Proliferation and Plays a Role TGFβ Induced Signal Transmission 

**Figure 1:**
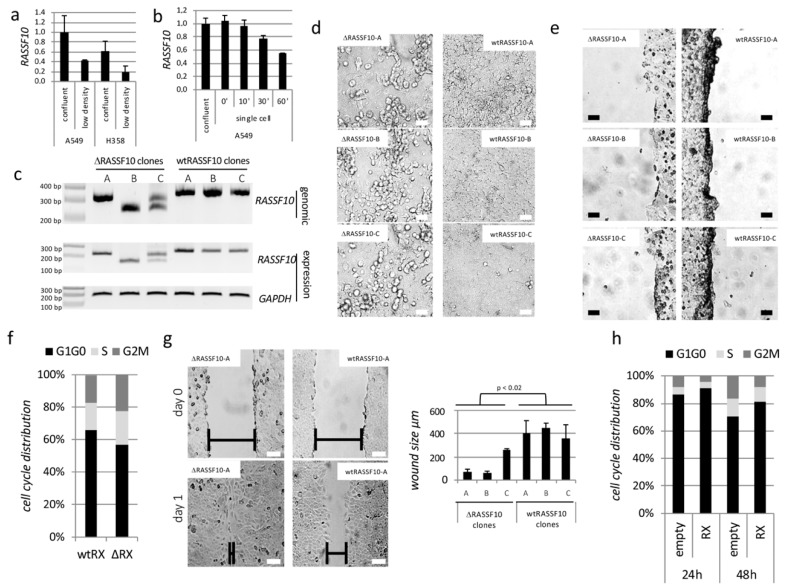
*RASSF10* (Ras Association Domain Family 10) loss increases cell growth. (**a**) Expression of *RASSF10* was measured in confluent (high density) and cells at low density (A549, H358). (**b**) *RASSF10* expression was determined in confluent cells and in single cell suspension in A549. Therefore, confluent cells were trypsinized, resuspended in full medium, placed at 37 °C under constant slow rotation and RNA was isolated at indicated time points. (**c**) Verification of CRISPR/Cas9 RASSF10 knockout in A549 on genomic and expression level by PCR for three RASSF10 negative clones (∆RASSF10) and three wtRASSF10 clones. (expected RASSF10 sizes: genomic wtRASSF10 333 bp; RT wtRASSF10 244 bp, CRISPR/Cas9 induces deletion and shortening of PCR product). (**d**) Morphological differences observed between confluent ∆RASSF10 and wtRASSF10 clones. 100 µm standard white. (**e**) Morphological differences observed between confluent ∆RASSF10 and wtRASSF10 clones when wide scratch was placed in cell culture dish. 100 µm standard black. (**f**) Growth differences by flow cytometry of wtRASSF10 (wtRX) and ∆RASSF10 (∆RX) clones isolated during proliferation at 10% density and (**g**) by wound healing assay. 100 µm standard black or white. (**h**) RASSF10 (RX) re-expression effects on cell cycle progression negatively (HeLa negative for endogenous RASSF10).

We studied human cancer cell lines (CCLE, cancer cell line encyclopedia, Broad Institute, *n* = 917, [28]) and found that expression of *RASSF10* (238755_at) significantly correlated with the expression of genes associated with the GO (gene ontology) terms cell periphery, plasma membrane (apical), epidermal/epithelial cell differentiation and cell–cell junction (Table 1). We further found that ‘*RASSF10* negatively associated’ genes are over-represented in the gene set collection hallmark_EMT (*p* = 4.7 × 10^−7^), whereas ‘*RASSF10* positively associated’ genes are under-represented in the hallmark_EMT (Table S1). We observed that *RASSF10* expression was highest in cell lines reaching confluency (Figure 1a,b).

#### 2.1.1. RASSF10 Loss Induces Cell Growth

These observations indicate that *RASSF10* expression is regulated by cellular contact, density and/or epithelial-mesenchymal transition. Therefore, we generated a RASSF10 knockout in the prototypic epithelial cell line A549 by CRISPR/Cas9 (Figure 1c; frameshift deletion mutation). Loss of RASSF10 was associated with detachment from monolayer (Figure 1d). We observed that at the scratch site the monolayer RASSF10 wildtype (wt) cells adhered strongly to neighboring cells in contrast to RASSF10 knockout cell clones (Figure 1e). RASSF10 loss was also associated with induced proliferation (phase G1/G0 decrease) by flow cytometry and increased wound healing ability (Figure 1f,g). In contrast, RASSF10 re-expression in HeLa cells with epigenetically inactivated endogenous *RASSF10* halted cell proliferation (G1/G0 induction) measured by flow cytometry (Figure 1h).

#### 2.1.2. RASSF10 Is Induced by the EMT Driver TGFβ and RASSF10 Depletion Promotes TGFβ Induced Invasion

**Figure 2:**
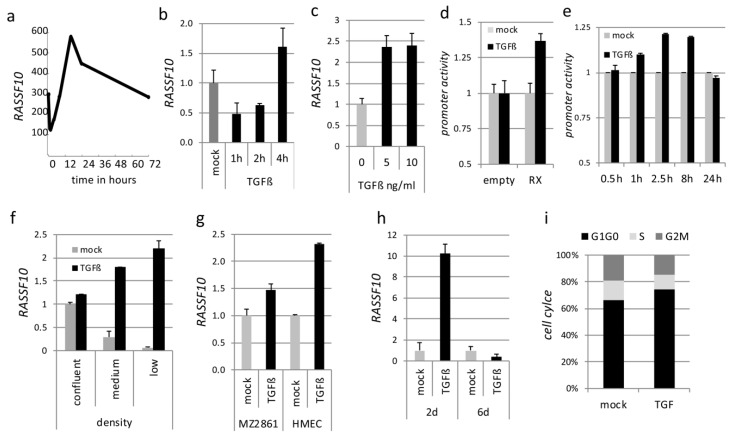
RASSF10 (Ras Association Domain Family 10) is induced by TGFβ treatment. (**a**) RASSF10 expression was found to be dynamically regulated by TGFβ treatment [28] and (**b**) verified by RT-PCR. In general RNA was isolated, DNAse digested, reversely transcribed and expression levels were determined by qPCR in triplicate and normalized to GAPDH. Expression of mock treatment was Set 1 for comparison. (**c**) RASSF10 expression in mock (citrate buffer pH3) or TGFβ treated A549 cells (5 ng/mL; 10 ng/mL; 48 h). (**d**,**e**) RASSF10 promoter activity upon TGFβ treatment (10 ng/mL, 12 h) in HEK (**d**) and in A549 (**e**) (10 ng/ml; varying time points) after transfection of plasmids: pRLnull-empty or pRLnull-RASSF10 promoter (both Renilla Luciferase reporter) together with transfection control pGL3 (Luciferase reporter). Promoter activity was measured using Dual-Glo Luciferase Assay (Promega, Walldorf, Germany) in OrionL Microplate Luminometer (Berthold Detection Systems, Pforzheim, Germany). (**f**) RASSF10 expression induction by TGFβ (10 ng/mL; 60 h) relative to cellular density. (**g**) RASSF10 expression in further cells: kidney cancer MZ2861 (TGFβ 10 ng/mL; 48 h) and breast epithelial cells HMEC (TGFβ 10 ng/mL; 24 h; GEO GDS4071). (**h**) RASSF10 expression regulation varies depending on TGFβ treatment duration (short term 48 h vs. long term 6d; 10 ng/mL). (**i**) TGFβ treatment (72 h; 10 ng/mL) induces cell cycle arrest at phase G1–G0.

As epithelial-mesenchymal transition was found in RASSF10 associated genes, we questioned if RASSF10 was regulated by the EMT driver TGFβ, which is also a modulator of tumorigenesis [29]. We found, that *RASSF10* expression and promoter activity is dynamically regulated by TGFβ (Figure 2a–e) and TGFβ induction of *RASSF10* was further verified in different cell lines, that express *RASSF10* (Figure 2g). Additionally, we observed that induction of *RASSF10* by TGFβ vs. mock was highest under low cellular density (Figure 2f), which was accompanied by the phenotypical spindle like morphology and upregulation of *SNAI2* [30]. We also observed that TGFβ treatment of A549 cells for two days led to an upregulation of *RASSF10* and long-term TGFβ exposure (six days), when cells adopted an EMT program [31], caused an inhibition of *RASSF10* expression (Figure 2h). Consistently, short-term TGFβ treatment led to cell cycle arrest at phase G1–G0 as measured by flow cytometry (Figure 2i) in accordance with earlier results [32]. We assumed that loss of the tumor-suppressor RASSF10 in cancer contributes to the transition of epithelial to mesenchymal cell phenotypes.

To analyze the effect of RASSF10 in TGFβ induced invasion, we performed extracellular matrix (ECM) transwell invasion assays in A549 cells (Table 2). In RASSF10 knockout cells (ΔRASSF10) we found that TGFβ treatment promotes a significant induction of ECM invasion compared to mock treated cells (*p*-value ≤ 0.03). However, this induction was not significant (*p*-value ≥ 0.14) in the wtRASSF10 cells suggesting that depletion of RASSF10 enhances significantly the ability of A549 cells to invade an ECM.

#### 2.1.3. RASSF10 Is a Positive Regulator of TGFβ Repressed CDH1 Expression

**Figure 3:**
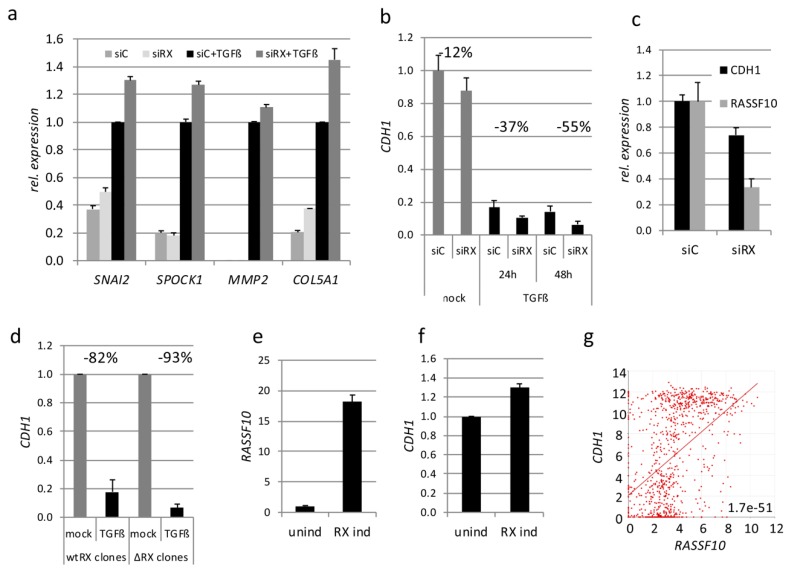
*RASSF10* (Ras Association Domain Family 10) and *CDH1* (Cadherin-1) expression are linked together. (**a**) Expression analysis shown for TGFβ induced genes upon *RASSF10* knockdown (siRX) and control knockdown (siC) by RT-PCR. Experiments were performed in A549 cells at 10 ng/ml TGFβ treatment vs. mock treatment (indicated time points or standard 48h), 72 h siRNA knockdown. Quantified RNA expression is normalized to *GAPDH*. Expression for control siRNA and TGFβ treatment was Set 1 for comparison. (**b**) Regulation of E-Cadherin (*CDH1*) expression by siRNA mediated *RASSF10* knockdown (siRX) and TGFβ treatment in A549 cells. (**c**) *CDH1* and *RASSF10* expression upon *RASSF10* knockdown (siRX) and control knockdown (siC). (**d**) CRISPR/Cas9 mediated RASSF10 loss (ΔRX clones) reduces CDH1 mRNA levels compared to RASSF10-expressing wildtype A549 clones (wtRXclones). (**e**) RASSF10 re-expression by a doxycycline inducible System (TetOn Invitrogen, Karlsruhe, Germany; 30 h) in HEK293 and according (**f**) RASSF10 driven induction of *CDH1* expression by RT-PCR. (**g**) Correlation analysis of CDH1 and RASSF10 expression in cancer (data CCLE Cancer Cell Line Encyclopedia; Broad; *n* = 917; log2 significance of correlation 1.7 × 10^−51^; analyzed using [28]).

Subsequently, we investigated the role of RASSF10 in TGFβ signaling, by *RASSF10* knockdown (siRX) during TGFβ treatment. TGFβ target genes were analyzed by RT-PCR (Figure 3a). *RASSF10* knockdown revealed that *RASSF10* inhibits induced TGFβ-target gene expression associated with extracellular matrix (*COL5A1*) and matrix metallopeptidase 2 (*MMP2*) or direct induction of EMT (*SNAI2* and *SPOCK1*) (Figure 3a). SNAI2 is a transcriptional repressor of E-cadherin (CDH1) and *CDH1* is downregulated upon TGFβ treatment (Figure 3b). Interestingly, we observed that *CDH1* levels are reduced by *RASSF10* knockdown (Figure 3b,c) and *CDH1* expression is significantly positively correlated with *RASSF10* expression (CCLE correlation analysis; Figure 3g). *RASSF10* deletion by CRISPR/Cas9 further reduced TGFβ driven *CDH1* repression (Figure 3d). *RASSF10* induction in HEK293 cells (lack of endogenous RASSF10) also led to an upregulation of *CDH1* mRNA levels (Figure 3e,f). This data suggests that RASSF10 is a positive regulator of TGFβ-repressed CDH1 expression. After studying the RASSF10 upstream regulation, we next focused on the identification of its interactome, as RASSF10 has no enzymatic activity itself.

### 2.2. RASSF10 Interacts and Stabilzes Apoptosis-Stimmlating of p53 Protein 2 (ASPP2) 

**Figure 4:**
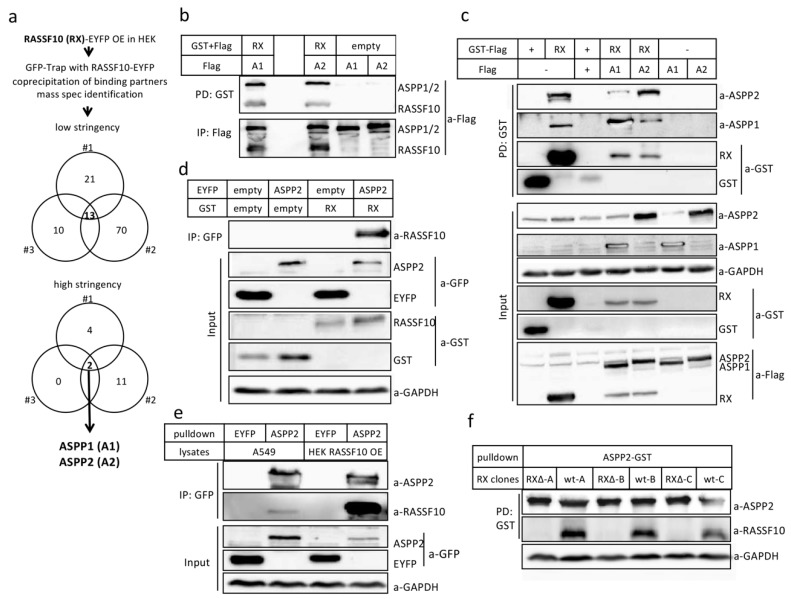
Identification of ASPP2 (Apoptosis-stimulating proteins of p53) as direct binding partner of RASSF10 (Ras-Association Domain Family 10). (**a**) Experimental set up for identification of binding partners of RASSF10 (RX) by IP and following mass spectrometry is shown. RASSF10 was overexpressed (OE) in HEK-cells and precipitated by green fluorescent protein GFP-Trap. RASSF10 binding partners were co-precipitated, separated in SDS-PAGE and MS identified. Under high stringency conditions we identified the ASPP1/2 (A1/2) as binding partners of RASSF10. Experiment was performed in biological triplicates. (**b**–**d**) Verification of the interaction of RASSF10 and ASPP1/2 in HEK-cells after overexpression of according constructs by pulldown (PD), SDS-PAGE and Western blotting is shown. (**b**) Pulldown of RASSF10 co-precipitates exogenous ASPP1 and ASPP2 and (**c**) endogenous ASPP1 and ASPP2. (**d**) Vice versa pulldown for ASPP2 co-precipitates RASSF10. ASPP2 was overexpressed in HEK cells, isolated and incubated with lysates from (**e**) wildtype A549-cells (expressing RASSF10) and (**f**) CRISPR/Cas9 mediated RASSF10 knockout A549 clones. Three RASSF10 deficient clones (Δ-A,B,C) and three wt clones (A,B,C) are shown.

For RASSF10 interactome analysis, we performed co-immuno-precipitations of overexpressed RASSF10 using the green fluorescent protein GFP-Trap system in biological triplicates (Figure 4a) and analyzed the RASSF10 partners by mass spectrometry (ESI). Results were normalized to likewise treated controls for each experiment (#1–#3) and we precipitated putative binding partners in overlap of three experiments (Table 3). Results came down to the apoptosis-stimulating proteins of p53 (ASPP1 and ASPP2) with a reproducible interaction with RASSF10 (Figure 4), in all three experiments under high stringency. It has been reported that ASPP2 controls epithelial plasticity and inhibits EMT through regulation of β-catenin and CDH1 [26]. 

#### 2.2.1. Verification of RASSF10 Interaction with ASPP1 and ASPP2

For confirmation we used co-precipitation by GFP-Trap and GST-glutathione pulldown for exogenous ASPP1/2-RASSF10 and endogenous ASPP1/2-RASSF10 interaction. RASSF10 was overexpressed and co-precipitated overexpressed ASPP1 and ASPP2 in Western blot (Figure 4b) and also endogenous ASPP1/2 (Figure 4c). RASSF10 co-localized with ASPP1 and ASPP2, whereas the latter was altered in its localization by RASSF10 (Figure S2). The interaction was further confirmed by co-precipitation of exogenous RASSF10 with GFP-trapped ASPP2 (Figure 4d). Interestingly, we observed that RASSF10 not only interacts with ASPP1/2 but also stimulates the endogenous levels of ASPP2 but not ASPP1 (Figure 4c; 5th panel lane 2). To test the interaction of ASPP2 with endogenous RASSF10 we used the RASSF10 expressing lung cancer cell line A549. We overexpressed and isolated ASPP2 by GFP-Trap in HEK, which was then incubated with A549 lysates or from HEK overexpressing Flag-RASSF10 (Figure 4e). We were able to detect co-precipitated endogenous RASSF10 in A549 approx. at the height of Flag-RASSF10 at 70 kDa. We used CRISPR/Cas9 to generate three RASSF10 negative clones in A549 (∆RX-A,B,C), which we controlled by PCR and sequencing the deletion within the coding region of *RASSF10* (Figure 1). Using three A549 RASSF10-negative clones and three RASSF10-positive wt clones (wt-A,B,C), we could show that knockout of endogenous RASSF10 abolishes its co-precipitation with ASPP2 (Figure 4f). Without prior co-precipitations/enrichment, we were unable to detect endogenous RASSF10. In our experiments, the RASSF10 ‘SDS PAGE-displayed MW’ is 70 kDa vs. its estimated 57 kDa size. The RASSF10 displayed size was verified with detected endogenous RASSF10 vs. loss of RASSF10 upon CRISPR/Cas9 knockout (Figure 4f). This detected difference in size can be due to its specific amino acid composition, post-translational modification and/or SDS (sodium dodecyl sulfate) occupancy in page [33,34].

#### 2.2.2. RASSF10 Stabilizes ASPP2 Protein through Its Coiled-Coils Domain

To characterize the ASPP1/2-RASSF10 interaction, we generated mutants of RASSF10 containing only its RA-domain (RASSF10D1-133) or its C-terminus with coiled-coils (RASSF10D237-508). The binding of ASPP to RASSF10/RASSF10-mutants was tested by co-precipitation (Figure S3a). Precipitation of ASPP1/2 was almost lost with RASSF10D1-133. Subsequently, we tested the strength of the ASPP-RASSF10 interaction by using RASSF10-mutants as competitors (Figure S3b). ASPP2 was co-precipitated with most of the domain mutants and competition with RASSF10D1-133 did not interfere with the binding of ASPP2 to wt-RASSF10 (Figure S3b). However, wt-RASSF10 can compete with RASSF10D237-362 regarding the binding to ASPP2 (Figure S3b). The vice versa experimental set up with GFP-trapped wt-RASSF10 that co-precipitates ASPP1/2, is not interfered with by the competitor RASSF10D237-362 (Figure S3c). For further competition experiments, we used the known ASPP binding partners YAP, p53 and p65 [35]. The strength of the ASPP-RASSF10 complex was further emphasized by the fact that YAP, p53 and p65 overexpression failed to compete with the co-precipitation of ASPP with RASSF10 (Figure S3d).

**Figure 5:**
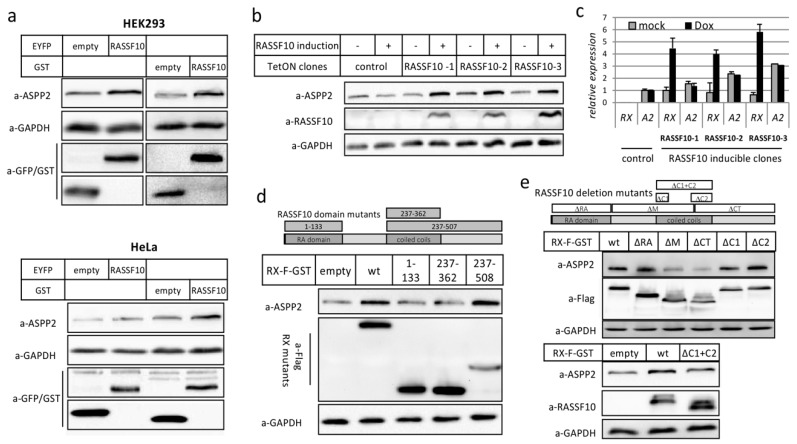
ASPP2 (Apoptosis-stimulating proteins of p53) is stabilized by RASSF10 (Ras Association Domain Family 10), coiled-coil domain dependently. (**a**) RASSF10 overexpression increased endogenous ASPP2 levels in HEK293 and HeLa. (**b**) RASSF10 induction (TetOn-TREx, Thermo Fisher Scientific, Karlsruhe, Germany) stabilizes ASPP2 levels in a doxycycline (+) RASSF10-inducible expression system. Three RASSF10-inducible clones RX-1, RX-2 and RX-3 and control clone are shown. (**c**) Equivalent experiment to b shows the unaffected transcriptional levels of ASPP2 upon RASSF10 induction by RT-PCR as normalized to GAPDH. (**d**) RASSF10 domain mutants (RASSF10D1-133, RASSF10D237-362, RASSF10D237-508) and (**e**) RASSF10 deletion mutants (∆RA, ∆M, ∆CT, ∆C1, ∆C2 and ∆C1+C2) were assessed for ASPP2 stabilization. After overexpression or induction of indicated vectors, protein lysates were isolated after 48 h, separated by SDS-PAGE and Western blotted with indicated antibodies.

Based on our initial observation that RASSF10 overexpression led to the stabilization of endogenous ASPP2, but not ASPP1 (Figure 4c), we verified that RASSF10 overexpression increases endogenous ASPP2 levels irrespective of the cell line or vectors used (Figure 5a). 

We created a RASSF10-inducible cell line (TetOn-TREx-System, Thermo Fisher Scientific) in HEK293 using doxycycline. Induction of RASSF10 strongly increased endogenous ASPP2 protein levels (Figure 5b), which was not due to increased *ASPP2* RNA expression (Figure 5c). Increased endogenous ASPP2 levels could also be shown by IF upon RASSF10 overexpression (Figure S2b). Next, we questioned which RASSF10 domain facilitates the ASPP2 stabilization. We used RASSF10-domain mutants (RASSF10D1-133, RASSF10D237-362, RASSF10D237-508) and deletion mutants (RASSF10∆RA, RASSF10∆M, RASSF10∆CT, RASSF10∆C1, RASSF10∆C2 and RASSF10∆C1+C2) (Figure 5d,e). The RA-domain or coils alone did not stabilize ASPP2 levels (Figure 5d). Deletion of RA-domain of RASSF10 or one coil retained the ability to stabilize ASPP2 (Figure 5e). In contrast, deleting the middle region of RASSF10 (∆M) or the C-terminus (∆CT) led to the loss of ASPP2 stabilization. Deletion of both coils almost completely abolishes ASPP2 stabilization (Figure 5e lower panel). Here we show that RASSF10 stabilizes ASPP2 through its coiled-coil domains. To understand the aberrant mechanism of the RASSF10-ASPP2 pathway which is relevant for human carcinogenesis, we investigated the genetic and epigenetic alteration of these genes in different tumor entities.

### 2.3. RASSF10 Is Inactivated across Human Cancers and a Valuable Cancer Biomarker

Tumor-suppressor gene inactivation can occur by loss of function mutation or promoter methylation [36,37]. We only found minimal genomic alterations of *RASSF10* (2.4%) and *ASPP2* (4.7%) compared to 62% of *TP53* mutations in cancer cell lines (*n* = 881; Broad CCLE/Cancer Cell Line Encyclopedia; analyzed using [38]). Similar mutation frequencies were found in primary tumors. *TP53* is heavily mutated in various cancers especially in lung (>80%), head and neck (>70%), colorectal (>50%), breast (>30%), kidney chromophobe (30%) and below 5% in kidney clear cell carcinoma. For *ASPP2* genetic mutations remain at very low levels with <1% in lung adenocarcinoma and kidney clear cell carcinoma and <2% in bladder cancer. For *RASSF10* there are no genetic alterations across primary cancers (TCGA/The Cancer Genome Atlas; analyzed using [39]). In summary, we are not convinced that mutations of *RASSF10* and *ASPP2* are likely to contribute to tumorigenesis, whereas occurrence of *TP53* mutations in cancer is consistent with literature [40].

#### 2.3.1. *RASSF10* Is Frequently Hypermethylated in Human Cancers

**Figure 6:**
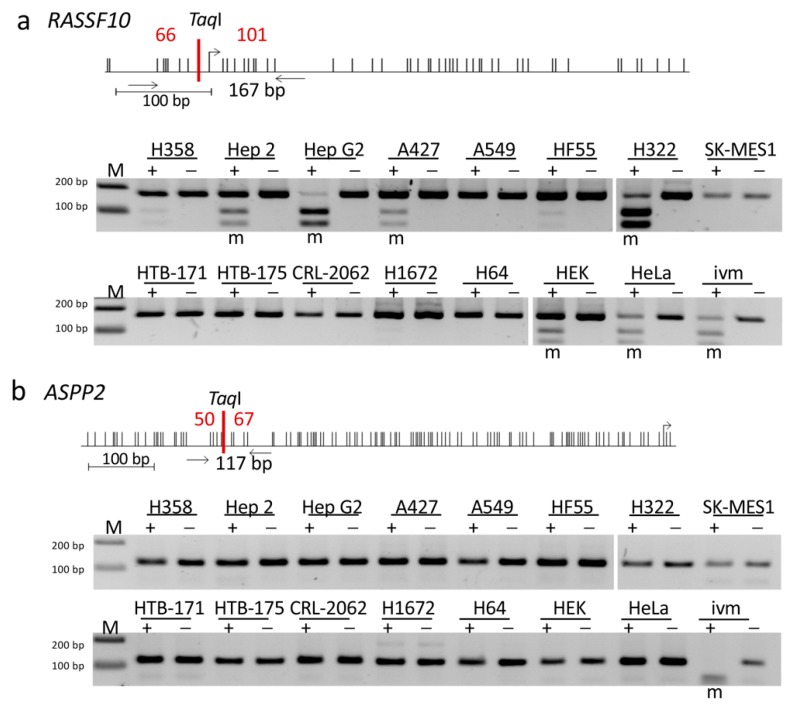
Epigenetic inactivation of RASSF10 (Ras Association Domain Family 10) but not ASPP2 (Apoptosis-stimulating proteins of p53) in cancer cell lines. Promoter inactivation by methylation was studied for the CpG islands of (**a**) *RASSF10* and (**b**) *ASPP2* in human cancer cell lines by COBRA (combined bisulfite restriction analysis). Upper panel shows schematic representation of the CpG island with transcriptional start sites (bent arrow), analyzed regions (horizontal arrows), single CpGs (black vertical lines), *Taq*I restriction sites (red) and resulting digestion products in bp (red). Lower panel shows 2% TBE agarose gels with digestion products, together with 100 bp marker (M). Abbreviations are methylated (m), digestion (+), mock digestion (−). An in vitro methylated (ivm) positive control was used.

**Figure 7:**
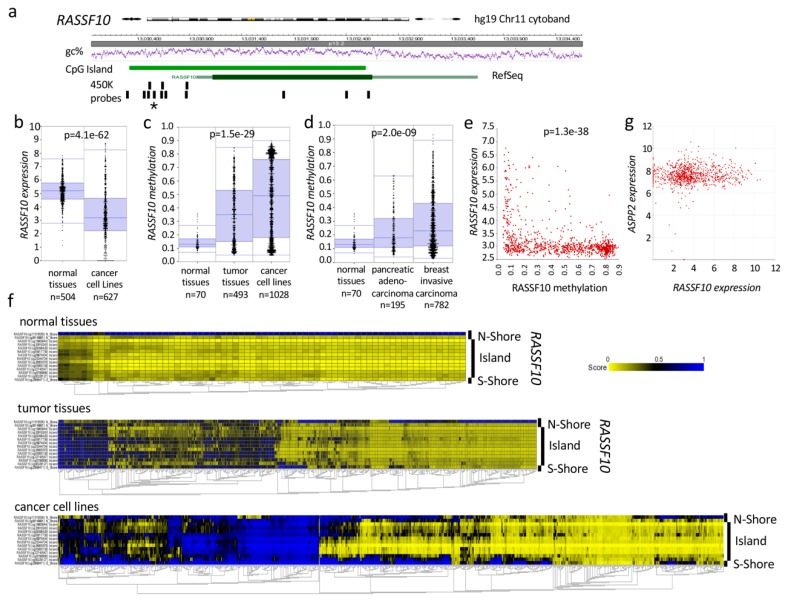
Epigenetic inactivation of RASSF10 (Ras Association Domain Family 10) across human cancers. (**a**) Genomic organization of *RASSF10* with CpG island and probe position of Illumina 450K methylation array (450K). Asterisk marks the depicted cg05817758 probe. (**b**) *RASSF10* expression is decreased in cancer cell lines vs. normal tissues (238755_at, log2, data Roth vs. Wappet, Anova one way). (**c**) *RASSF10* methylation increases from normal to tumor tissues and cancer cell lines (cg05817758, data Lokk vs. Heyn vs. Esteller, Anova one way). (**d**) Increased RASSF10 methylation in primary pancreatic adenocarcinoma and invasive breast carcinoma compared to normal tissue (cg05817758, data Lokk vs. TCGA vs. TCGA, Anova one way) (**e**) Loss of *RASSF10* expression correlates with its promoter hypermethylation (cg05817758 and 11742468_at, log 2 ps, data Esteller vs. Garnett). (**f**) Increased *RASSF10* promoter methylation (yellow unmethylated; blue methylated) is observed from normal to tumor tissues and cancer cell lines. Methylation is depicted relative to CpG island/shores and for all *RASSF10* (cg) reporters from 450K array. (**g**) *RASSF10* expression plotted relative to *ASPP2* expression across cancer cell lines (CCLE Cancer Cell Line Encyclopedia, log2, data Broad, n = 917).

*RASSF10* and *ASPP2* contain large CpG islands within their promoter regions (Figure 6, Figure 7 and Figure S4). In normal tissues, these both genes with CpG rich promoters are expressed (Figure 7 and Figure S4). We questioned whether the newly identified complex partners RASSF10-ASPP2 are regulated by promoter hypermethylation in cancer. We used the combined bisulfite restriction analysis COBRA methylation analysis, based on bisulfite conversion of unmethylated cytosins, for various cell lines: lung cancer cell lines: HTB171, HTB175, H64, H1672, CRL-2062 (small cell lung cancer); H358, A427, A549, H322 (non-small cell lung cancer); SK-MES-1 (squamous cell carcinoma); head and neck cancer Hep2, liver carcinoma HepG2, embryonal kidney HEK, cervix carcinoma HeLa and human fibroblasts HF55. We found that six out of 14 immortal cell lines showed methylation of *RASSF10* whereas HF55 was unmethylated. However, *ASPP2* was unmethylated in these cancer cell lines (Figure 6b). 

Genome wide analysis using publicly available datasets (*NCBI-GEO*; *Gene Expression omnibus*) verified our findings (Figure 7 and Figure S4). *RASSF10* is significantly hypermethylated in tumor tissues and cancer cell lines (*p* = 1.5 × 10^−29^; Figure 7c) but *ASPP2* is unmethylated (Supplementary Figure S4c). Increased methylation of RASSF10 was revealed in primary pancreatic adenocarcinoma and invasive breast carcinoma compared to normal tissue (*p* = 2.0 × 10^−9^; Figure 7d). Methylation of *RASSF10* occurs across the complete CpG island in tumor tissues and methylation is further increased in cancer cell lines, whereas normal tissues are unmethylated (Figure 7f). In contrast, *ASPP2* remains unmethylated in cancer across its CpG island (Figure S4e). *RASSF10* expression is significantly decreased in cancer cell lines vs. normal tissues (*p* = 4.1 × 10^−62^; Figure 7b), but not *ASPP2* (Figure S4b). As a causal correlation *RASSF10* promoter methylation significantly correlates with decreased expression in cancer cell lines (*p* = 1.3 × 10^−38^; Figure 7e), but not *ASPP2* (Figure S4d). *RASSF10* expression levels vary in cancer cell lines, but not the expression levels of *ASPP2* and expression of *RASSF10* and *ASPP2* do not correlate (Figure 7g).

#### 2.3.2. RASSF10 Inactivation Correlates with Clinical Diagnosis and Prognosis of Cancer

**Figure 8:**
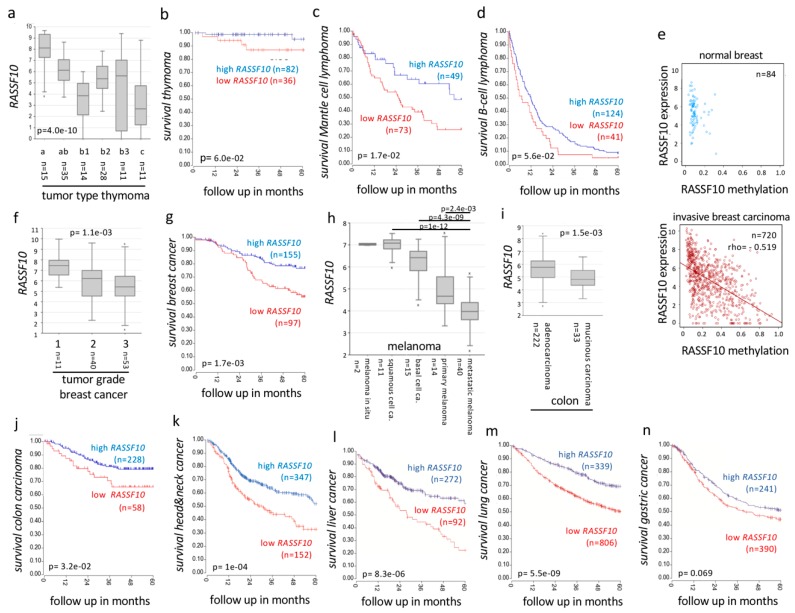
*RASSF10* (Ras Association Domain Family 10) inactivation correlates with clinical diagnosis and prognosis of human cancer. (**a**) Loss of *RASSF10* correlates with tumor type in thymoma (log2, data TCGA, Anova one way) and (**b**) Loss of *RASSF10* leads to reduced survival in thymoma (combined datasets). Loss of *RASSF10* correlates with reduced 5 year survival in (**c**) mantle cell lymphoma (data Staudt) or (**d**) B-cell lymphoma (data Xiao, subset: dead). (**e**) *RASSF10* expression is reduced by DNA methylation in breast invasive carcinoma vs. normal breast (cg05817758, beta value; expression log2; norm. rsem+1). (**f**) In breast cancer decreased *RASSF10* levels correlate with tumor grade (log2, data Clynes, Anova one way) and (**g**) correlate with reduced five year survival (data Bertucci). (**h**) In melanoma low *RASSF10* expression correlates with tumor type vs. metastatic melanoma (log2, data Matta). (**i**) In colon cancer *RASSF10* expression is reduced vs. adenocarcinoma (log2, data EXPO). (**j**) Relapse free survival was positively associated with *RASSF10* expression in colon carcinoma (data SieberSmith). In (**k**) head and neck cancer, (**l**) liver cancer, (**m**) lung cancer and (**n**) gastric cancer reduced RASSF10 expression is associated with reduced survival (KM plotter).

At last, we tested the ability of RASSF10 to serve as a prognostic and diagnostic biomarker in independent data sets for human neoplasia across various primary samples. In thymoma *RASSF10* expression was reduced with progressed tumor type and correlated with reduced survival (Figure 8b; Table S2). In lymphoma reduced *RASSF10* expression correlated with reduced survival (mantle cell) or an earlier death of patients (B-cell) (Figure 8c,d; Table S2). In breast cancer we can show that high *RASSF10* methylation is associated with reduced *RASSF10* expression, we observed that loss of *RASSF10* expression correlates with progressed breast cancer grade and reduced overall survival of breast cancer patients (Figure 8e–g, Table S2). Similarly, *RASSF10* expression was reduced in metastatic melanoma vs. non-metastatic melanoma types and in colon carcinoma vs. adenocarcinoma (Figure 8h,i, respectively). Reduced *RASSF10* levels further reveal a reduced survival of colon cancer (Figure 8j), head and neck cancer patients (Figure 8k), as well as liver cancer (Figure 8l), lung cancer (Figure 8m) and gastric cancer patients (Figure 8n). Supplementary Table S2 summarizes that low *RASSF10* expression is associated with reduced 5 year survival rates of cancers: kidney cancer, 24% (papillary, significant) and 12% (clear cell, significant); head and neck cancer, 18% (significant); lymphoma, 22% (mantle cell, significant) and 5% (B-cell); thymoma, 8%; liver cancer, 34% (significant); lung cancer, 18% (significant); gastric cancer, 7%; and breast cancer, 21% (significant). In summary, we observed that the levels of *RASSF10* expression/methylation are suitable for prognosis and diagnosis of various cancer types in humans. 

### 2.4. RASSF10 or ASPP2 Depletion Induces Activation of the TGFβ Signaling Pathway

**Figure 9:**
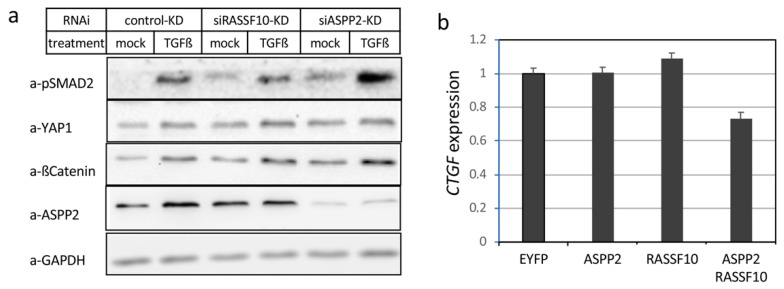
Knock down of *RASSF10* (Ras Association Domain Family 10) or *ASPP2* induces constitutive TGFβ (transforming growth factor beta) signaling. (**a**) Expression analysis shown for TGFβ induced signaling upon *RASSF10* knockdown (siRASSF10-KD), *ASPP2* knockdown (siASPP2-KD) and control knockdown (control-KD) by Western blot. Experiments were performed in A549 cells at 10 ng/ml TGFβ treatment vs. mock treatment (48 h), 72 h siRNA knockdown. Protein lysates were isolated after 48 h, separated by SDS-PAGE and Western blotted with indicated antibodies. (**b**) Combined RASSF10 and ASPP2 overexpression in HeLa cells reduces *CTGF* levels. Cells were transfected, RNA isolated after 48 h and qRT-PCR analysis was performed. *CTGF* expression was normalized to *GAPDH* levels and control transfection (EYFP Set = 1).

To analyze functional effects of RASSF10 and ASPP2 on TGFβ mediated signaling, we performed siRNA mediated knock-down (KD) and analyzed the molecular consequences on SMAD2 phosphorylation which is activated through the canonical TGFβ pathway (Figure 9). Knockdown of RASSF10 induced constitutive phosphorylation of SMAD2 at Ser465/467 and this constitutive activation of SMAD2 was also observed for the ASPP2 knockdown (Figure 9a). Interestingly, we found an enhanced phosphorylation of SMAD2 after TGFβ treatment and knockdown of ASPP2. Moreover, increased levels of ß-Catenin that represents another TGFβ target and a classical regulator of EMT were detected (Figure 9a). However, YAP1 levels were rather unaffected after RASSF10 or ASPP2 knockdown. Subsequently, we have analyzed the expression levels of CTGF that is a downstream effector of TGFβ signaling involved in EMT [41]. We revealed that overexpression of RASSF10 and ASPP2 reduces levels of CTGF in HeLa cells (Figure 9b). These data indicated that loss of RASSF10 or ASPP2 induced constitutive activation of TGFβ signaling and EMT.

## 3. Discussion

In our study, we wanted to understand the mechanism of regulation of RASSF10 and its contribution to inhibition of growth, migration and invasion as a tumor suppressor (Figure 1 and Table 2). TGFβ is well known for its inhibition of epithelial cell proliferation, however, during tumor progression cells evade the antitumoral TGFβ effect and TGFβ becomes oncogenic in late stage tumors by activation of EMT [32,42,43]. TGFβ signaling transmits an extracellular signal into a cellular/nuclear signal, with phosphorylation and nuclear translocation of SMADs followed by altered gene transcription (also SMAD independent effect) [44,45]. Here, we report that the RASSF10 tumor-suppressor is upregulated by TGFβ stimulation in epithelial cells (as well as by cellular density) and associated with a G1 cell cycle arrest restricting cell growth (Figure 2). However, the *RASSF10* promoter is not a directed SMAD target, since ChIP-sequencing (chromatin immune-precipitation) data indicate that SMAD2 and SMAD3 are not detected at the proximal *RASSF10* locus in several cell lines (Supplementary Figure S5). This suggests that TGFβ induced RASSF10 expression is a delayed gene response involved in downregulation of overshooting TGFβ signaling and regulation of the dual nature of TGFβ [46]. Loss of RASSF10 altered the TGFβ gene expression profile and induced expression of *COL5A1*, *MMP2*, *SNAI2* and *SPOCK1* (Figure 3a) and constitutive SMAD2 phosphorylation (Figure 9a), suggesting that RASSF10 plays a suppressive role in TGFβ signaling and EMT. SNAI2 and SPOCK1 are both factors that are known to trigger TGFβ induced EMT [4,47]. Additionally, we observed that depletion of RASSF10 significantly promotes TGFβ induced invasion of A549 cells in an extracellular matrix (Table 2). We presume that, during early TGFβ signaling expression of RASSF10 acts as a transmitter of antiproliferative TGFβ effects. Interestingly, we observed that RASSF10 induced E-cadherin (*CDH1*) levels and loss of RASSF10 expression reduced *CDH1* levels (Figure 3). CDH1 is essential for the maintenance and homeostasis of polarized epithelial monolayers [48] and its transcription is repressed through the TGFβ induced expression of SNAI2 [3]. We revealed, that *RASSF10* expression positively correlated with the expression of *CDH1* (Figure 3g) and GO terms associated with the plasma membrane, cell surface receptor signaling and epithelial/epidermis cell differentiation (Table 1). The role of RASSF10 was further supported by finding its interaction partner ASPP2 (Table 3 and Figure 4) that also controls epithelial plasticity through regulation of CDH1 [26]. The strength of the ASPP-RASSF10 interaction was further emphasized by the fact that known ASPP2 binding factor YAP1 [49], p53 and p65 overexpression [50] failed to compete with the co-precipitation of ASPP with RASSF10 (Figure S3d). There are several data supporting the ASPP2 role at the cell periphery through the cell polarity complex PAR [51,52,53]. Our findings regarding the cellular role and localization of RASSF10-ASPP2 are further supported by interactome analysis, in which ASPP2 and RASSF10 are placed together in a large interaction module that provides links to cell polarity [54]. Additionally, ASPP2 forms an apical-lateral polarity complex at the level of tight junctions in polarized epithelial cells, acting as a scaffold for, e.g., protein phosphatase 1 (PP1) and junctional YAP [55] and ASPP2 is also said to regulate epithelial plasticity through CDH1 and β-Catenin regulation [26]. Our data indicate that knockdown of ASPP2 induces β-Catenin levels and phospho-SMAD2 under TGFβ treatment (Figure 9a). Previously it has been reported that ASPP2 suppresses TGFβ induced EMT by inhibiting Smad7 degradation [27]. SMAD7 prevents SMAD2/3 phosphorylation and is an important antagonist of TGFβ signaling [56]. In summary, we presume that RASSF10 induces ASPP2 and ASPP2 inhibits degradation of SMAD7 that counteracts TGFβ signaling and EMT. Moreover, RASSF10 and ASPP2 overexpression reduced levels of the matricellular protein CTGF (CCN2). CTGF has an important growth promoting role in cancer and is also involved in angiogenesis, cell adhesion and migration [57]. We found that knockdown and knockout of RASSF10 increased mitosis and increased cell proliferation (Figure 1) and significantly promotes TGFβ induced ECM invasion (Table 2), as well as RASSF10 re-expression halted proliferation (Figure 1) [19,22]. Additionally, RASSF10 was found at centrosomes/microtubules during mitosis [58]. In accordance ASPP2 was said to be involved in centrosome linker assembly at the end of mitosis [59]. As RASSF10 and ASPP2 [26,51,52,53,54,55] are both linked to the cell polarity network, their presence would negatively regulate the mitotic/proliferative potential. Loss of the RASSF10-ASPP2 complex would lead to disturbance of cell polarity and thereby would interfere with the coordination of mitosis, as it is known that the positioning of the spindle apparatus is coordinated with polarity signals at the cell cortex [60]. The disruption of cell polarity itself is regarded as a central hallmark of cancer [61]. 

In our study we found a significant epigenetic silencing of *RASSF10*, but not *ASPP2* in different tumor entities (Figure 6 and Figure 7). Promoter hypermethylation of tumor suppressor genes is an established mechanism of their silencing in carcinogenesis [62]. *RASSF10* methylation increased from primary tumors to cancer cell lines (Figure 6), consistent with the progressive hypermethylation of tumor-suppressors during tumorigenesis [63,64]. In our previous work, we reported that the pharmacological inhibition (e.g., 5-Aza-2’-deoxycytidine) of DNA methylation restored *RASSF10* expression in different cancer entities [17,19,22,65]. We observed that methylation levels of *RASSF10* correlated significantly with its reduced expression in cancer (Figure 7e). Our clinical data set revealed that hypermethylation of the CpG island of *RASSF10* is a common and general event in human tumorigenesis (Figure 7 and Figure 8). We could broadly show that in independent data sets loss of *RASSF10* correlated not only with reduced patient survival rates in various tumor types (kidney cancer, thymoma, lymphoma, breast cancer, colon carcinoma, head and neck cancer, liver cancer, lung cancer and gastric cancer), but also with tumor stage/grade (thymoma, breast cancer) and tumor types (kidney cancer, melanoma and colon carcinoma) (Figure 8 and Table S2). Further analyses will become possible, when newer data sets are available in which *RASSF10* is now commonly integrated. Our present comprehensive work is the finalization of our previous research in smaller data sets in which we have shown that the promoter of *RASSF10* is methylated in patient tumors samples of the adrenal gland [21], head and neck [19], sarcoma [19], pancreas carcinoma [19] and Merkel cell carcinoma [20]. We showed the epigenetic inactivation of *RASSF10* in thyroid cancer [17], lung cancer [19], skin cancer [65], breast cancer [22] and showed that *RASSF10* inhibited growth of breast cancer [22], pancreas carcinoma and sarcoma cell lines [19]. Here, we now confirmed the prognostic and diagnostic value of *RASSF10* across tumor types (Figure 8). As *RASSF10* DNA promoter methylation correlates with its RNA expression across cancers (Figure 7e), we are confident that measuring RASSF10 methylation represents its expression profile. It is obvious to measure methylated tumor DNA instead of RNA due to its superior stability in cells and body fluids (circulating DNA). Furthermore, *RASSF10* RNA levels vary in tissues, and determining the according threshold level of inactivation would have to be determined for each tissue or cancer type. However, in DNA, we observed that already low levels of methylation inactivated RASSF10 and therefore a common threshold level could abrogate *RASSF10* expression irrespective of the tissue. Tumor DNA could be obtained from tumor resections/biopsies or also non invasive by liquid biopsies [66], where circulating tumor cells (CTC) or circulating tumor DNA (ctDNA) are present in blood/body fluids or even from exhaled breath condensates (EBC) [67]. It was reported that in ex-smokers hypermethylation of RASSF1A could be measured in EBC [68] and EBCs are in clinical trials [69]. Liquid biopsies are FDA approved for lung cancer EGFR mutation tests as companion diagnostic [70]. Bisulfite treatment of DNA still is the gold standard to analyze DNA methylation [71], high-throughput bisulfite conversion is available [72] and digital droplet PCR (ddPCR) even amplifies low levels of nucleic acid in disproportionate sample/target combinations [73]. RASSF10 methylation analysis should be evaluated for its integration in present cancer screens.

## 4. Materials and Methods 

### 4.1. Cell Culture and Treatment of Cell Lines 

Cell lines were grown in appropriate medium (DMEM/Dulbecco’s Modified Eagle’s Medium, RPMI/Roswell Park Memorial Institute) supplemented with 10% FCS/fetal calf serum, 1% Penicillin/Streptomycin under cell culture conditions (37 °C, 5% CO_2_). Cell lines were transfected for indicated time points using Polyethylenimmin (PEI, 4,9mM, Sigma, St. Louis, MO, USA) for HEK/HeLa, Turbofect (Thermo Fisher Scientific) or X-tremeGENE HP (Roche, Mannheim, Germany) for A549 according to manufacturer’s protocol. Doxycycline (Dox, Thermo Fisher Scientific) was dissolved in water and used for RASSF10 induction in HEK-cells within the TetOn-TREx-system at working concentration of 2 µg/mL for 48 h [74]. SiRNAs (RASSF10 and ASPP2) were purchased from Dharmacon (Lafayette, CO, USA) On-target plus siRNA pool of four and was transfected using Lipofectamine RNAiMAX (Thermo Fisher Scientific). TGFβ (pH3 Citrate buffer) was used at 10 ng/ml. For flow cytometry assay overnight ethanol fixed cells were *RNase* A (50 µg/mL, 30 min, 37 °C) treated, stained with 50 µg/mL propidium iodide prior to measurement of DNA content in FACSCantoII (BD Biosciences). FACSDiva Software (BD Biosciences) was used for measurement and gating to distinguish cell cycle phases. 

### 4.2. Generation of Stable RASSF10 Inducible HEK-Cells 

HEK-cells stably expressing the Tet Repressor under Blasticidine (5 µg/mL, Roth, Karlsruhe, Germany) were obtained from Thermo Fisher Scientific as part of the TetOn-TREx-System and were used as control cell line. These cells were transfected with the cloned RASSF10-pcDNA4ToMyc for stable insertion of Dox-inducible-RASSF10. Cells were selected for RASSF10 using Zeocin (500 µg/mL, Thermo Fisher Scientific). Three clones with stable Dox-inducible-RASSF10 were used in our experiments. 

### 4.3. Mass Spectrometry and Co-immunoprecipitations/Pulldown assays and Western Blotting

For identification of RASSF10 binding partners we used the reverse-phase nano liquid chromatography coupled with mass spectrometry at Marcus Krueger’s lab. We overexpressed RASSF10-EYFP and EYFP empty in HEK-cell for 24 h. RASSF10-EYFP and EYFP alone were precipitated from lysates using GFP-Trap (Chromotek, München, Germany) according to manufacturer’s protocol. Samples were separated via NuPAGE (Thermo Fisher Scientific), Coomassie stained and gel lanes were cut in equal sized fragments. Proteins in gel pieces were then reduced, alkylated, digested with Trypsin, eluted and peptides were loaded onto C18 reversed phase HPLC columns for following analysis. Peptides were ionized by electrospray ionization and transferred into the mass spectrometer. The method was described earlier [75,76]. For further interaction analyzes of RASSF10 with its binding partners we used pulldown/co-immunoprecipitations. Plasmids were overexpressed in indicated cell lines and whole cell lysates were prepared at indicated time points. Lysates were incubated with Glutathione-Sepharose 4B (GE Healthcare Life Sciences, Solingen, Germany), Anti-Flag M2 Affinity Gel (Agarose) (Sigma) or GFP-Trap_agarose (Chromotek) and pulldowns were performed according to manufacturer’s protocols. Precipitates were separated via SDS-PAGE and Western blotted onto PVDF membrane (Immobilon, Merck, Darmstadt, Germany) for antibody based detection. Luminata Crescendo Western HRP substrate (Millipore, Merck, Darmstadt, Germany) was used for detection at VersaDoc Imaging System (BioRad, Hercules, United States). The following antibodies were used: a-GAPDH (FL335, sc-25778 from Santa Cruz), a-GFP from Rainer Renkawitz (Giessen, Germany), a-RASSF10 (AP12444c-ev2020, Abgent, San Diego, United States), a-p65 (610868, BD Biosciences, Heidelberg, Germany), a-ASPP1 (sc50890, Santa Cruz, Santa Cruz, United States), a-ASPP2 (sc53861, Santa Cruz), a-YAP1 (sc15407, Santa Cruz), a-ß-Catenin (9562S, Cell Signaling), a-phospho-SMAD2 ser465/467 (3101, Cell signaling), a-Flag (M2, Sigma), a-GST (B-14 sc138, Santa Cruz), a-V5 Tag (ab9116, Abcam, Cambridge, United Kingdom), HRP-coupled secondary antibodies anti-rabbit (sc2004, sc2357, Santa Cruz), anti-mouse (sc2005, sc516102, Santa Cruz) and anti-goat (sc2021, Santa Cruz), alexafluor568 (Thermo Fisher Scientific).

### 4.4. DNA Isolation and Methylation Analysis

The promoter region of *RASSF10* and *ASPP2* was analyzed by CpG plot (EMBL-EBI, Hinxton, United Kingdom) and both show large CpG islands. Primers for bisulfite treated DNA were designed to bind only fully converted DNA and amplify promoter region (Table S3). The precise promoter region was chosen for CpG content and presence of according restriction enzymes for COBRA analysis (Figure 6). DNA was isolated after proteinase K (Thermo Fisher Scientific) digest and extracted either with phenol/chloroform or by QIAamp DNA extraction kit (Qiagen, Hilden, Germany), and concentrations were determined by UV-photospectrometery. For COBRA methylation analysis 2 µg genomic DNA was bisulfite treated (5 mM hydroquinone, 1.65 M sodium metabisulfite and pH 5.5 with 0.025M NaOH) and incubated over night at 50 °C. DNA was purified using MSB Spin PCRapace (STRATEC Molecular, Birkenfeld, Germany), eluted in 50 µL H_2_O and followed by 10 min incubation with 5 µL 3 M NaOH at 37 °C. DNA was then precipitated with 100% ethanol and 7.5 M ammonium acetate and resolved in 1 × TE buffer. The subsequent PCR product (COBRA primers) was digested with 0.5 µl of *Taq*I (Thermo Fisher Scientific) 1 h at 65 °C and resolved on 2% TBE gel together with mock control and DNA ladder. The COBRA product for *RASSF10* is 167 bp (*Taq*I site at 66 bp) for semi-nested PCR and the product for ASPP2 is 117 bp (*Taq*I site at 50) for nested PCR. In vitro methylation of genomic DNA was performed using CpG Methyltransferase *M.Sss*I (NEB, Frankfurt, Germany) according to manufacturer’s protocol. For Primers see Supplementary Table S3. For further details on COBRA analysis see Richter et al. [22].

### 4.5. RNA Expression Analysis 

RNA was isolated from human cell culture or mouse primary tissues (homogenized using Bioruptor, Diagenode, Seraing, Belgium) using Isol-RNA lysis procedure (Trizol, Thermo Fisher Scientific). RNA was *DNase* (Thermo Fisher Scientific) treated and then reversely transcribed by MMLV (Promega, Walldorf, Germany). Quantitative RT–PCR was performed in triplicate with SYBR select (Thermo Fisher Scientific) using Rotor-Gene 3000 (Qiagen, Hilden, Germany). For Primers see Supplementary Table S3. 

### 4.6. Plasmids

RASSF10’s coding sequence was amplified from genomic DNA, ASPP1 (IRAVp968F02130D) and ASPP2 (IRATp970A06136D) were obtained from the former *Deutschen Ressourcenzentrum für Genomforschung* (RZPD) now THE I.M.A.G.E. Consortium. Necessary site directed mutations were generated using QuikChange Lightning (Agilent, Santa Clara, United States) and coding sequences were cloned into according expression vectors: pCMVTag1 with Flag-Tag (Agilent, Santa Clara, United States), pEYFP and mCherry (Clontech, Mountain View, Germany), pEBG (GST and Flag, Addgene, Watertown, United States), and pcDNA4/To/Myc from the inducible system T-REx (Thermo Fisher Scientific). Plasmids were controlled by sequencing, expression analysis by RT-PCR, Western blotting and immunofluorescence. Susanne Maaz cloned p53 (IRALp962F088Q) into pCDNA3.1/nV5-DEST (Thermo Fisher Scientific) and YAP1 (IRAKp961L0779) was cloned into EYFP (Clontech) by Desiree Block and A.J. p65-pcDNA3 was a gift from Lienhard Schmitz (Giessen, Germany). Deletion mutants and domain mutants of RASSF10 were generated by site directed mutations as above. RASSF10 domain (D) mutants are RASSF10D1-133 (RA-domain), RASSF10D237-362 (coiled-coils), RASSF10D237-507 (coiled-coils and C’ terminus) and deletion mutants of RASSF10 are RASSF10∆RA (deletion: 4-133), RASSF10∆M (deletion: 133-318), RASSF10∆CT (deletion: 319-507), RASSF10^∆C1^ (deletion: 237-264), RASSF10∆C2 (deletion: 315-362) and RASSF10∆C1+2 (deletion: 237-362). 

### 4.7. CRISPR/Cas9

To genomically delete *RASSF10* we performed CRISPR/Cas9 targeted knockout. CRISPR/Cas9 vectors were obtained from Lienhard Schmitz (Giessen, Germany) and *RASSF10* targeting oligos/guideRNAs were generated according to protocol [77]. *RASSF10* knockout oligos (two combinations) were created to delete a region 3 prime of the translational start site, creating a frameshift and RASSF10 loss in p × 549 with wt Cas9. We transfected A549-cells with the CRISPR/Cas9 RASSF10 oligos and selected for positive clones by puromycin (1 µg/mL) for three days. Clones were expanded and the knockout was verified by PCR based amplification of the *RASSF10* genomic region and RNA showing the deletion and was further verified by Western blot. 

### 4.8. Invasion Assay 

For the evaluation of the invasion capacity of A549 wildtype and RASSF10 deletion clones transwell extracellular matrix (ECM) invasion assays were performed. In 24-well plate a volume of 750 µL of DMEM with 10% FCS and 5 ng/mL TGFβ (or mock) was distributed into the wells. Filter inserts (8.0 µm pore size, Falcon cell culture insert, transparent PET membrane, Corning, Kaiserslautern, Germany) were coated with 100 µL ECM (0.25 mg/mL Matrigel Matrix, Corning). 5 × 10^4^ cells were seeded in 200 µL DMEM with 5 ng/mL TGFβ (or mock) and incubated at 37 °C with 5% CO_2_ for 16 h. Cells were fixed with 3.7% formaldehyde and 100% methanol for 2 min. Finally, cells were stained with 10% Giemsa for 15 min, washed with PBS and ECM was removed with a cotton swab. Bottom side of membranes were analyzed by microscopy (Motic AE21, Wetzlar, Germany) and all invasive cells were counted.

### 4.9. Statistical Analysis

Gene Expression, promoter methylation correlation and Kaplan Meier calculations were performed using *R2 Genomics Analysis and Visualization Platform* [28], *Wanderer* [78]*, KM Plotter* [79,80,81,82] and *MethSurv* [83]. For further calculations we used *GraphPad.* For details please see Appendix A. 

## 5. Conclusions

Cancer incidences are still growing and thereby the need for precision medicine and novel targeted therapies is rising. Our work directly serves this demand to identify for novel cancer targets. We are now showing that RASSF10 expression inhibits signs of EMT in cancer cell lines, but we are also reporting its mode of action as a tumor suppressor. RASSF10 is regulated by TGFβ signaling and promoter hypermethylation and RASSF10 itself modulated TGFβ signaling through its regulation of epithelial cadherin. Depletion of RASSF10 promotes TGFβ induced ECM invasion and constitutive SMAD2 phosphorylation. We identified ASPP2 (apoptosis-stimulating protein of p53 2) as the main binding target of RASSF10 by interactome analysis, which depended on the RASSF10 coiled-coils domain. We show that RASSF10 induces the protein levels of ASPP2 that is also an inhibitor of EMT. In human cancers, RASSF10, but not ASPP2, is epigenetically inactivated by promoter hypermethylation. In independent datasets we could validate, that loss of RASSF10 expression clinically correlated with decreased survival and with progressed disease state of cancer patients. Thus, loss of RASSF10 expression by promoter hypermethylation can serve as a diagnostic and prognostic cancer marker.

## Figures and Tables

**Table 1:** GO (Gene ontology) term analysis of RASSF10-associated genes in cancer cell lines [28].

GO Term	GOPath	*p*-Value
cell periphery	GO:0071944	1.2 × 10^−32^
plasma membrane	GO:0005886	7.1 × 10^−31^
epidermis development	GO:0008544	1.5 × 10^−30^
skin development	GO:0043588	4.5 × 10^−30^
membrane part	GO:0044425	8.4 × 10^−28^
epidermal cell differentiation	GO:0009913	1.3 × 10^−25^
epithelial cell differentiation	GO:0030855	1.4 × 10^−24^
apical plasma membrane	GO:0016324	8.7 × 10^−18^
cell–cell junction	GO:0005911	5.0 × 10^−16^

**Table 2:** Summary of extracellular matrix transmembrane invasion assay of wtRASSF10 and ΔRASSF10 cells.

A549 Clone	Treatment	Mean Number of Invasive Cells (± SD, *n* = 3)	Fold Induction (*p*-Value) ^a^
A ΔRASSF10	5 ng/mL TGFβ	523 (±237)	870 (0.02)
A ΔRASSF10	mock	0.3 (±0.6)	
B ΔRASSF10	5 ng/mL TGFβ	727 (±363)	15 (0.03)
B ΔRASSF10	mock	48 (±73)	
C ΔRASSF10	5 ng/mL TGFβ	59 (±27)	197 (0.02)
C ΔRASSF10	mock	0.3 (±0.6)	
A wtRASSF10	5 ng/mL TGFβ	14 (±10)	1.2 (0.87)
A wtRASSF10	mock	12 (±15)	
B wtRASSF10	5 ng/mL TGFβ	15 (±13)	21 (0.14)
B wtRASSF10	mock	0.7 (±1.2)	

^a^ Two tailed t-test (TGFβ vs. mock treatment), Δ (deletion), RASSF10 (Ras Association Domain Family 10), TGFβ (Transforming growth factor beta).

**Table 3:** RASSF10 interacting proteins identified my mass spectrometry.

No	Protein ID	Enrichment Above Control	Full Protein Name
IP	RASSF10	286	Ras-association domain family 10
1	ASPP1	185	Apoptosis-stimulating of p53 protein 1
2	ASPP2	184	Apoptosis-Stimulating of p53 protein 2
3	SMU1	70	WD40 repeat-containing protein SMU1
4	EHD4	50	EH-domain containing 4
5	SDC2	42	Syndecan 2
6	PLEKHA5	42	Pleckstrin Homology Domain Containing, Family A Member 5
7	PTPN13	32	Protein tyrosine phosphatase, non-receptor type 13
8	HRC1	31	HRAS1-related cluster protein 1
9	CASK	25	Calcium/calmodulin-dependent serine protein kinase
10	NID1	23	Nidogen 1
11	DLG1	18	Discs, large homolog 1 (Drosophila)
12	PPP1CA	17	Protein phosphatase 1, catalytic subunit, alpha isoform
13	FBXW11	16	F-box and WD-40 domain protein 11

## Supplementary Materials

The following are available online at https://www.mdpi.com/2072-6694/11/12/1976/s1, Figure S1: Structure of RASSF10., Figure S2: RASSF10 (RX) localizes with overexpressed ASPP1/2 (A1/A2) and induces endogenous ASPP2, Figure S3: Characterization of RASSF10 binding to ASPP1/2 and competition; Figure S4: *ASPP2* is not epigenetically regulated in human cancers; Figure S5: No SMAD2 and SMAD3 binding at the RASSF10 promoter. Table S1: RASSF10 negatively and positively associated genes that are under- and overrepresented in the gene set hallmark EMT; Table S2: 5-year cancer patient survival *RASSF10* expression dependent, Table S3: List of primers.

## Appendix A. Additional Information on Datasets and Software Used in this Study

The information is listed in order of its appearance. Analysis performed using the R2 [28] *Genomics Analysis and Visualization Platform*.

Figure 2 and Figure 3: TGFβ time series A549, select gene: RASSF10 238755_at, dataset set_public_u133p2, raw values expression vs. time in hours, A549-GSE17708_‘Time Course of TGF-beta treatment of A549 lung adenocarcinoma cell line’ [84], 5 ng/mL TGFβ, triplicate, Affymetrix HG_133_plus_2 array; Expression correlation: Cellline CCLE Cancer Cell Line Encyclopedia - Broad - 917 - MAS5.0 - u133p2, correlate two genes, log2, RASSF10 (238755_at) APS=91.7(445) Avg = 47.6 and CDH1 (201131_s_at) APS = 1694.8(512) Avg = 947.9, Significance of correlation: r-value = 0.470 *p*-value = 1.75 × 10^−51^ T-value = 16.090 degrees of freedom=915, adjust: linear fit, Source: GEO ID: gse36133 Dataset Date: 2012-03-20; Ref [85]; TGFβ stimulation of HMEC: GEO GDS4071, Platform: GPL570: [HG-U133_Plus_2] Affymetrix Human Genome U133 Plus 2.0 Array, Title: ‘TGFβ1 effect on HMEC-TR mammary epithelial cell line deficient in Smad4 or TIF1γ’, Ref [86];

Figure 7 and Supplementary Figure S4: Normal Various - Roth - 504 – MAS5.0 - u133p2, Source: GEO ID: gse7307 Dataset Date: 2007-04-09; Cellline Cancer AstraZeneca - Wappet - 627 - MAS5.0 - u133p2, Source: GEO ID: gse57083 Dataset Date: 2014-04-28; Normal Tissues - Lokk - 70 - custom - ilmnhm450, Source: GEO ID: gse50192 Dataset Date: 2014-02-26; Tumor Types (landscape) - Heyn - 493 - custom - ilmnhm450, Source: GEO ID: gse76269 Dataset Date: 2017-06-07; Cellline Cancer Pharmacogenomic - Esteller - 1028 - custom - ilmnhm450, Source: GEO ID: gse68379 Dataset Date: 2016-07-05; Cellline Cancer Drug (Sanger) - Garnett - 1017 - rma - u219, Available on R2 since: 2016-08-05;

Figure 7: T-SNE on Broad and Roth, Transform: zscore, no gene filter, no sample filter, perplexity = 23, Color mode: Color by Gene (RASSF10/ASPP2), Transform for Track Gene: none; Cellline CCLE Cancer Cell Line Encyclopedia - Broad - 917 - MAS5.0 - u133p2, Source: GEO ID: gse36133 Dataset Date: 2012-03-20;

Figure 8: Tumor Thymoma - TCGA - 120 - rsem - tcgars, Source: TCGA ID: THYM, Available on R2 since: 2016-03-08, RASSF10 (RASSF10_644943) APS=123.7258(118) Avg = 121.7, Clinisnitch: histologic_diagnosis, Sample Filter Select track: histologic_diagnosis type; Tumor Mantle cell lymphoma - Staudt - 122 - MAS5.0 - u133p2, Source: GEO ID: gse93291 Dataset Date: 2017-03-17, RASSF10 (238755_at) APS = 18.86(26) Avg = 11.7, cutoff_modus: scan, follow up threshold: 60 months; Tumor B-cell Lymphoma - Xiao - 420 - MAS5.0 - u133p2, Source: GEO ID: gse10846 Dataset Date: 2008-11-28, RASSF10 (238755_at) APS = 17.2(66) Avg = 9.8, cutoff_modus: scan (and minimal sample size in one group 20%), follow up threshold: 60 months, Sample Filter Select track: follow_up_status dead; Mixed Tumor Breast - Clynes - 121 - MAS5.0 - u133p2, Source: GEO ID: gse42568 Dataset Date: 2013-05-26, RASSF10 (238755_at) APS = 154(85) Avg = 114.9, Clinisnitch: grade, Sample Filter Select track: grade; Tumor Breast (MDC) - Bertucci - 266 - MAS5.0 - u133p2, Source: GEO ID: gse21653, Available on R2 since: 2014-09-05, RASSF10 (238755_at) APS = 76.4(212) Avg = 62.7, cutoff_modus: scan, follow up threshold: 60 months; Tumor Melanoma (Metastatic) - Matta - 87 - MAS5.0 - u133p2, Source: GEO ID: gse7553 Dataset Date: 2008-05-28, RASSF10 (238755_at) APS = 73.39(62) Avg = 56.4, Clinisnitch: type, Sample Filter Select track: type; Tumor Colon - EXPO - 315 - MAS5.0 - u133p2, Source: GEO ID: GSE2109 Dataset Date: 2005-01-15, RASSF10 (238755_at) APS=67.7(268) Avg = 60.7, Clinisnitch: histology, Sample Filter Select track: histology; Tumor Colon - SieberSmith - 355 - MAS5.0 - u133p2, Source: GEO ID: GSE14333, GSE17538 Available on R2 since: 2011-03-30, RASSF10 (238755_at) APS = 90(337) Avg = 86.1, cutoff_modus: scan (and minimal sample size in one group 20%), follow up threshold: 60 months, relapse free survival probability. Analysis performed using the Wanderer [78] TCGA data.

Figure 8: Methylation Expression Correlation: RASSF10, Dataset Project: TCGA, Data Type: 450k Methylation Array 450k Infinium chip, Select: cg05817758, beta value and log2 (normalized rsem+1) of RASSF10, Correlation method: Spearman, Fit linear regression, for Breast invasive carcinoma. Analysis performed using the KM Plotter [79,80,81,82]. Thymoma, Head-neck squamous cell carcinoma; mRNA Affymetrix ID 238755_at: Liver cancer, lung cancer, gastric cancer, Split patients by: Auto select best cutoff, Follow up threshold: 60 months. Analysis performed using MethSurv [83].
