# Supplementary material for: RASSF10 Is a TGFβ-Target That Regulates ASPP2 and E-Cadherin Expression and Acts as Tumor Suppressor That Is Epigenetically Downregulated in Advanced Cancer
Source: Cancers (Basel). 2019 Dec 8;11(12):1976. doi: 10.3390/cancers11121976 (PMC6966473; doi:10.3390/cancers11121976)

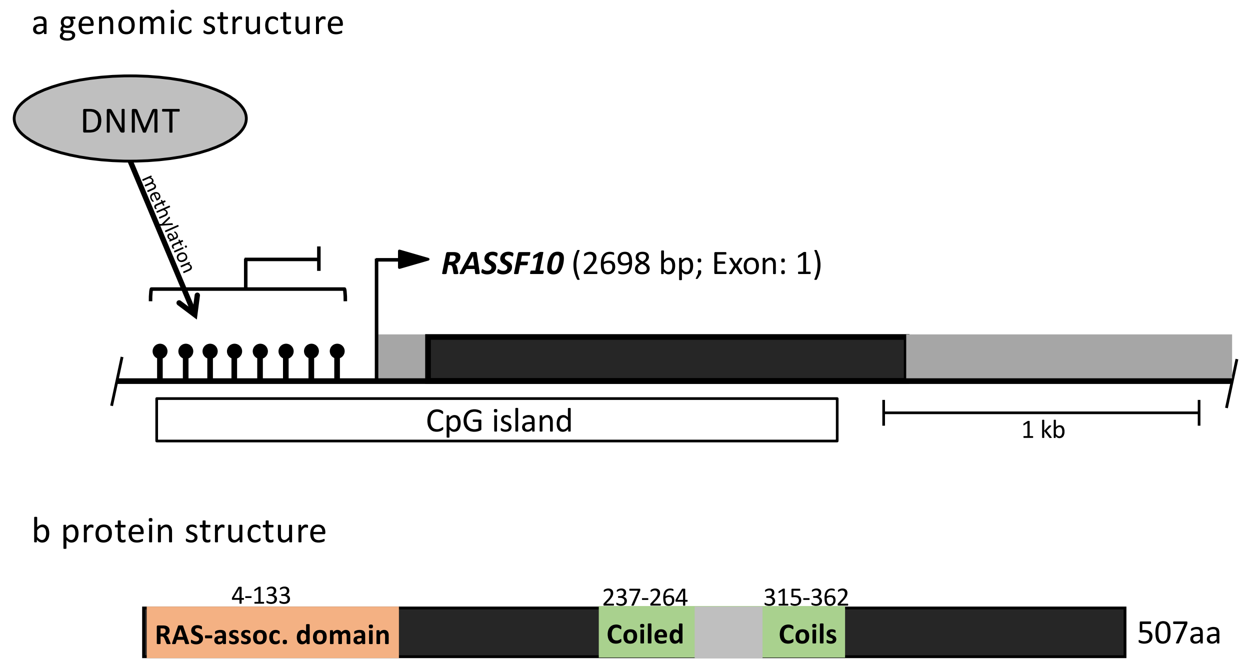

Supplementary Figure S1. Structure of RASSF10. (a) RASSF10 is transcribed from one exon into a 2.7 kb mRNA with a 1.5 kb coding sequence (black). In cancer, RASSF10 is subject to hypermethylation of its CpG island promoter through DNA-methyltransferases (DNMTs), thereby blocking its transcription in cancer. (**b**) RASSF10 (507 aa) contains an N-terminal RAS-association domain (red) and central coiled coils (orange).

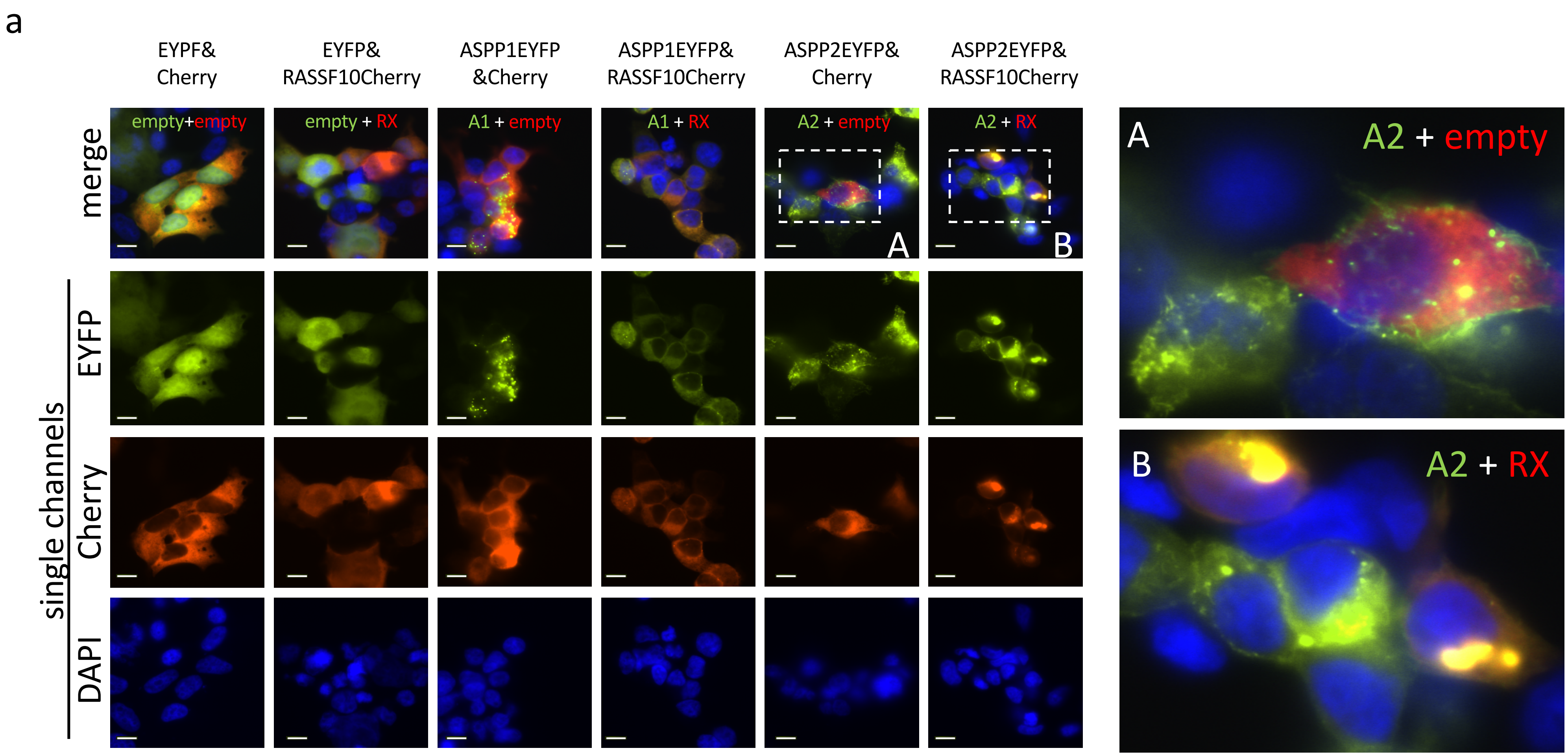

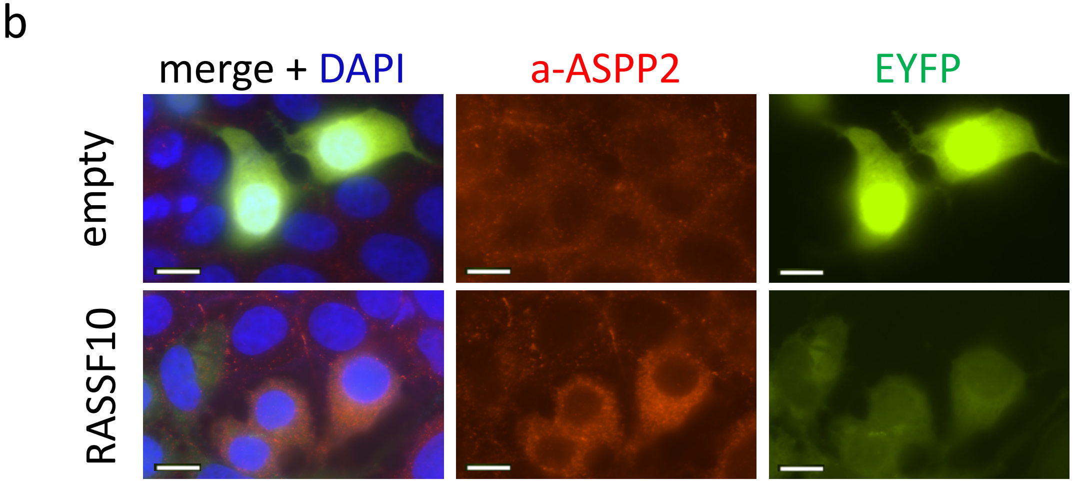

Supplementary Fig. 2: RASSF10 (RX) localises with overexpressed ASPP1/2 (A1/A2) and induces endogenous ASPP2. (a) HEK cells were seeded in 6 well plates on glass slides and transfected the following day with combinations of the following vectors: empty-EYFP, empty-Cherry, RASSF10-Cherry, ASPP1-EYFP and ASPP2-EYFP. 24 h after transfection cells were fixed with formaldehyde, stained with DAPI (0.1 g/ml in PBS, Sigma Aldrich) and embedded in Mowiol/DABCO (Sigma Aldrich). **(b)** HEK were transfected with RASSF10-EYFP and empty-EYFP, formaldehyde fixed, 5% BSA 0,2% TritonX PBS blocked, incubated with a-ASPP2 antibody (1:50; 4°C over night; in 1% BSA 0,5%Triton PBS), incubated with a-mouse AlexaFluor568 (1:1000; 1hRT; in PBS), DAPI stained, embedded in Mowiol. Cells were analysed using Axio Observer Z1 (Zeiss) and Volocity Software (Perkin Elmer) (white bar=5 µm standard).

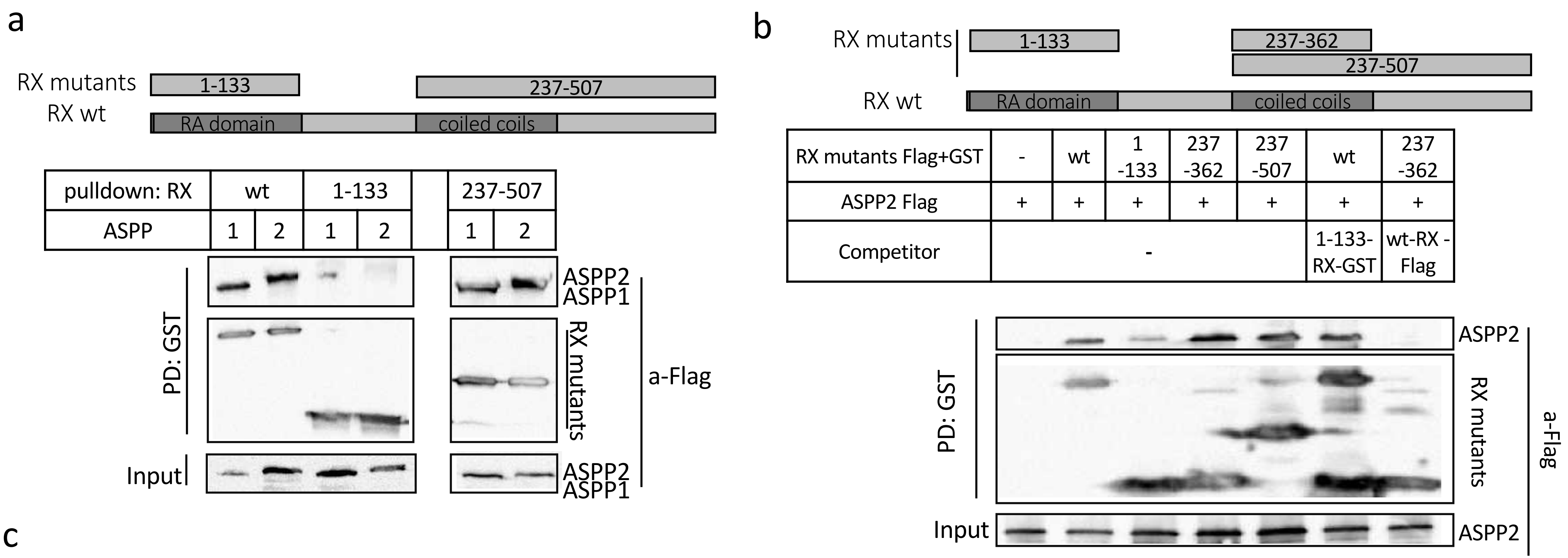

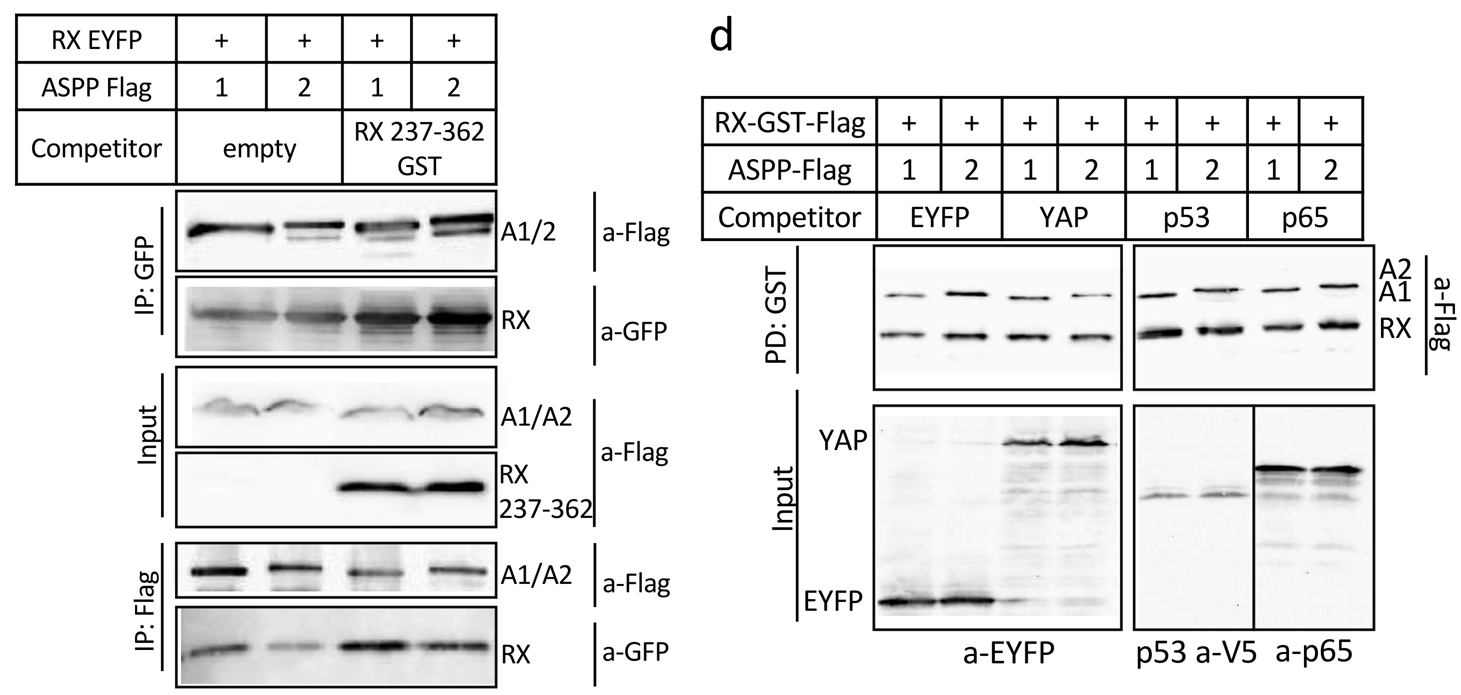

Supplementary Figure S3. Characterization of RASSF10 binding to ASPP1/2 and competition. (a) RASSF10 binds to ASPPs through its C-terminal region. RXwt and RX domain mutants tagged with Flag+GST were overexpressed in HEK cells together with Flag tagged ASPP1 and ASPP2. After 24h pulldown using Glutathione Sepharose precipitated GST-tagged wt-RX and coprecipitated ASPPs. Co-prepitation of ASPPs failed for mutant 1-133, that lacks the coiled coil structures. Protein lysates were separated in SDS-Page and western blotting using anti-Flag antibody detected overexpressed RX and ASPPs. (**b**) RASSF10 coiled coil domain facilitates binding to ASPP2. Rassf10 or its mutants were overexpressed in HEK together with ASPP2. Competition experiments were performed to interfere with RX-ASPP2 binding. Pulldowns for GST via Glutathione Sepharose were followed by SDS-Page and western blotting. RXwt binding of ASPP2 could not be blocked with RX-1-133, which contained RA domain only. RX mutant 237-362 (coiled coils) lost its binding to ASPP2 when RXwt was present. (**c**) Vice versa experiment with RX-EYFP binding ASPPs could not be interfered with by presence of shortened RX (237-326). (**d**) ASPP bind to RASSF10 with higher affinity than to other known ASPP targets. RASSF10, ASPPs and known ASPP binding partners YAP, p53, p65/RelA were co-overexpressed in HEK293. Pulldown for RASSF10 through its GST tag (using Glutathione Sepharose) coprecipitated ASPPs. Presence of YAP, p53 or p65 did not interfere with RASSF10-ASPP binding.

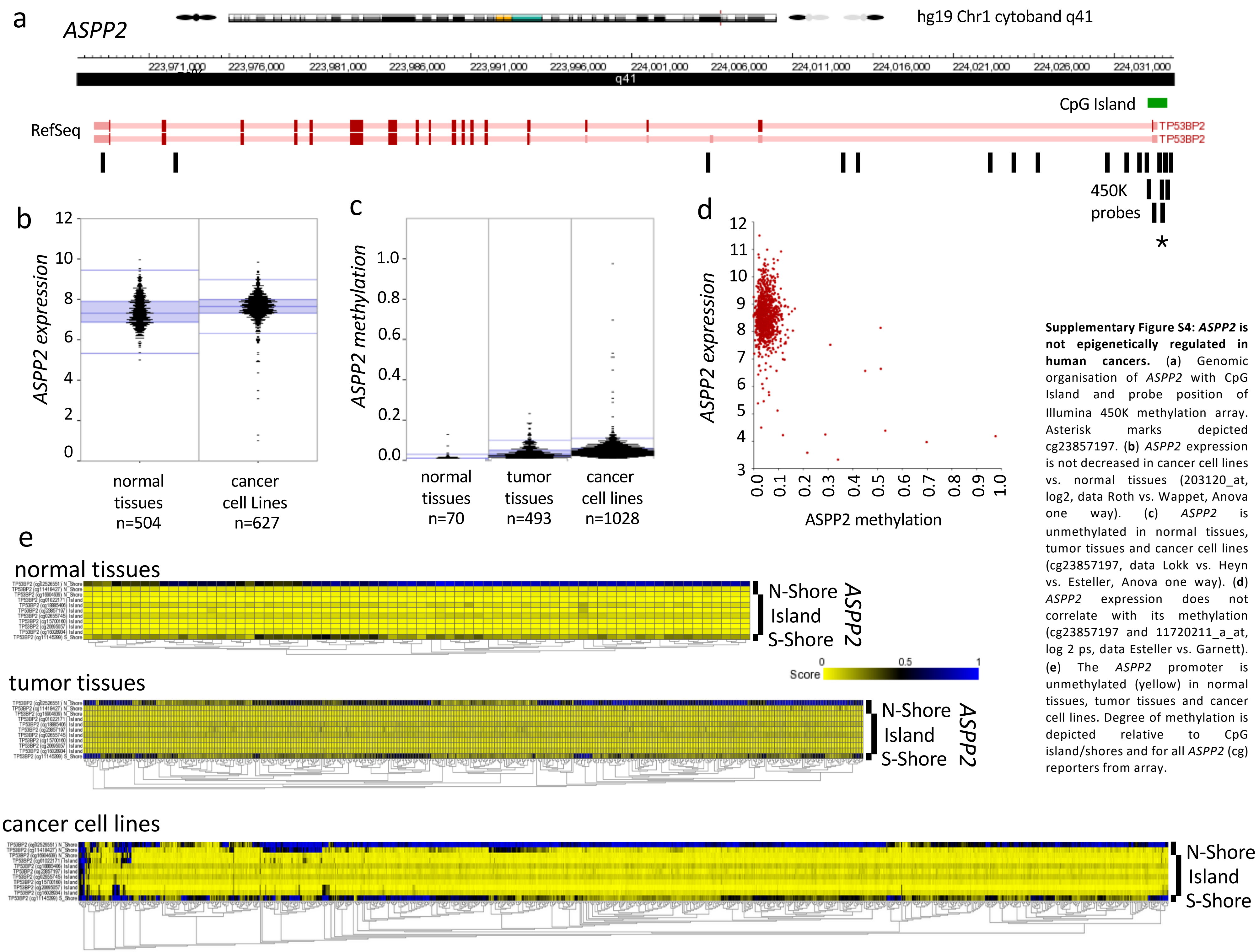

Supplementary Figure S4: *ASPP2* is not epigenetically regulated in human cancers. (**a**) Genomic organisation of *ASPP2* with CpG Island and probe position of Illumina 450K methylation array. Asterisk marks depicted cg23857197. (**b**) *ASPP2* expression is not decreased in cancer cell lines vs. normal tissues (203120_at, log2, data Roth vs. Wappet, Anova one way). (**c**) *ASPP2* is unmethylated in normal tissues, tumor tissues and cancer cell lines (cg23857197, data Lokk vs. Heyn vs. Esteller, Anova one way). (**d**) *ASPP2* expression does not correlate with its methylation (cg23857197 and 11720211_a_at, log 2 ps, data Esteller vs. Garnett). (**e**) The *ASPP2* promoter is unmethylated (yellow) in normal tissues, tumor tissues and cancer cell lines. Degree of methylation is depicted relative to CpG island/shores and for all *ASPP2* (cg) reporters from array.

GSM1246721: Smad3 ChIP-seq, A549 cells:

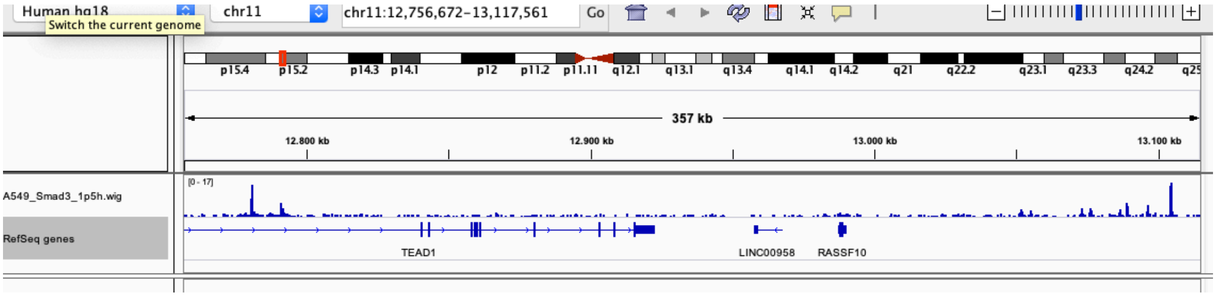

GSM1246713: Smad3 ChIP-seq, H441/siControl cells:

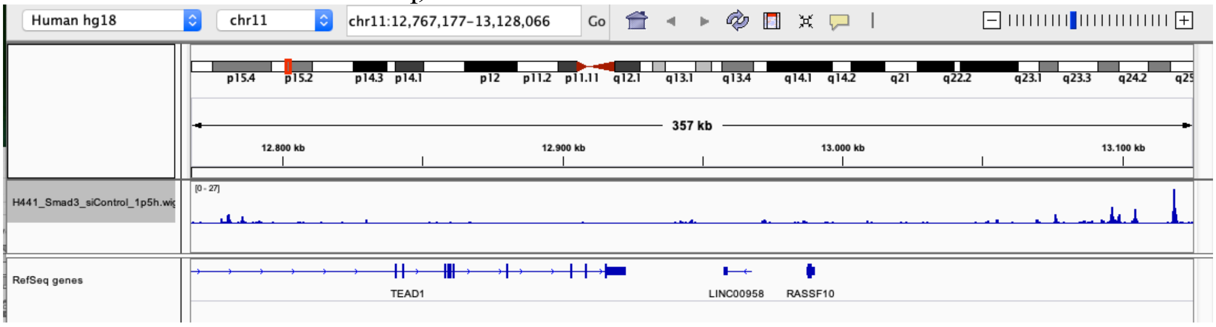

GSM1558744: Smad2 and Smad3 binding sites in H345-TbRII cells:

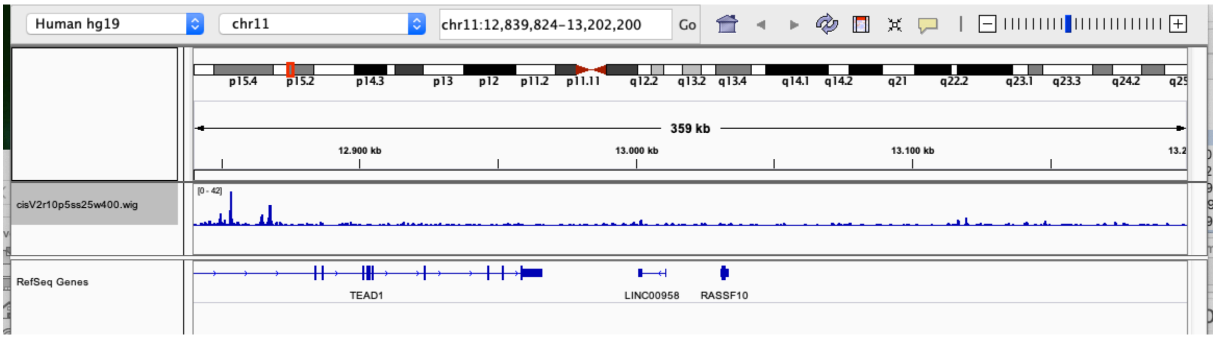

Supplementary Figure S5: No SMAD2 and SMAD3 binding at the RASSF10 promoter. The binding of SMAD2 and SMAD3 were analyzed in different public available Chip-seq data sets by Integrative Genomic Viewer (IGV) in a 400 kb region distal to the RASSF10 gene in A549 and H441 cells (GSE51509: Isogaya K, Koinuma D, Tsutsumi S, Saito RA et al. A Smad3 and TTF-1/NKX2-1 complex regulates Smad4-independent gene expression. Cell Res 2014 Aug;24(8):994-1008) and in H345-TbRII cells (GSE63871: Murai F, Ehata S, Koinuma D, Miyazono K).

Table S1: RASSF10 negatively and positively associated genes that are under- and overrepresented in the gene set hallmark EMT.

RASSF10 negative associated-gene Overrepresented (p=4.7e-07)	RASSF10 positively associated-gene Underrepresented (p=0.03)
ABI3BP, ACTA2, ADAM12, ANPEP, BGN, CALU, CDH11, COL11A1, COL1A2, COL3A1, COL5A1, COL5A2, COL5A3, COL6A2, COL6A3, DAB2, DCN, EFEMP2, ELN, EMP3, FAP, FBLN2, FBLN5, FBN1, FGF2, FLNA, GADD45B, GAS1, GLIPR1, GPX7, ITGA5, ITGB3, LGALS1, LOXL2, LRRC15, LUM, MGP, MMP2, MMP3, NID2, NOTCH2, NTM, P3H1, PCOLCE, PDGFRB, PLOD1, PMP22, POSTN, PRRX1, PTX3, RGS4, SGCD, SGCG, SPARC, TGFB1, THBS2, THY1, TPM4, VIM, WIPF1, WNT5A	AREG, CAPG, CXCL1, CXCL6, CXCL8, DKK1, FBLN1, FUCA1, GPC1, IGFBP2, IGFBP3, ITGA2, ITGAV, ITGB5, JUN, LAMA3, LAMC2, MATN2, MEST, NT5E, PFN2, PLOD2, PMEPA1, PRSS2, PTHLH, PVR, RHOB, SAT1, SDC1, SDC4, TGFBI, THBS1, TNFRSF12A, TPM1

Supplementary Table S2. 5-year cancer patient survival *RASSF10* expression dependent

Cancer		5-year survival probability		patient cohort (n)		
**type**	**subtype**	**RX low expression**	**RX high expression**	**RX low expression**	**RX high expression**	**p value**
kidney cancer	papillary	60%	84%	111	176	1.7e-03
	clear cell	59%	71%	322	208	2.0e-02
head&neck cancer	squamous cell	33%	51%	152	347	1.0e-04
lymphoma	mantle cell	26%	48%	72	49	1.7e-02
	B-cell	5%	10%	41	124	5.6e-02
thymoma		88%	96%	36	82	6.0e-02
liver cancer		28%	62%	92	272	8.3e-06
lung cancer		52%	70%	806	339	5.5e-09
gastric cancer		47%	54%	390	241	6.9e-02
breast cancer		56%	77%	97	155	1.7e-03

Supplementary Table S3. Primer list

	gene		Sequence (upper and lower primer)
**RT-Primers**	*ß-ACTIN*		CCTTCCTTCCTGGGCATGGAGTC
			CGGAGTACTTGCGCTCAGGAGGA
	GAPDH		TGGAGAAGGCTGGGGCTCAT
			GACCTTGGCCAGGGGTGCTA
	RASSF10		GCGCCATGGATCCTTCGGAAAA
			GGCAGCGCCTCGTCGTCGTCCT
	ASPP2		AAGATGATGCCGATGTTTCTTACC
			CGCAACTGGACGTTCTTTTT
	SNAI2		CATTCGGGAGAAGGTCCGAG
			CATTCGGGAGAAGGTCCGAG
	SPOCK1		CAGCCTGTCCACACAAAAGC
			TGGTCAATTTGGGATGGGGG
	MMP2		GTCTGTGTTGTCCAGAGGCA
			ATCACTAGGCCAGCTGGTTG
	COL5A1		CTTGGCCCAAAGAAAACCCG
			GTAGGTGACGTTCTGGTGGG
	CDH1		GTCACTGACACCAACGATAATCCT
			TTTCAGTGTGGTGATTACGACGTTA
	CTGF		CATCTTCGGTGGTACGGTGT
			GACCAGGCAGTTGGCTCTAA
**Methylation**	*RASSF10* semi-nested PCR		ATAAGTAGAGGAGTTAGTAGGTTAAAGGAGA
			AAATACAAAAAACTCAAAACCCAAACCC
			GTGGAGGGATTTTTGAATTTTTTTT
	ASPP2 semi-nested PCR		TTYGTTTTTTTTYGTTTGGTTTAG
			CCTCAAAAAAACAAAATAATAATAAATAACA
			GTTTTGTTTTGAAGGTAAAGGGTTTTT

Figure 4b Pulldown GST/a-FLAG

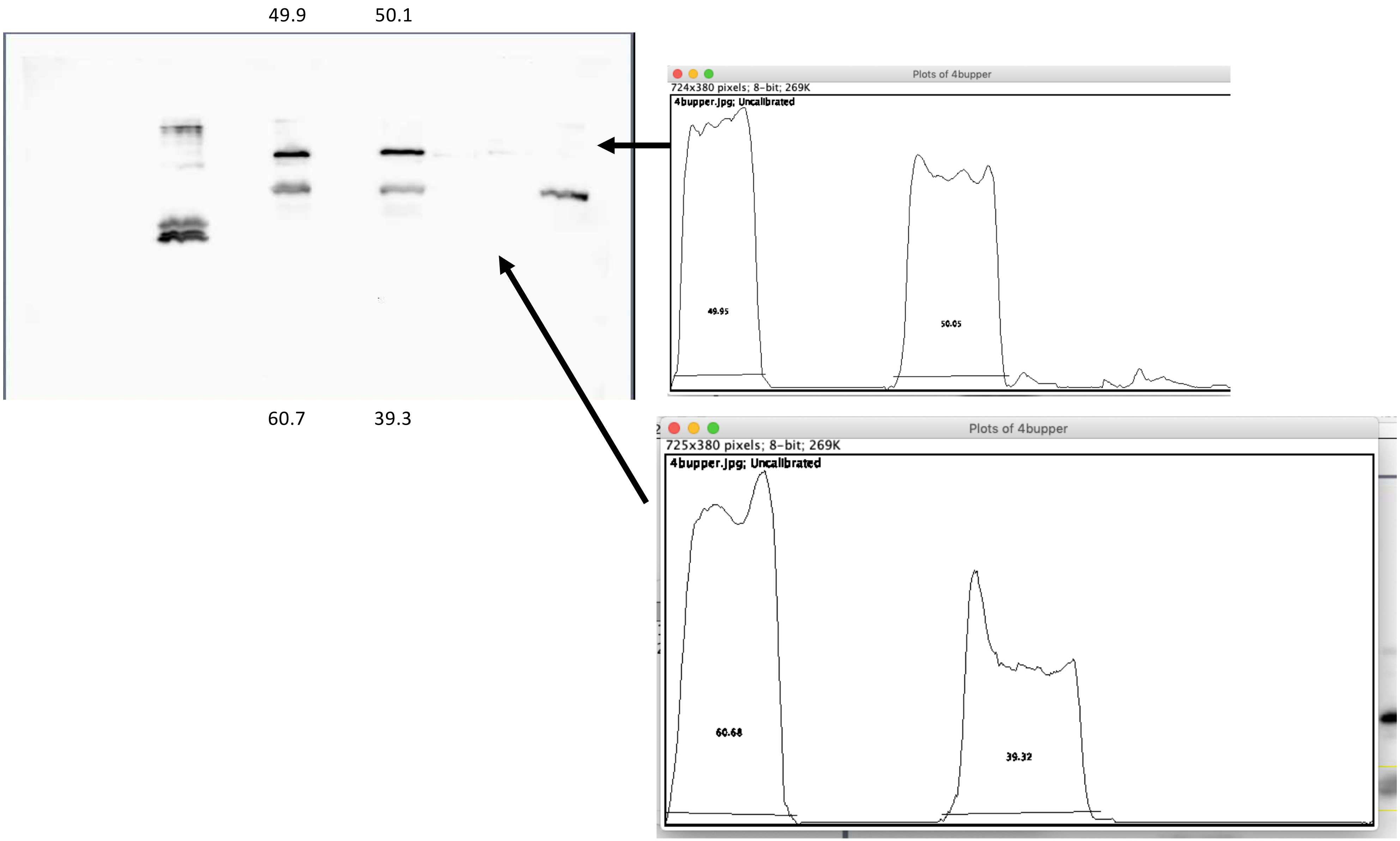

Figure 4b IP/Flag

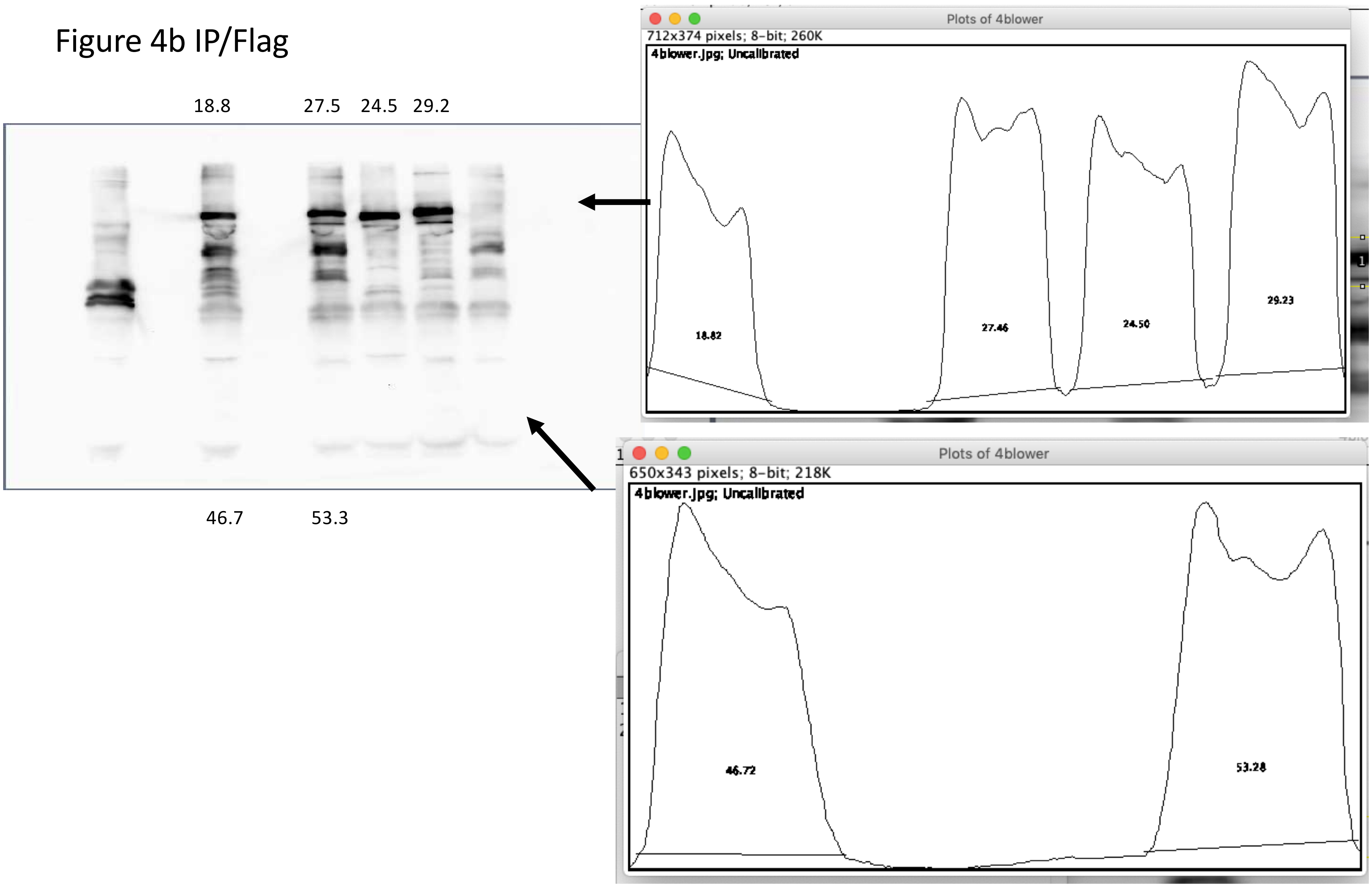

Figure 4c ASSP2

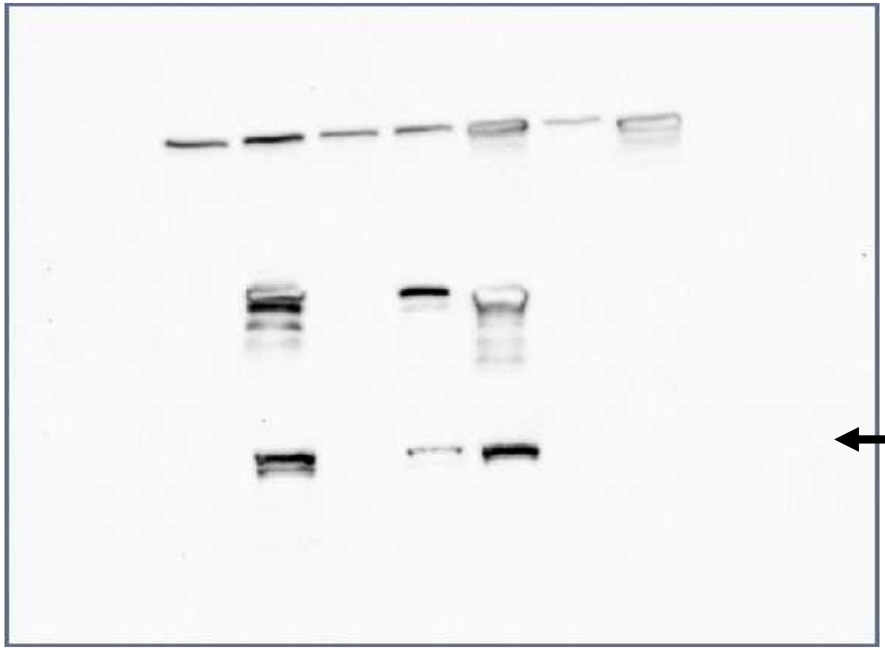

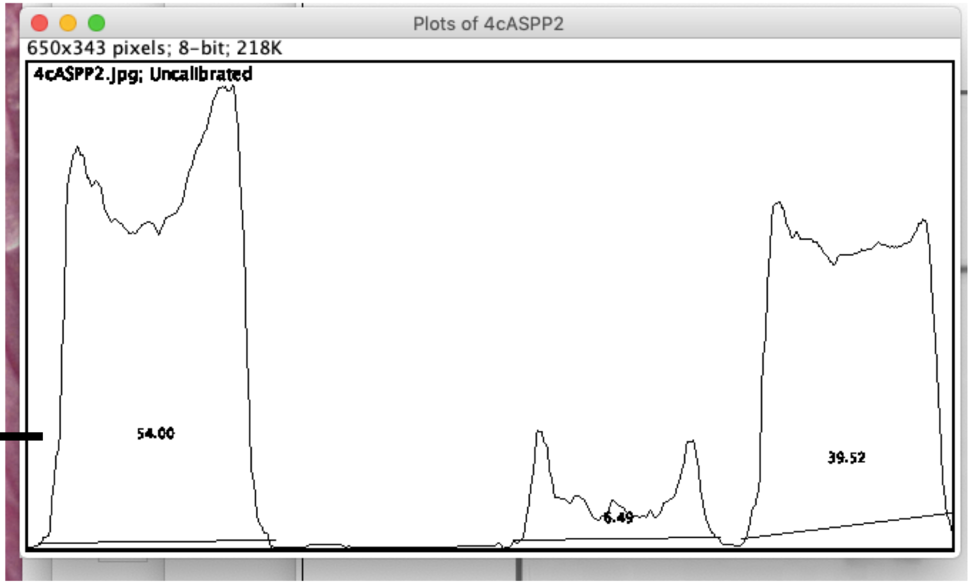

54.0. 6.5 39.5

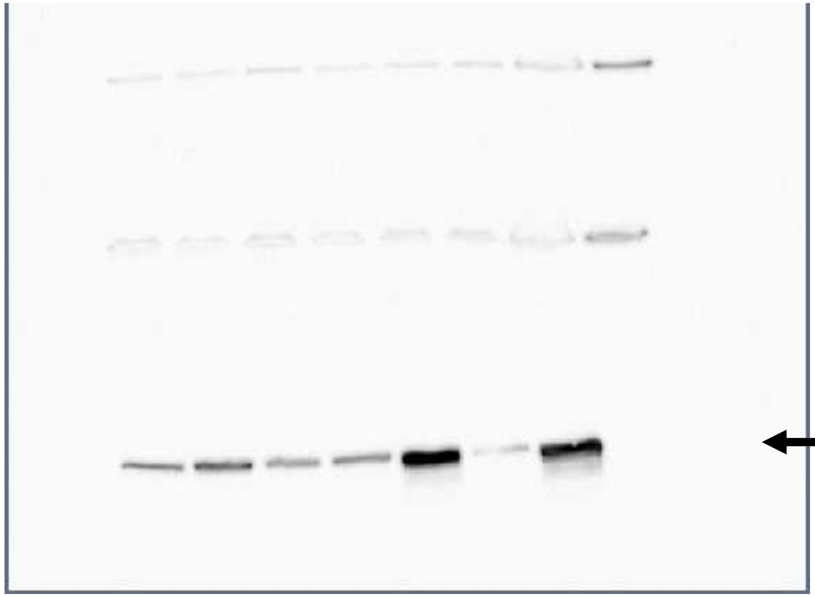

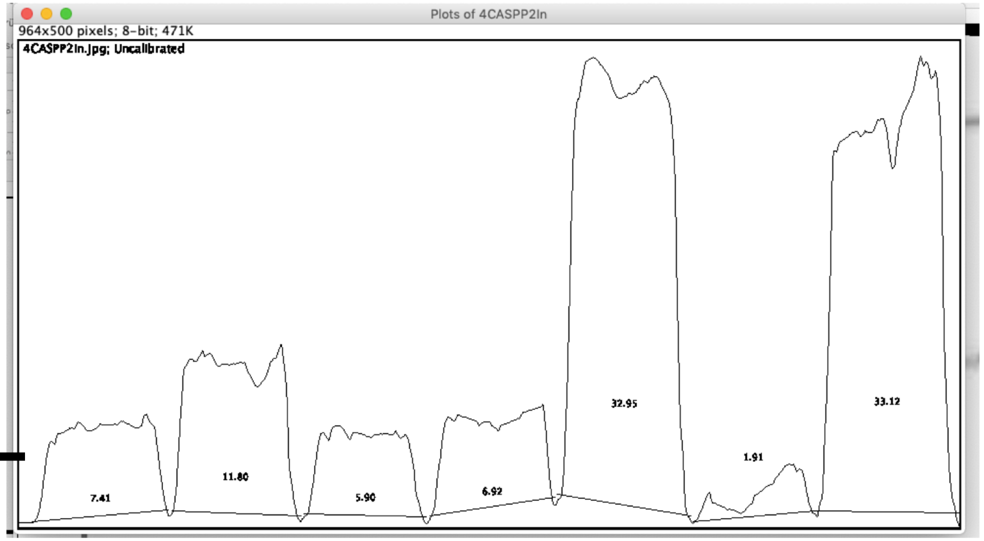

7.4 11.8 5.9 6.9 32.9 1.9 33.1

Figure 4c ASSP1

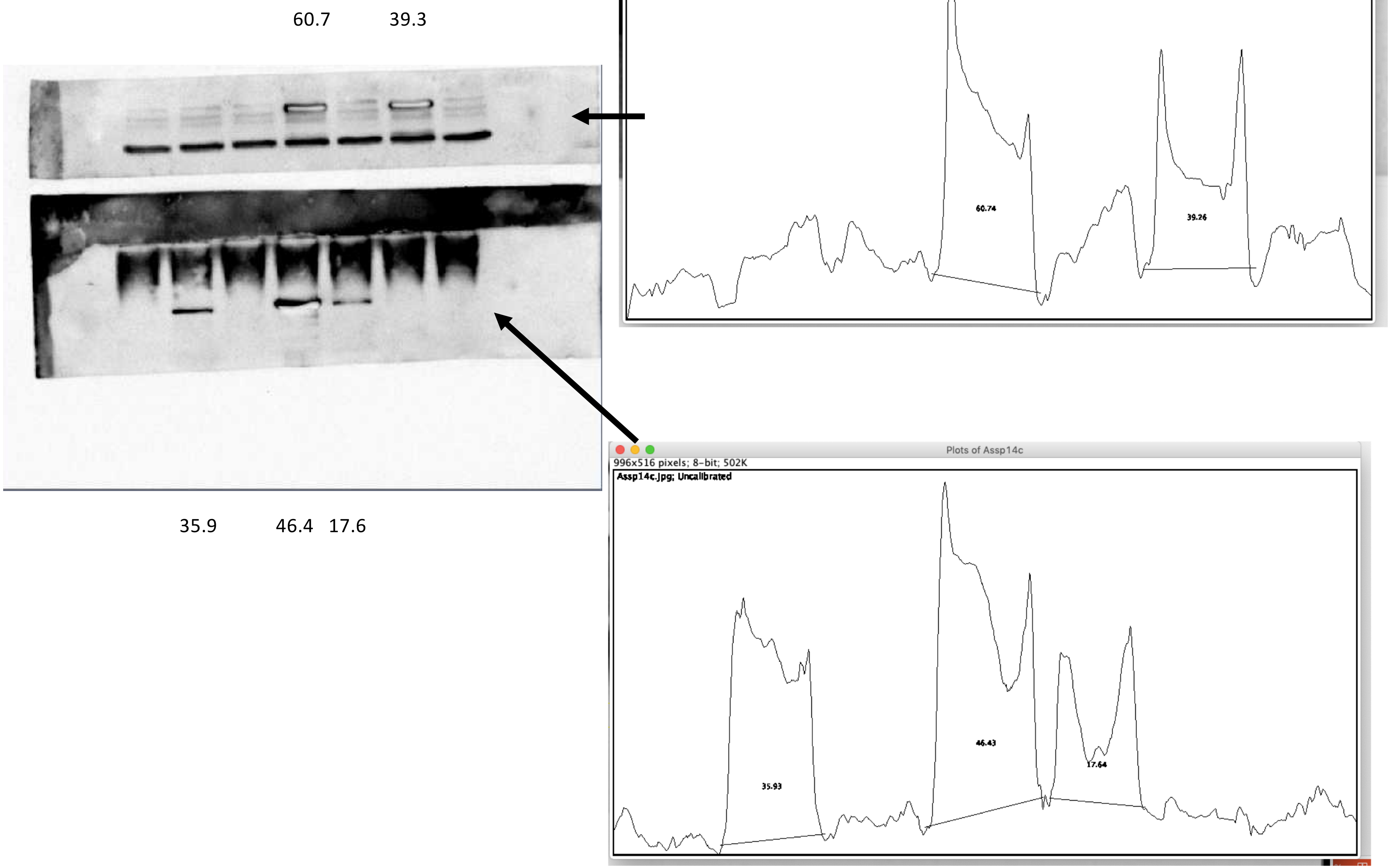

Figure 4c RASSF10 Pulldown

76.5 13.4. 10.7

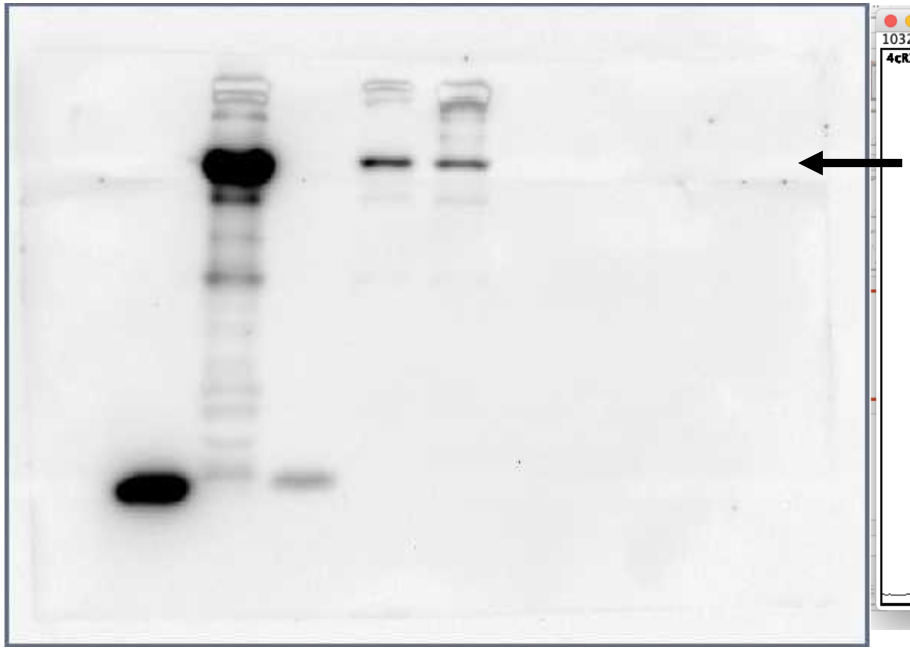

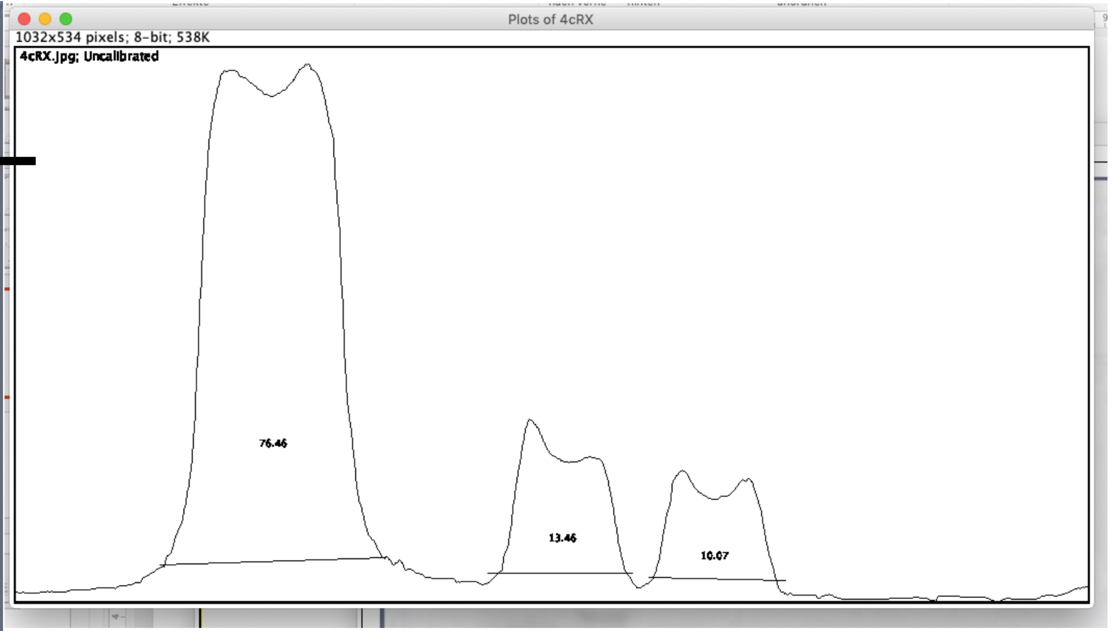

Figure 4c GST Pulldown

Figure 4c GAPDH

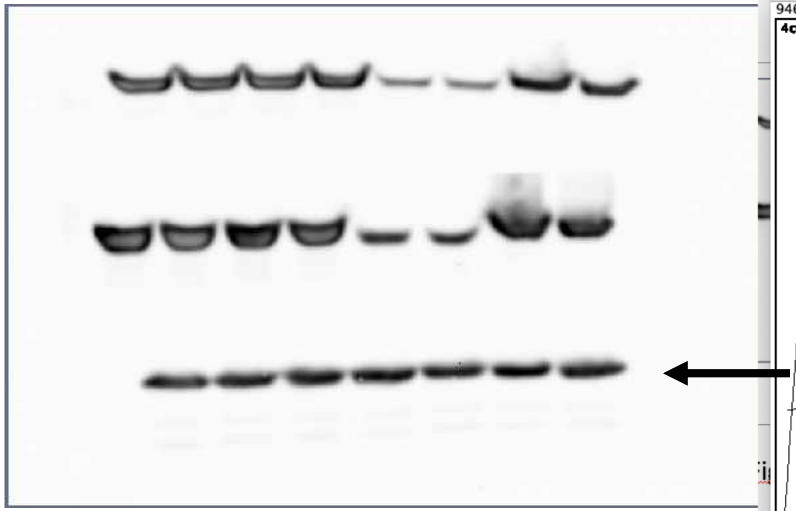

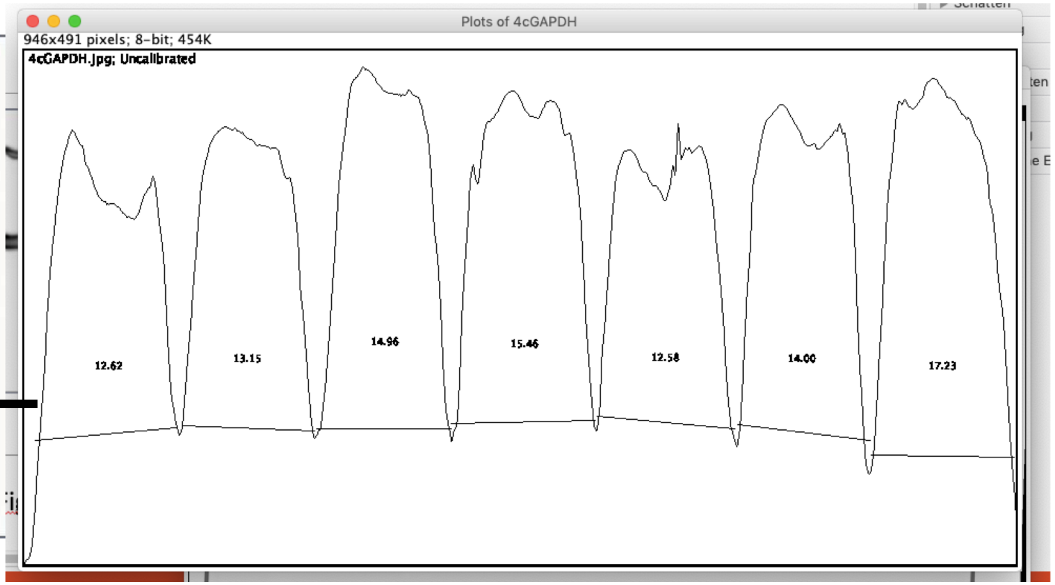

12.6 13.1 15.0 15.5 12.6 14.0 17.2

Figure 4c RASSF10 Input

75.3 12.7. 12.2

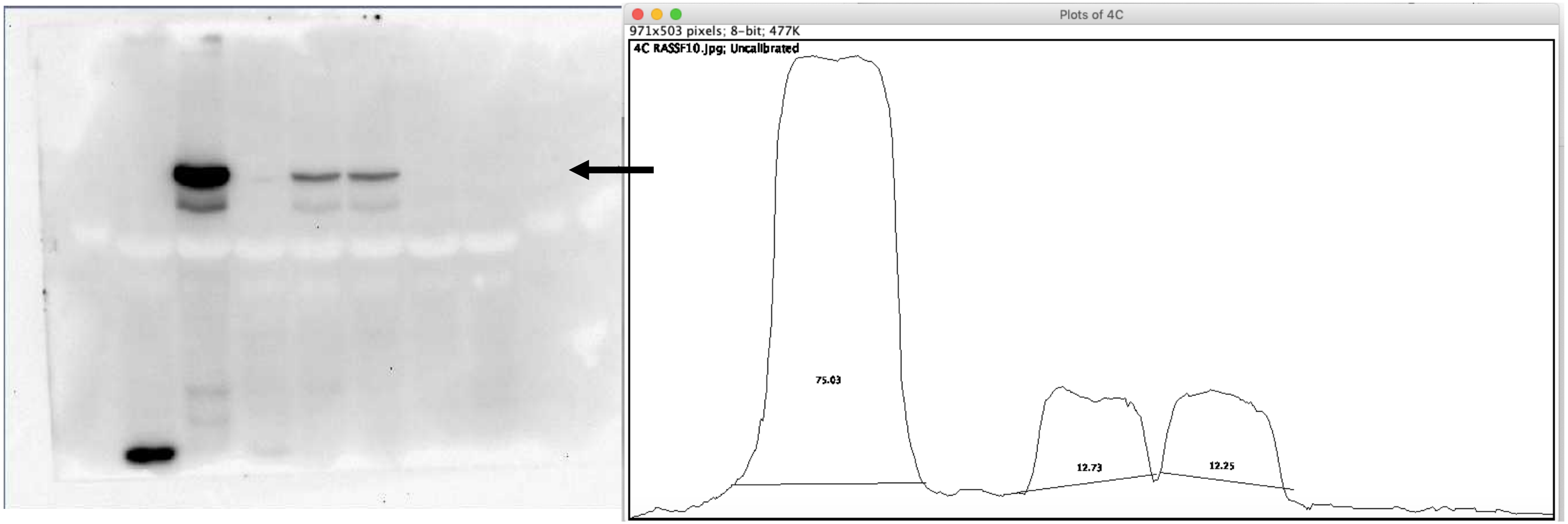

Figure 4c GST Input

Figure 4c ASPP1/2 Input

2,4 4,5 3,4 26.7 22.3 20.3 20.6

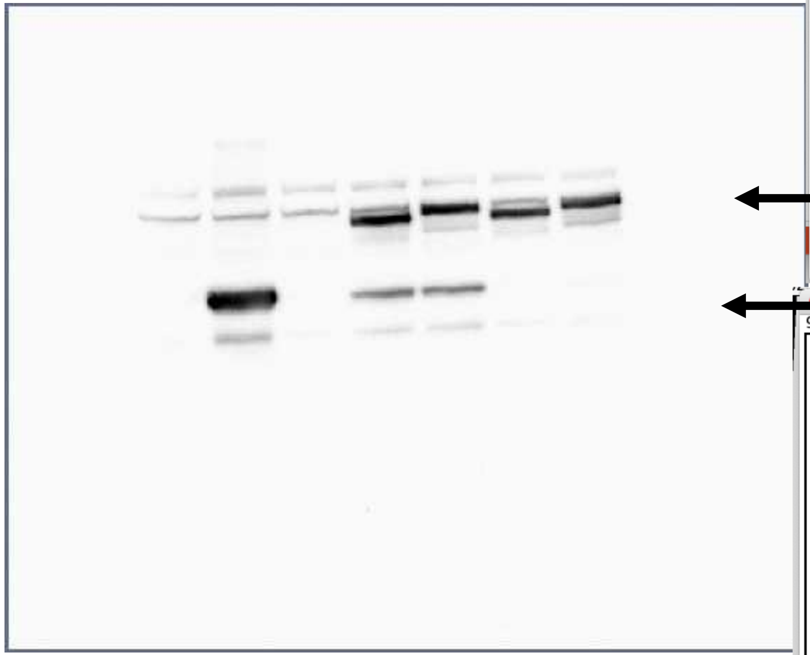

71.8 14.9 13.3

Figure 4c RASSF10 Input

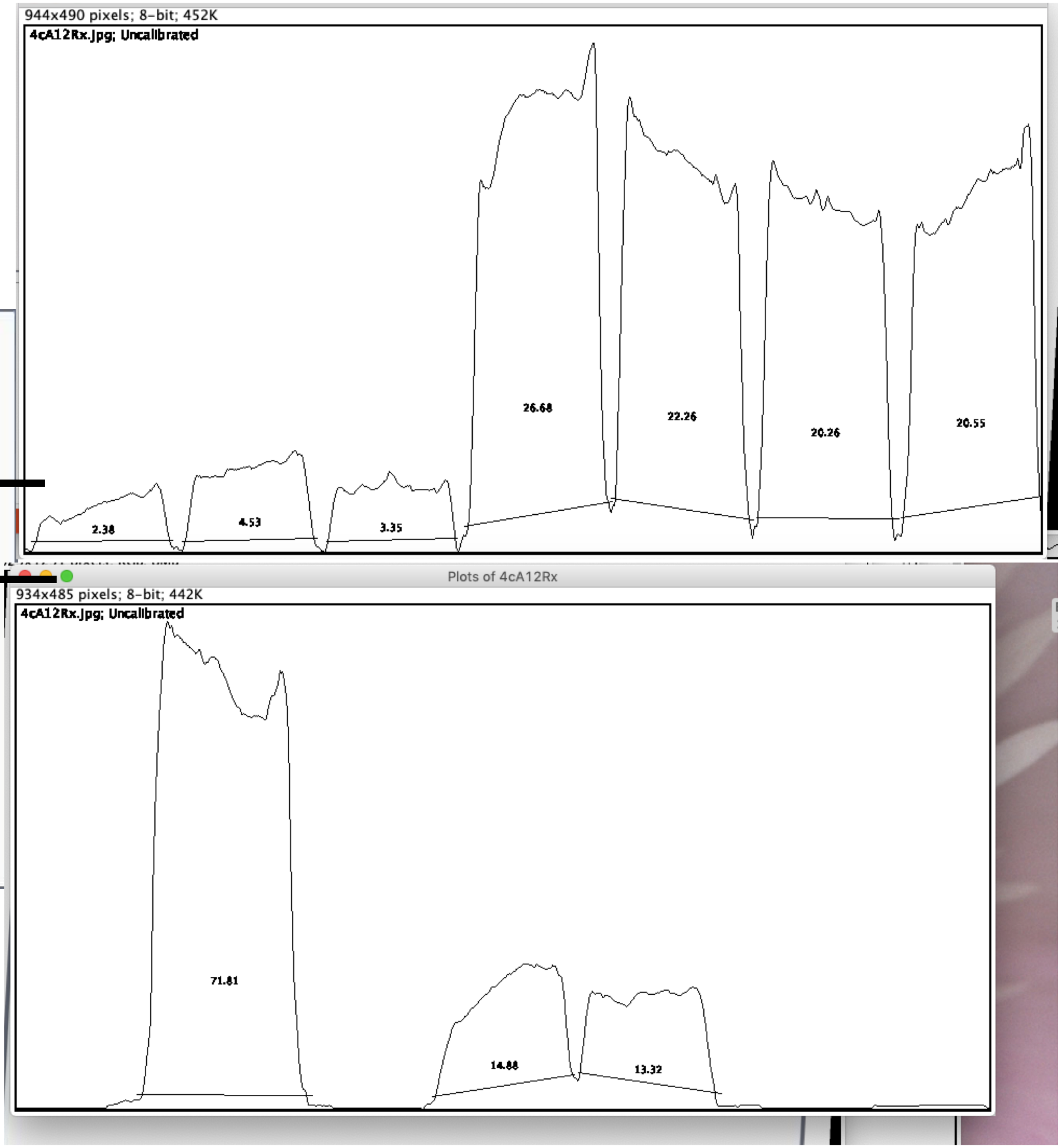

Figure 4d RASSF10

Figure 4d ASPP2/EYFP

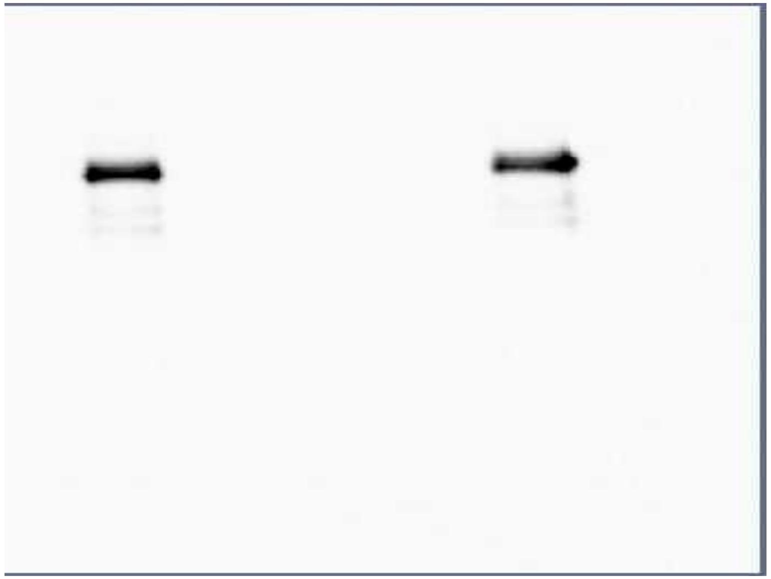

61.0 39.0

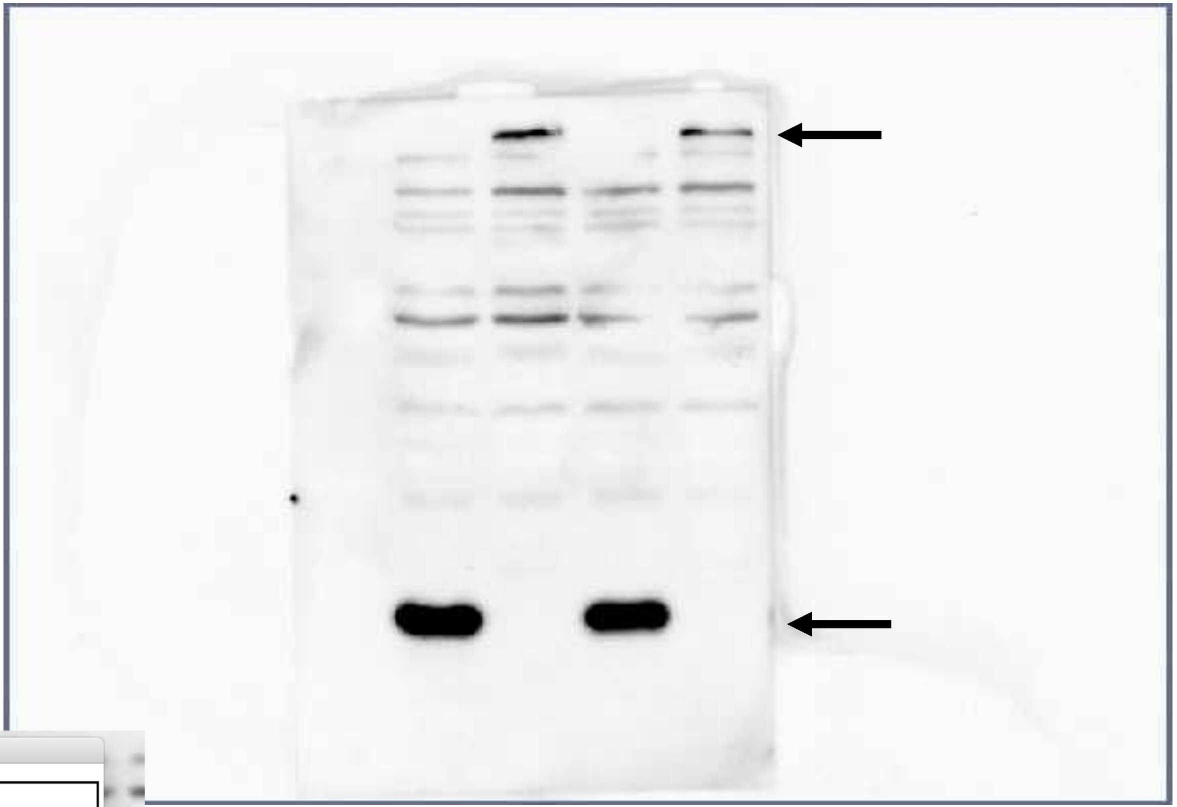

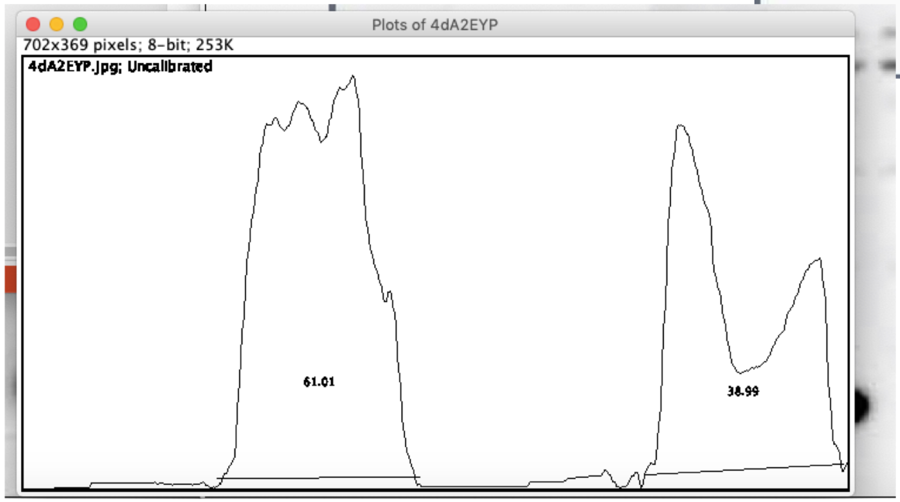

50.5 49.5

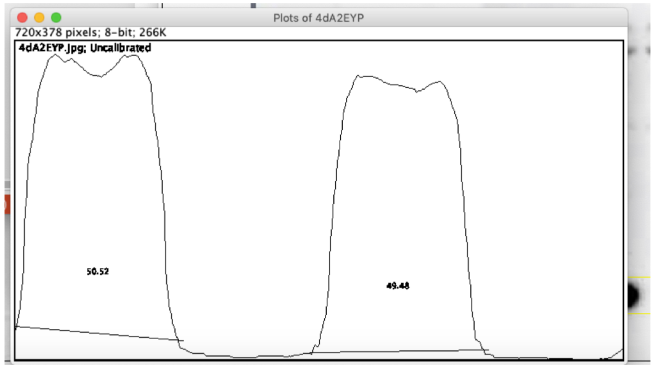

Figure 4d RASSF10 Input

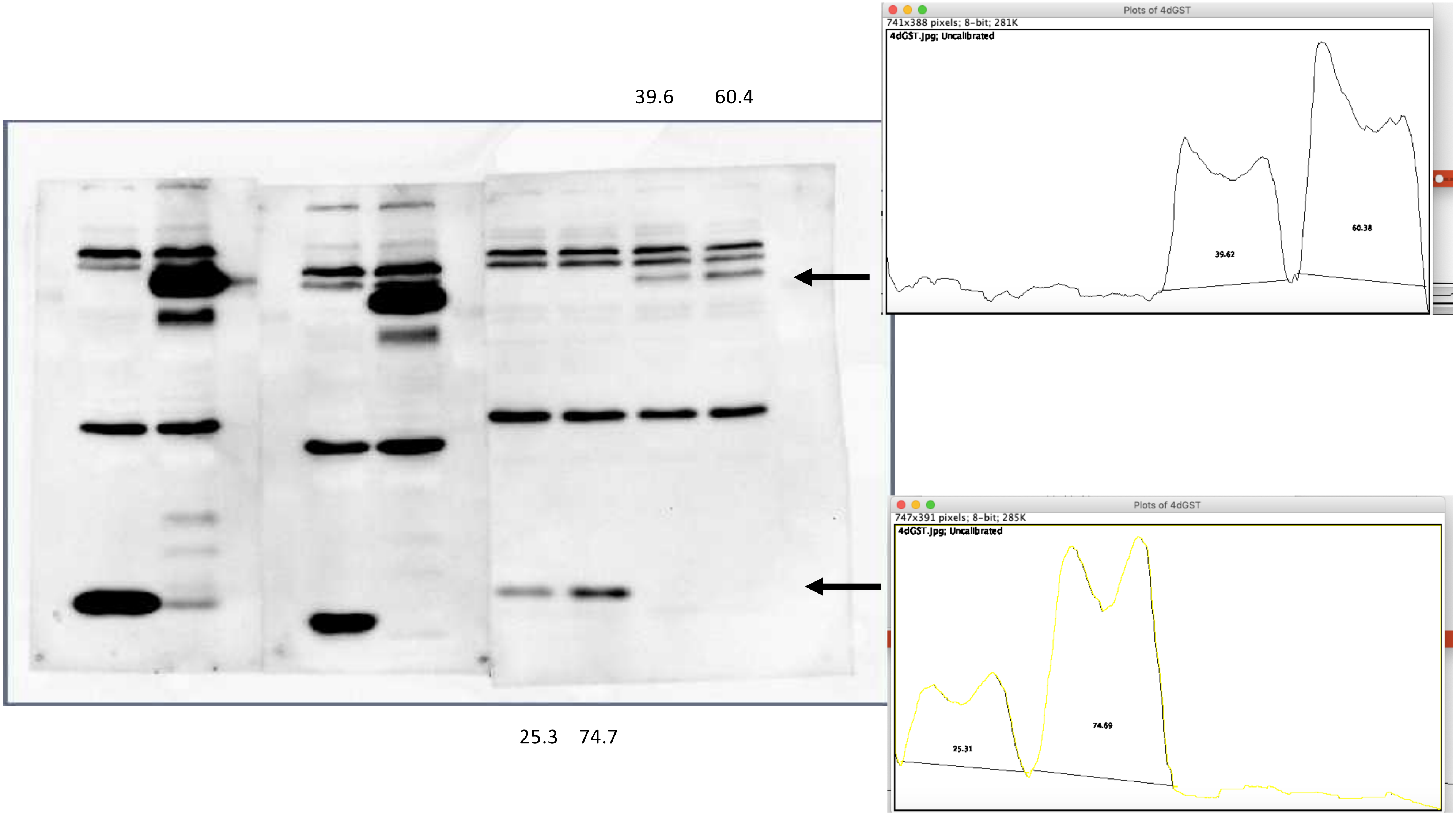

Figure 4d GST

Figure 4d GAPDH

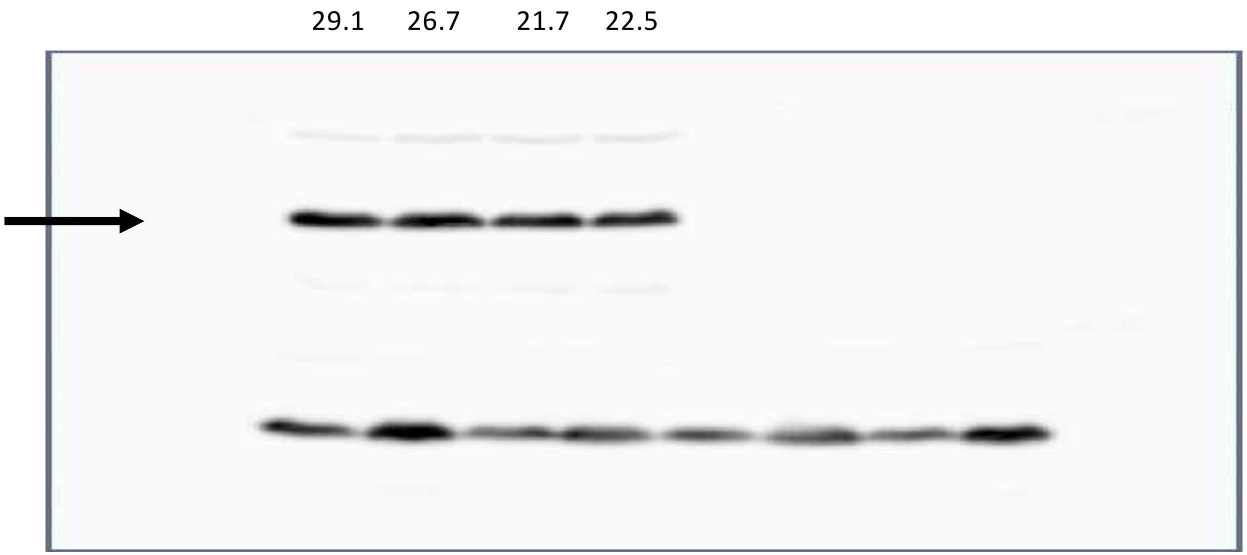

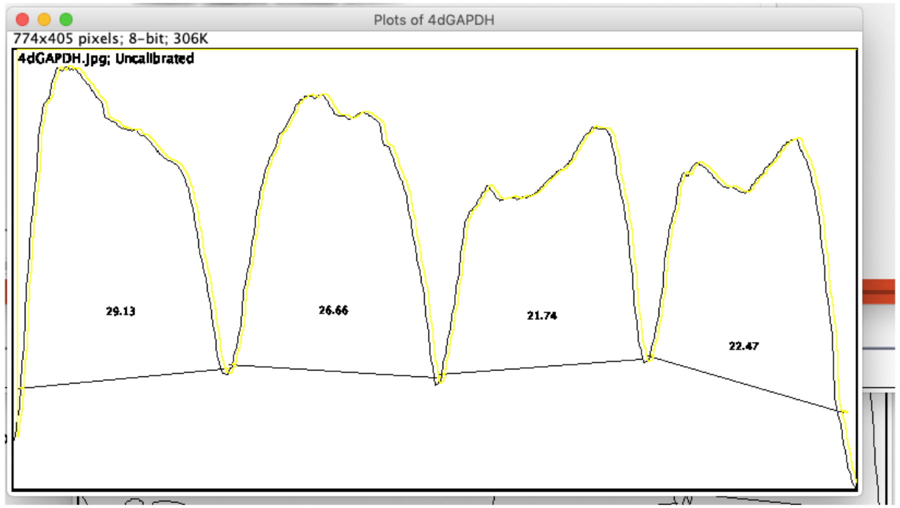

Figure 4e ASPP2

53.3 46.7

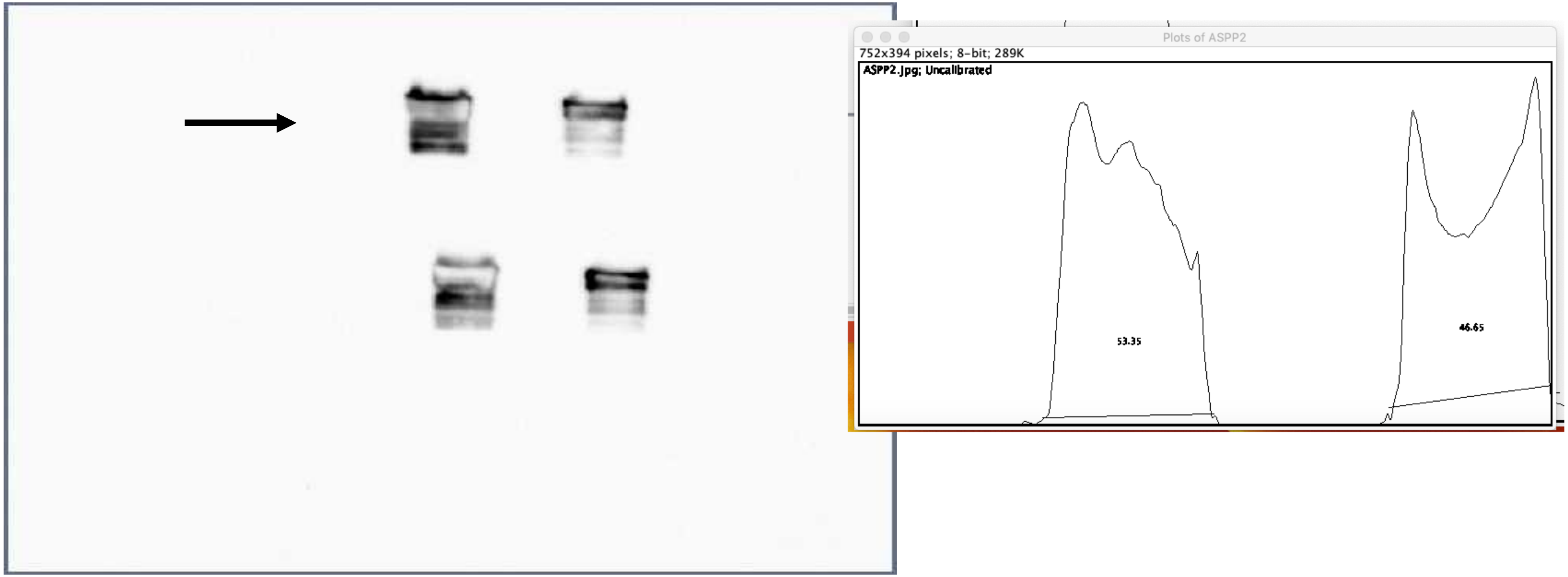

Figure 4e RASSF10

8.7 91.3

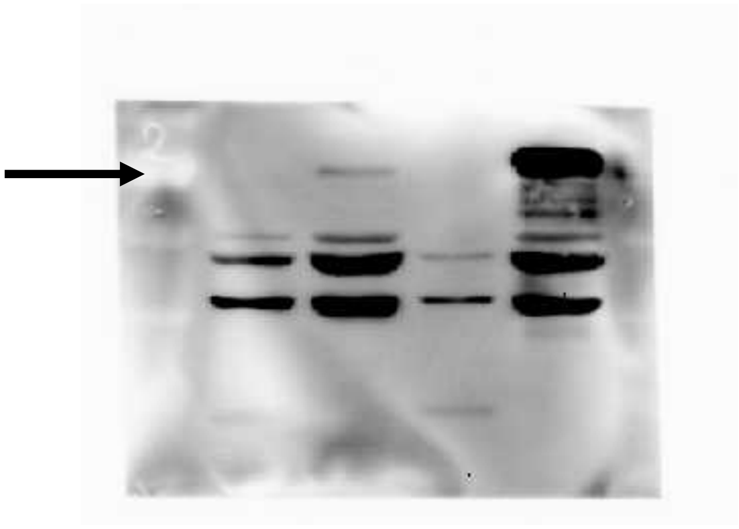

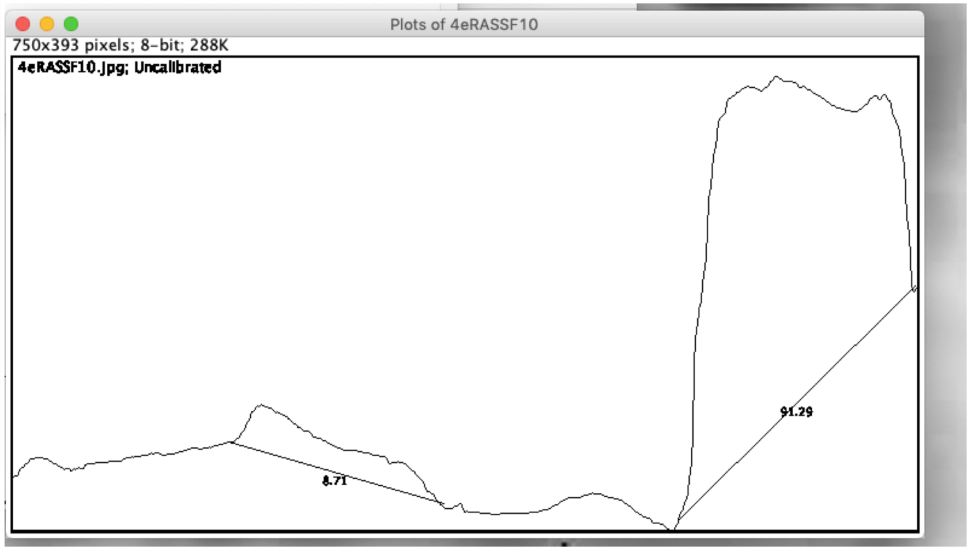

Figure 4e ASPP2

78.2 21.9

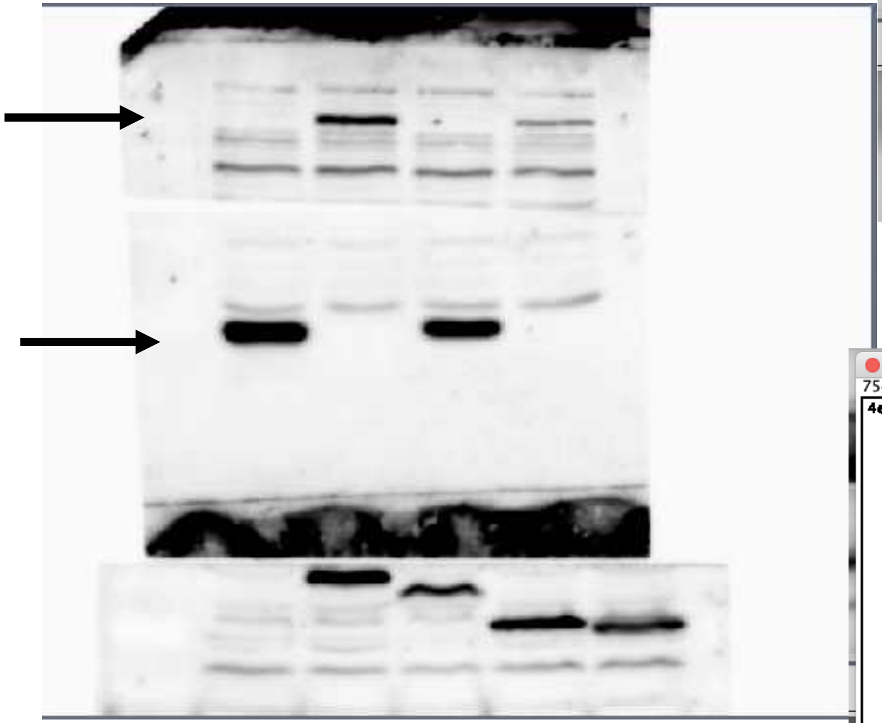

56.5 43.6

Figure 4e EYFP

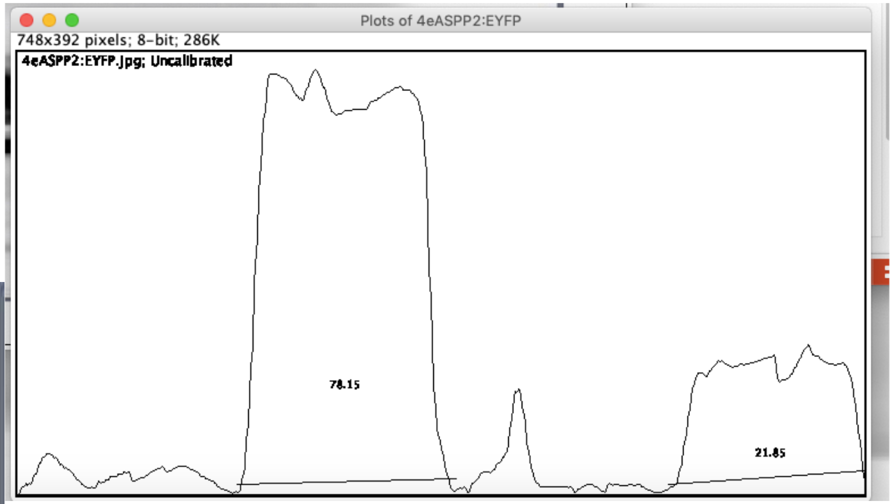

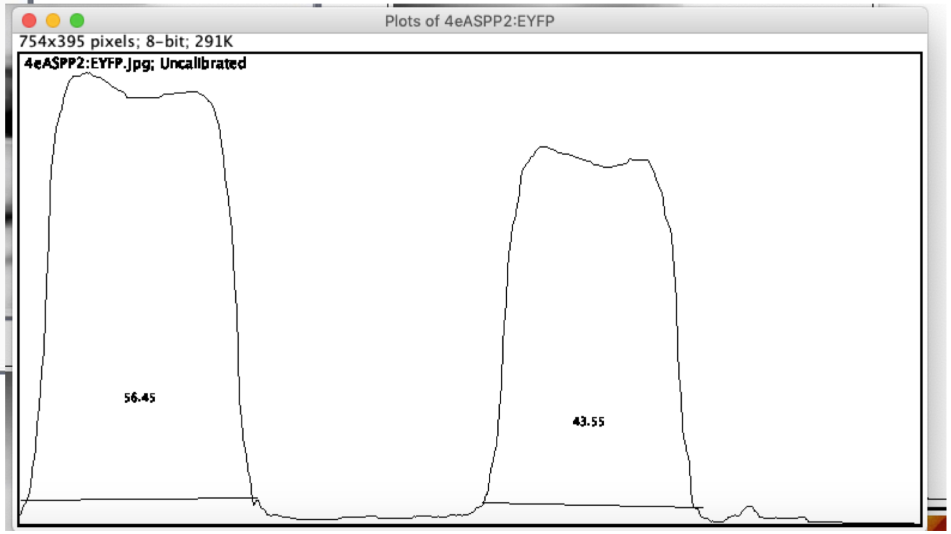

Figure 4e GAPDH

30.4 27.0 21.5 21.0

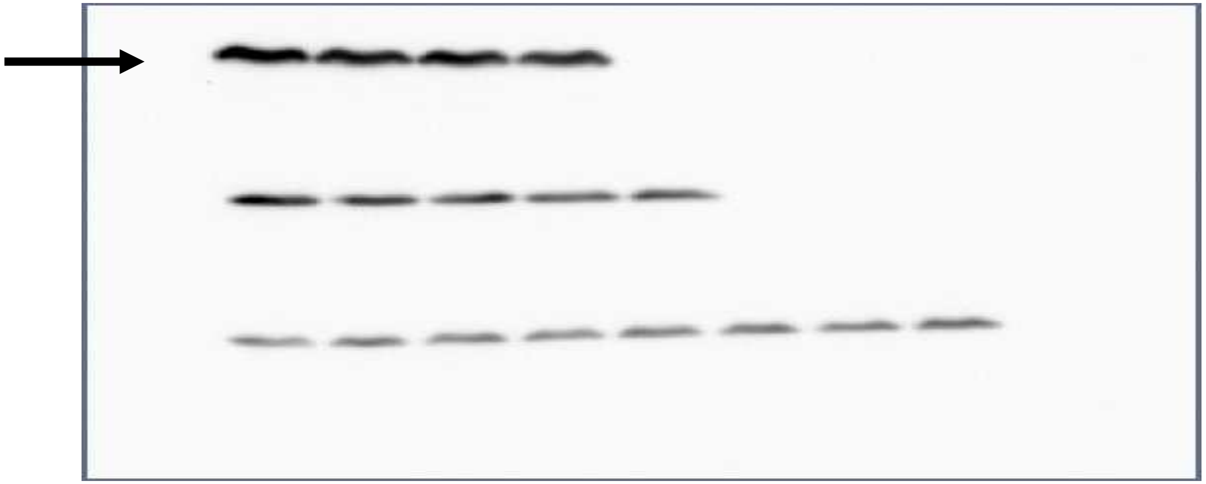

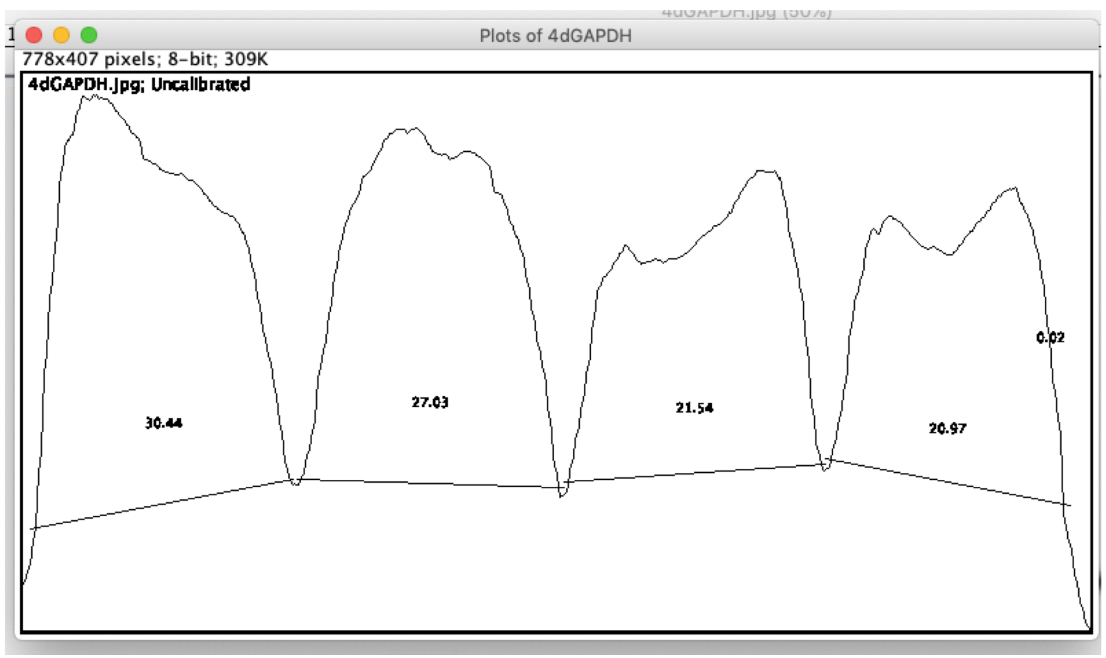

18.2 19.3 16.7 18.0 18.2 9.6

Figure 4f ASPP2

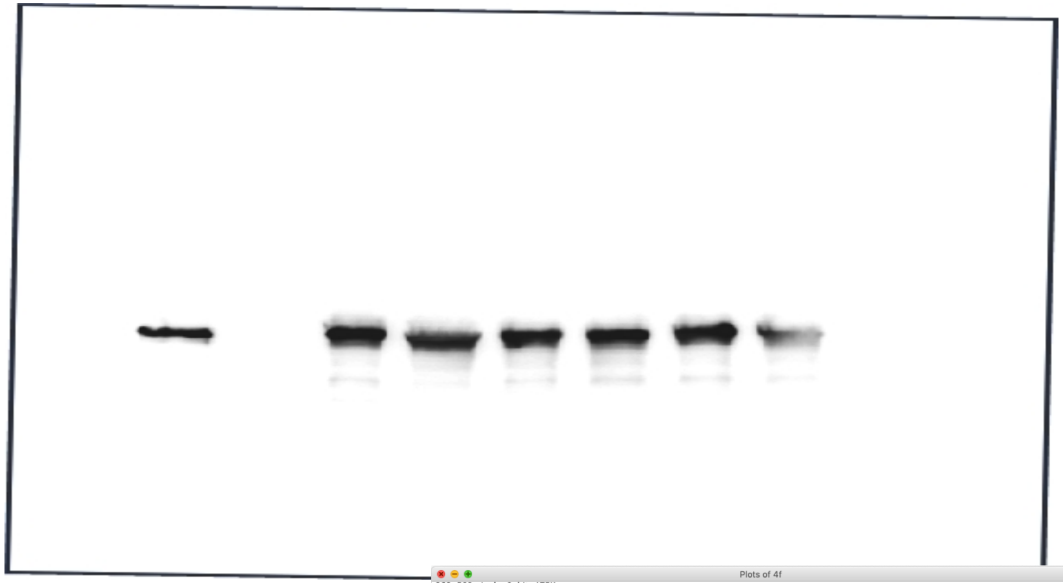

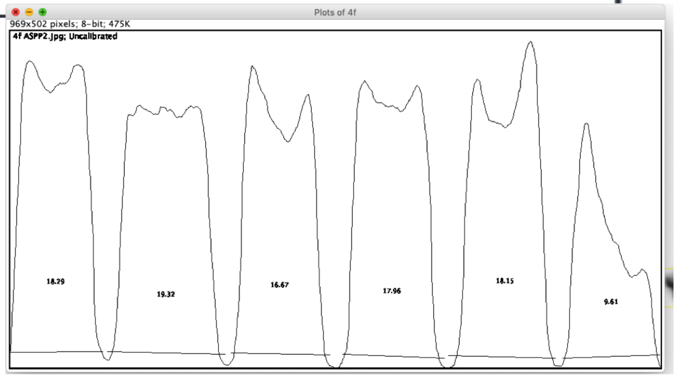

Figure 4f RASSF10

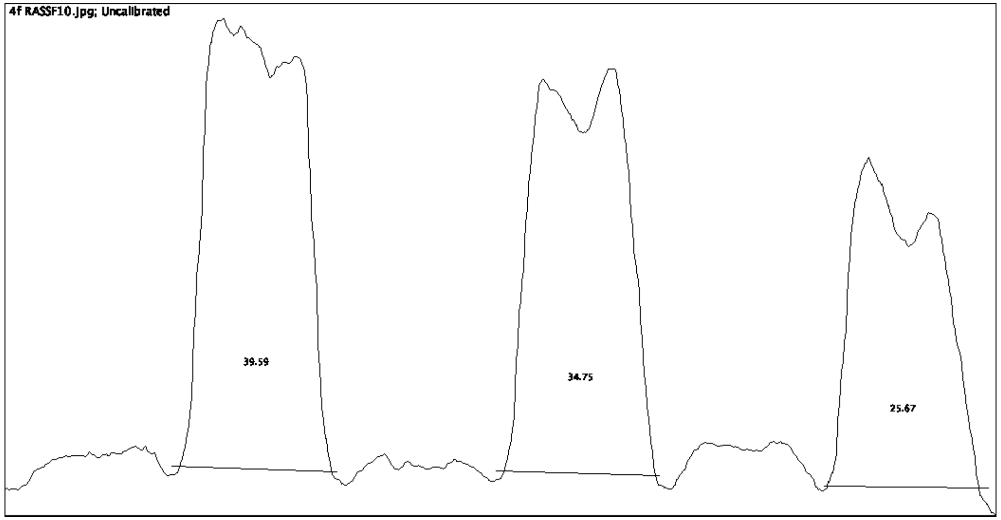

39.6 34.8 25.7

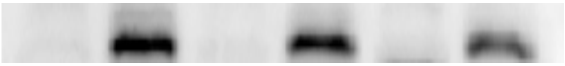

Figure 4f GAPDH

14.3 19.0 13.8 19.2 16.3 17.5

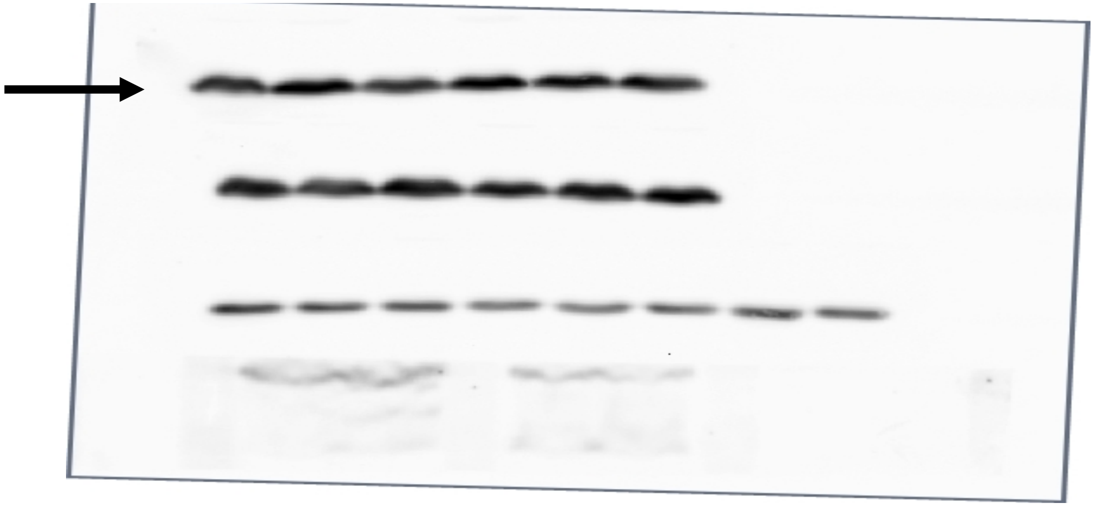

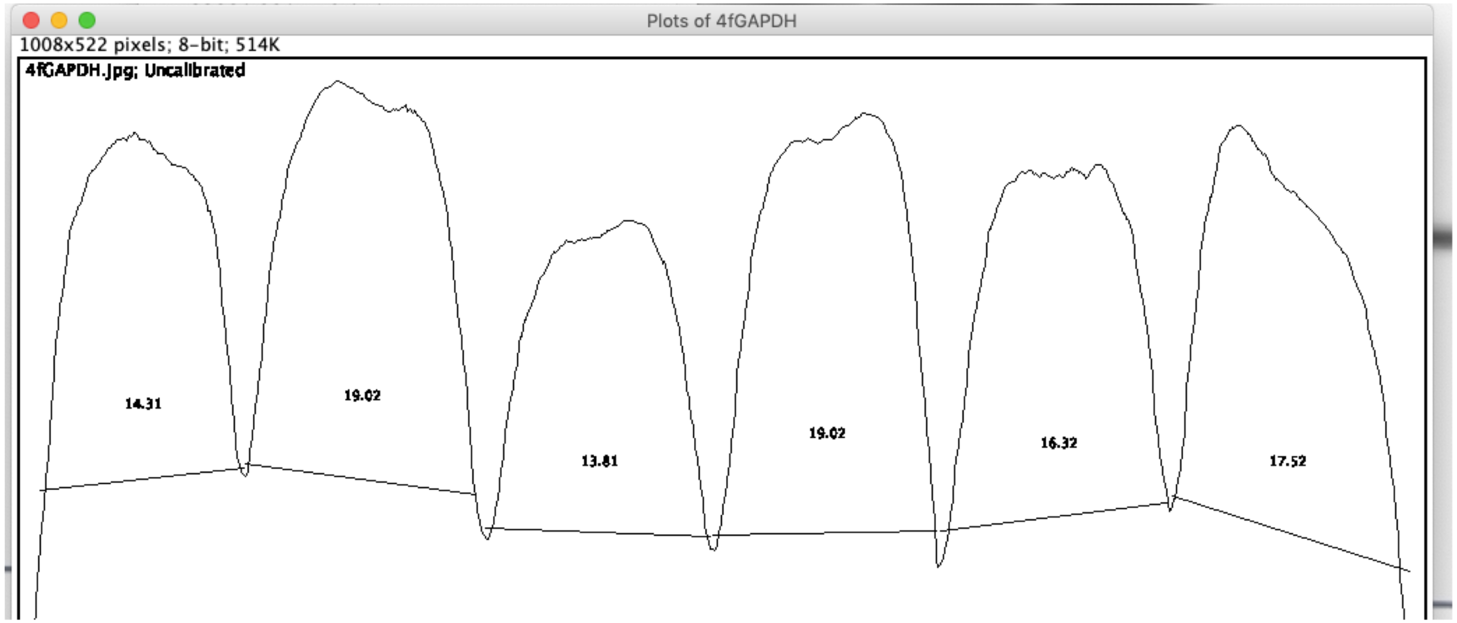

Figure 5a ASPP2/EYFP HEK293

37.9 62.1

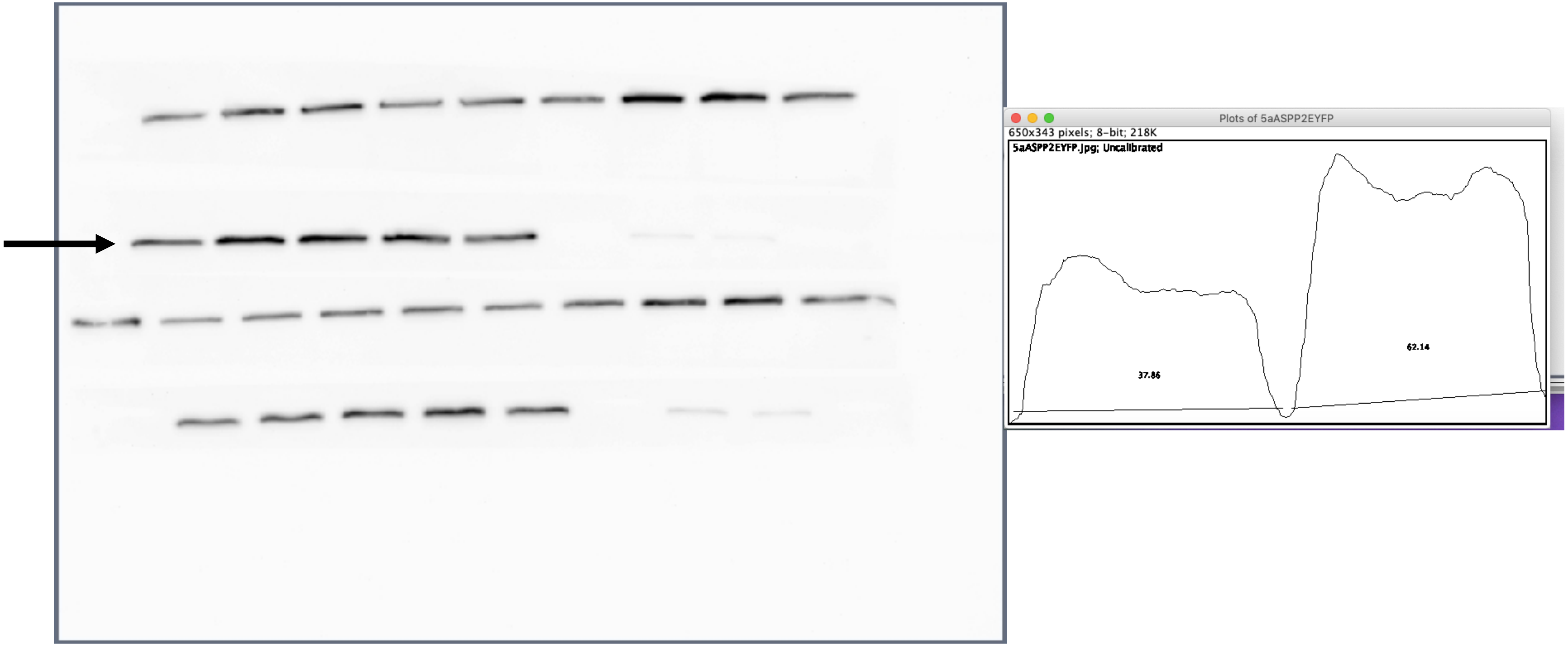

Figure 5a ASPP2/GST HEK293

32.7 67,3

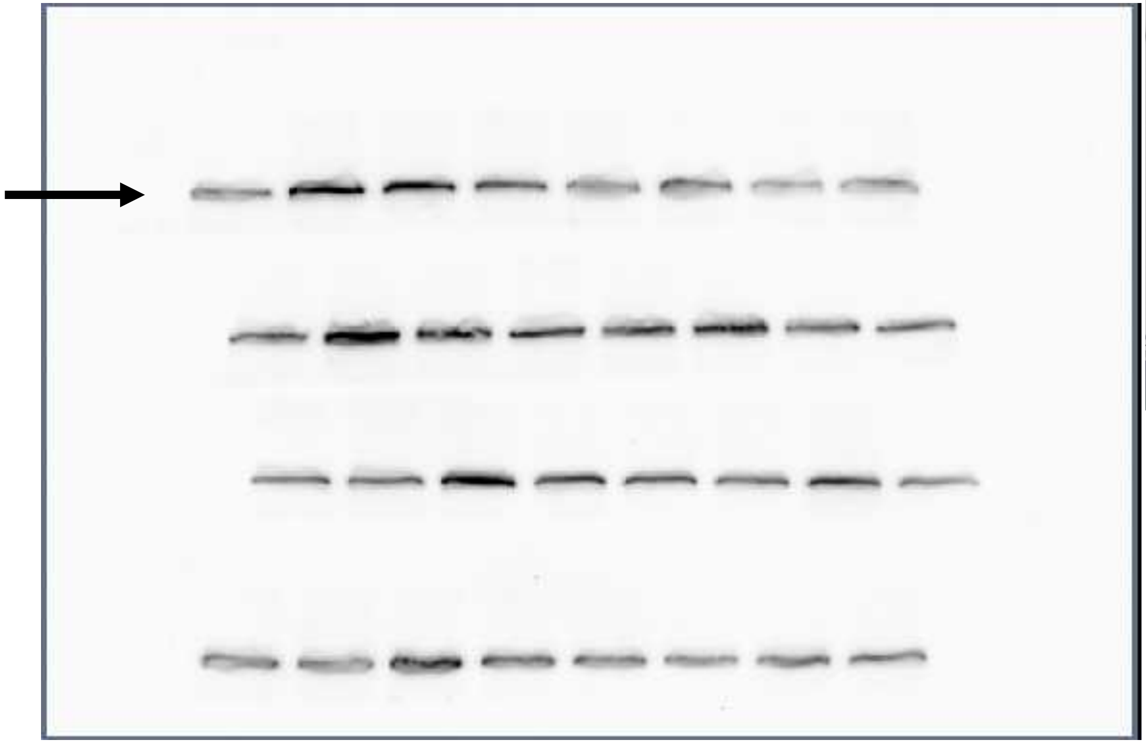

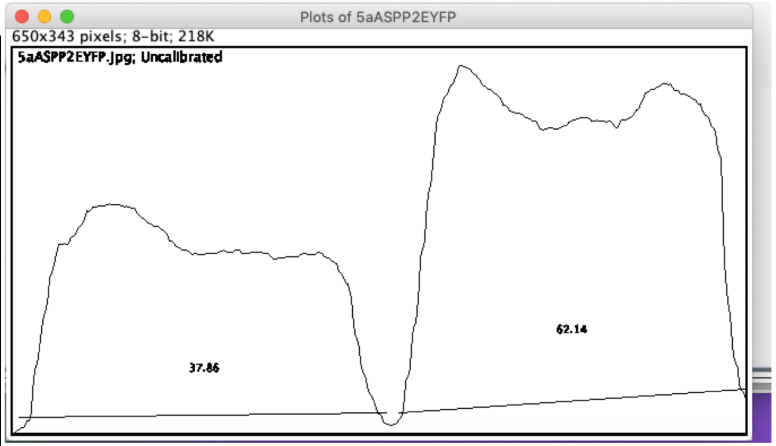

Figure 5a GAPDH/GST HEK293

52.0 48,0

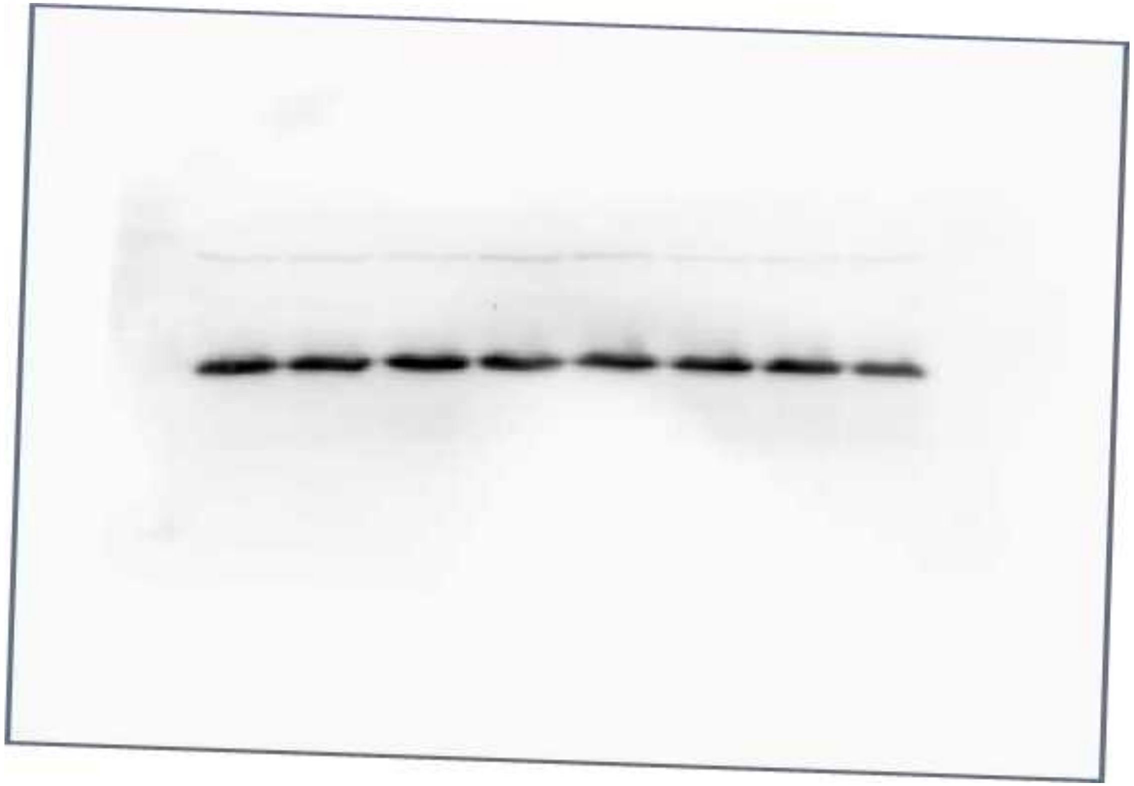

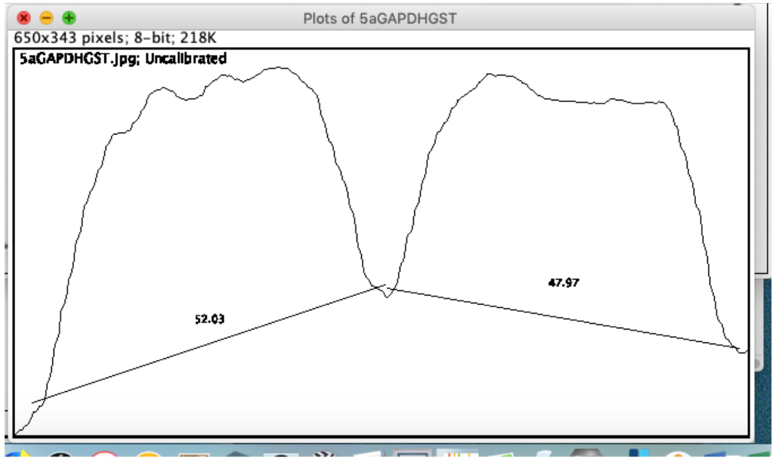

Figure 5a GAPDH/EYFP HEK293

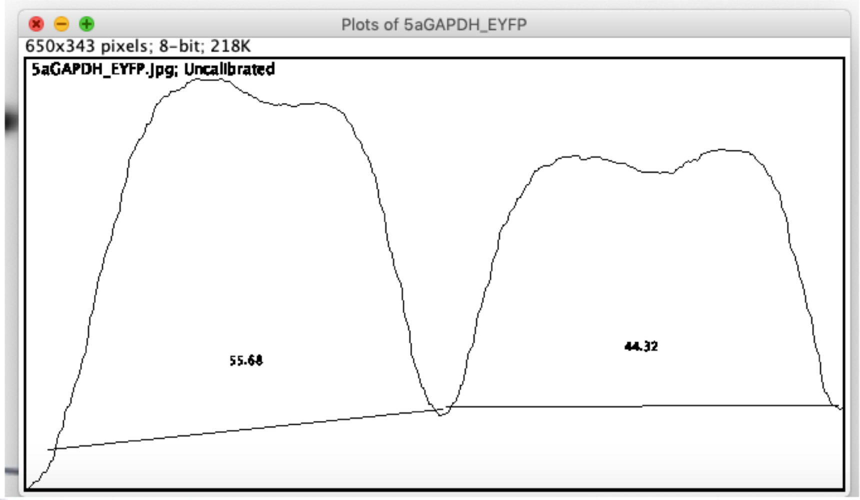

55.7 44,3

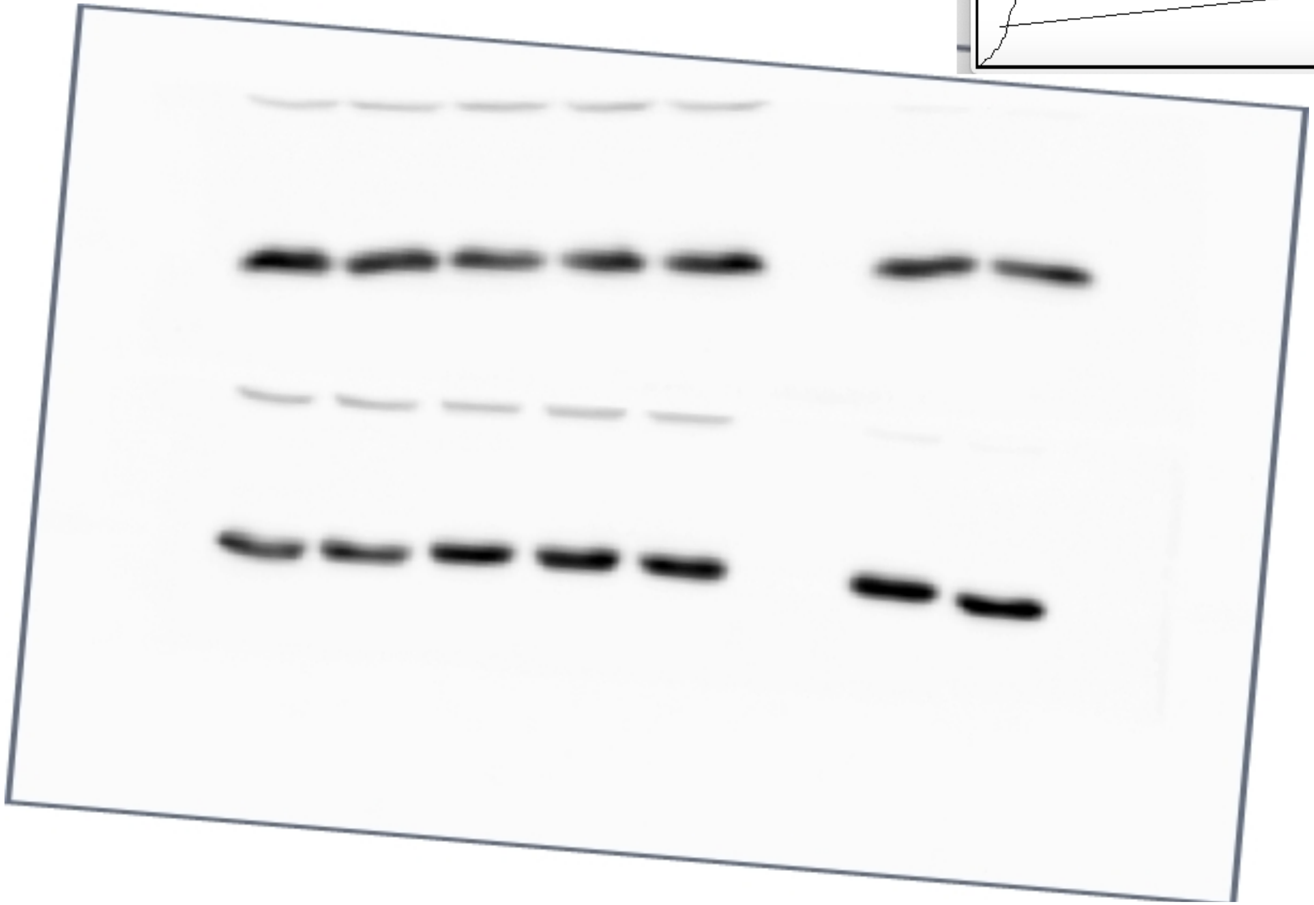

Figure 5a GFP

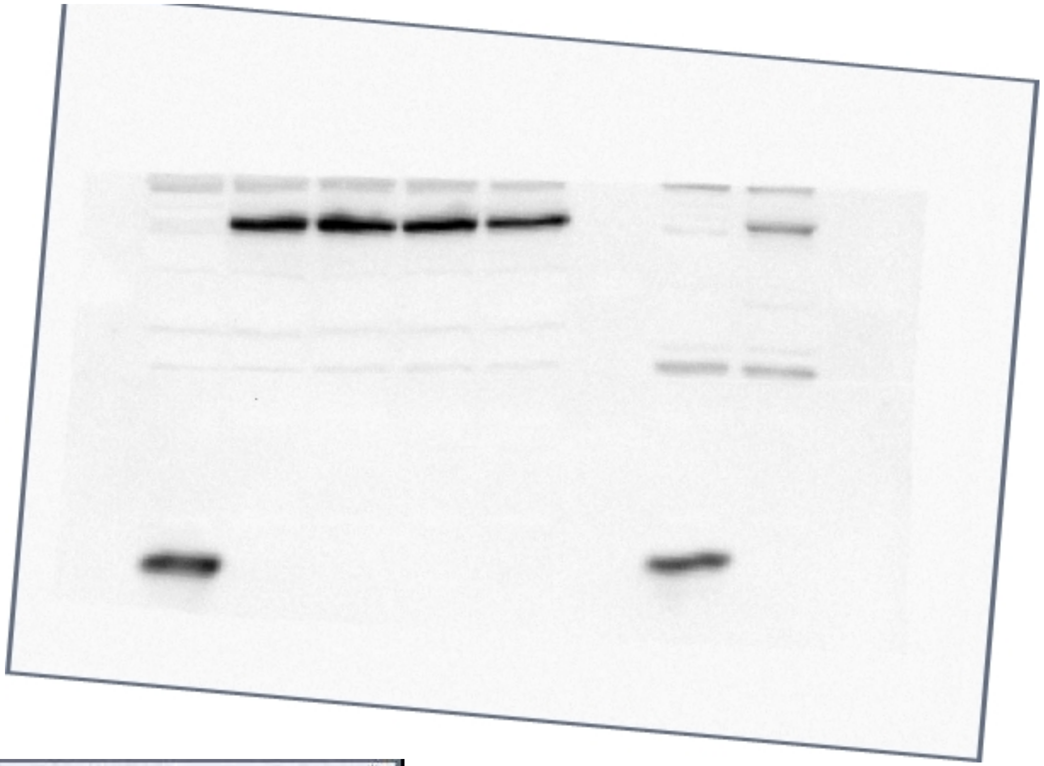

Figure 5a GST

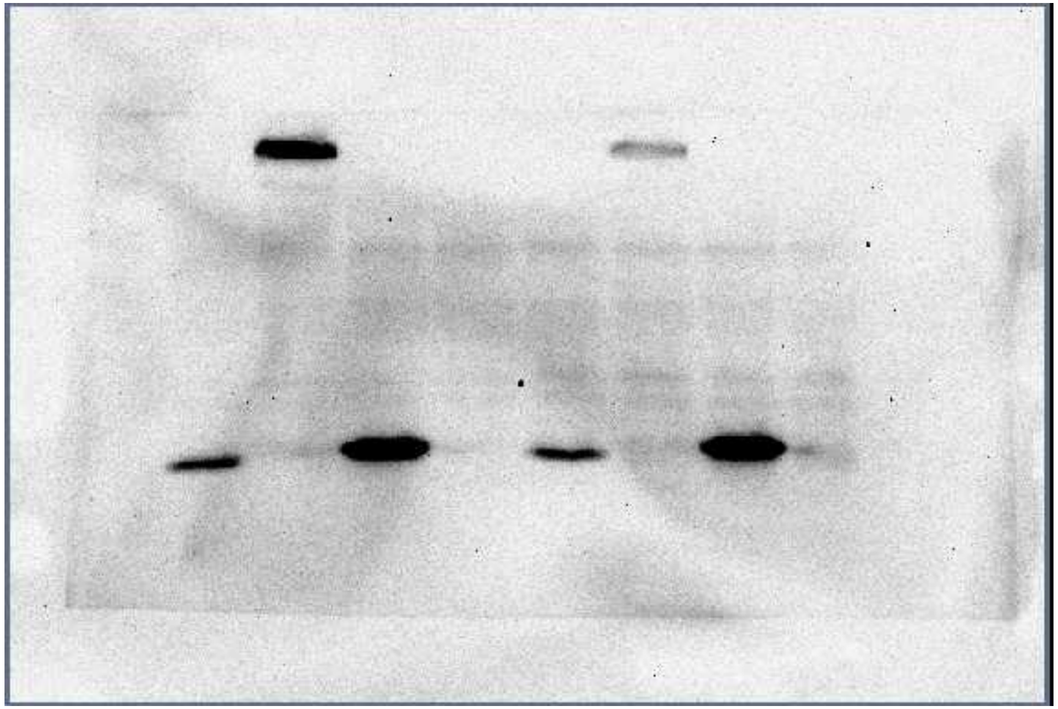

Figure 5a ASPP2 HeLa

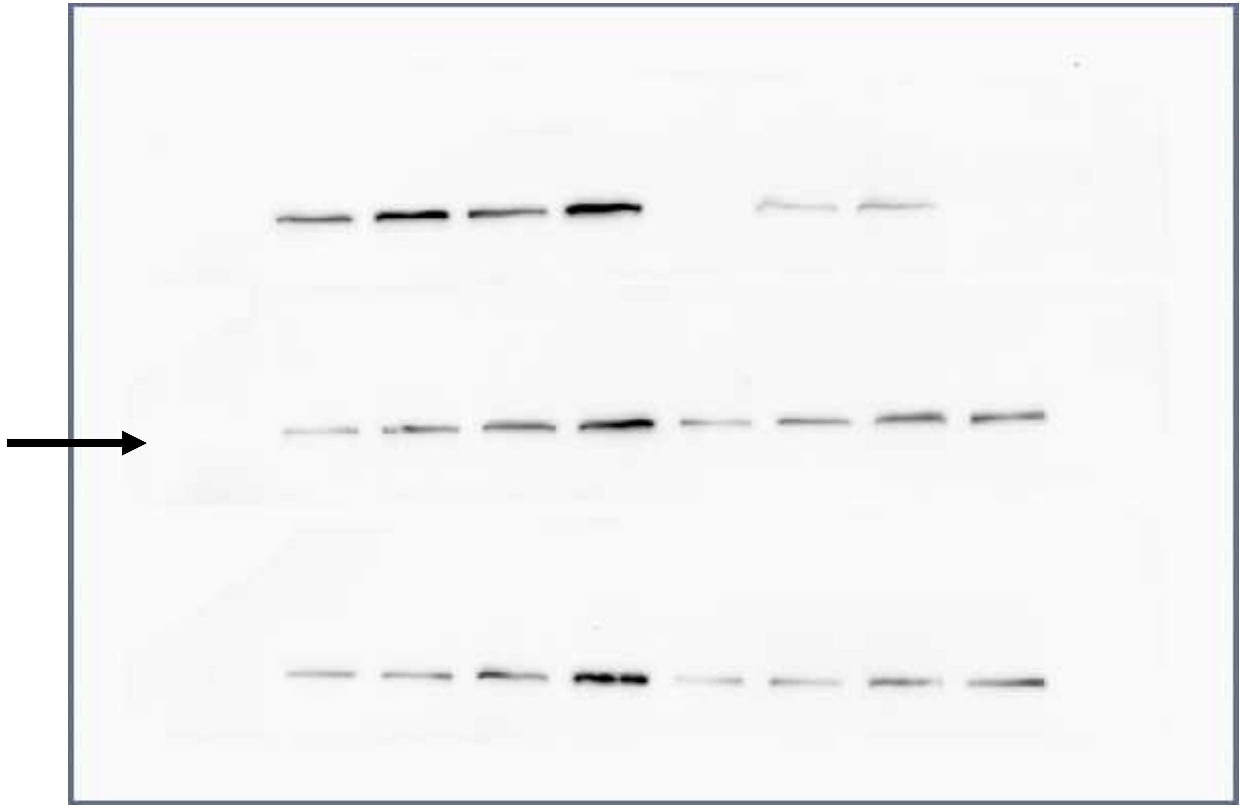

7,2 19.0 25.2 48.7

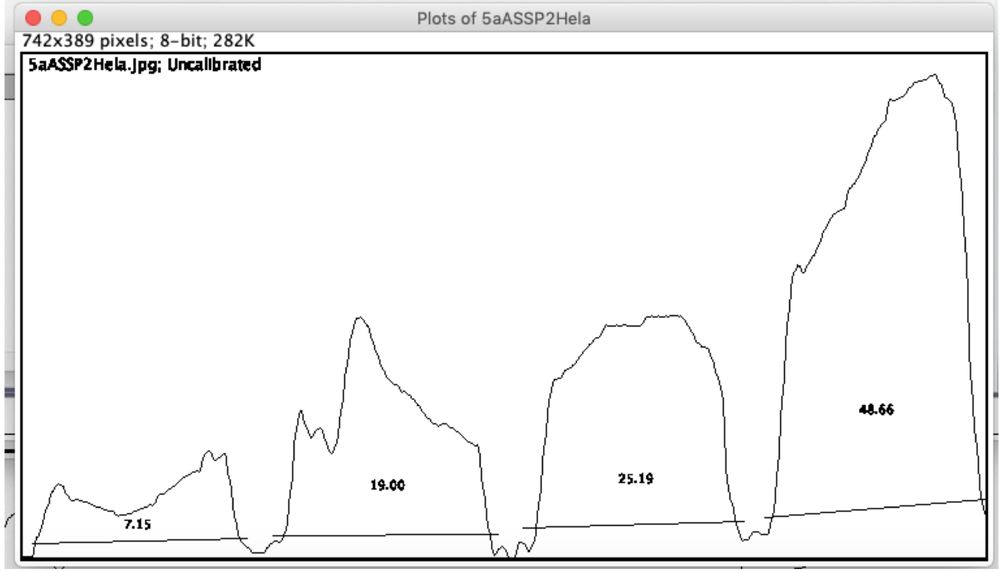

Figure 5a GAPDH HeLa

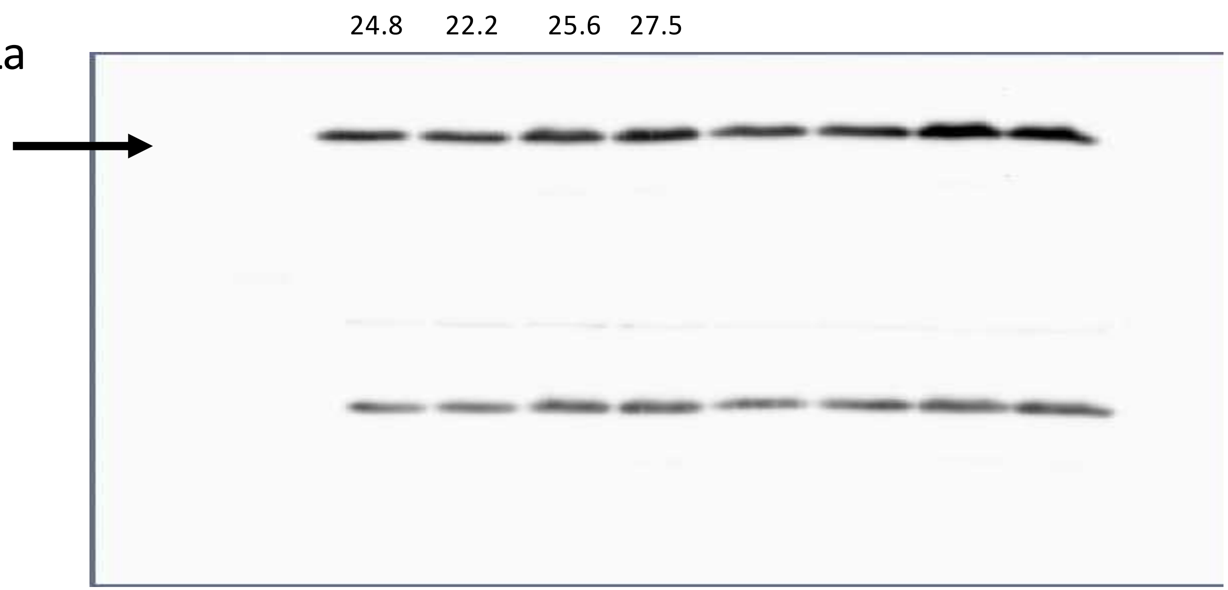

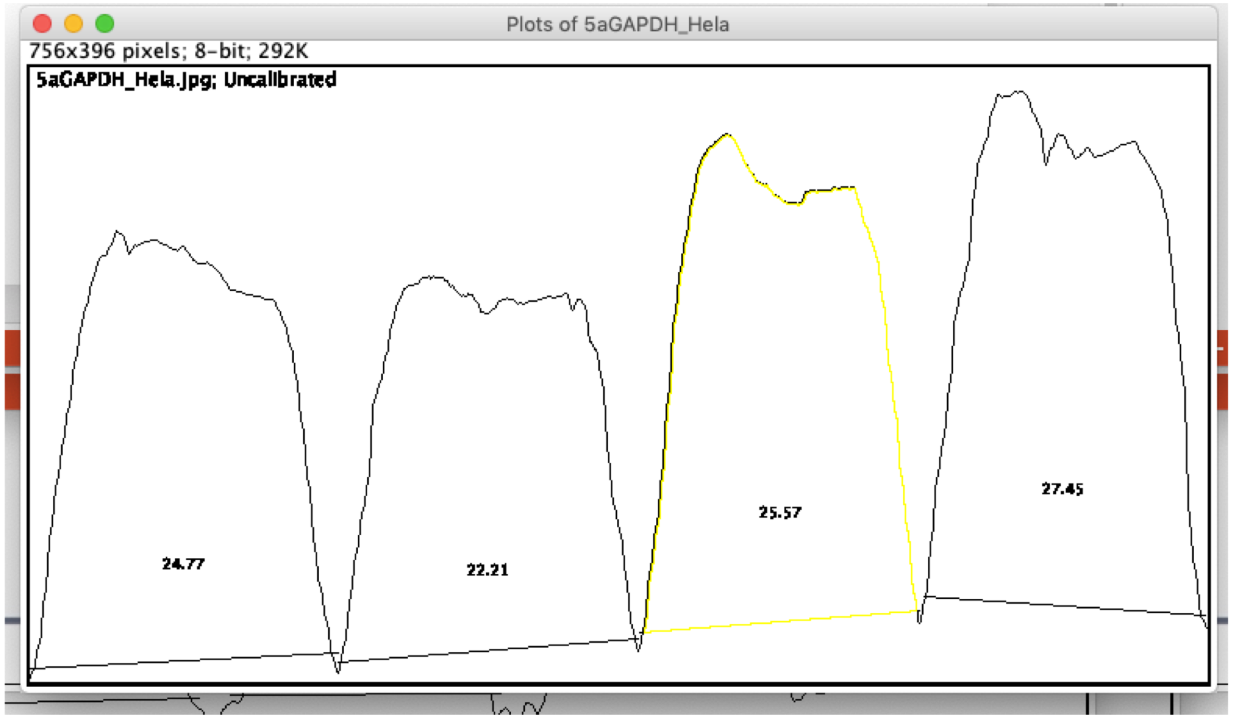

Figure 5a GFP/GST HeLa

49.1 50.9

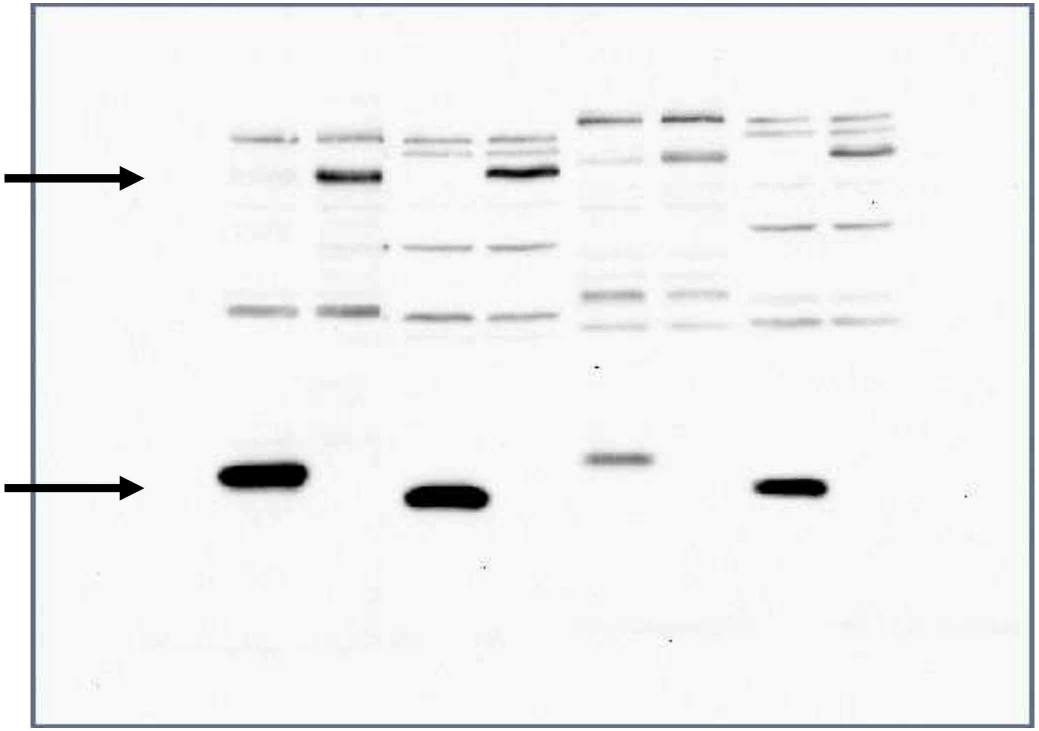

53.0 47.0

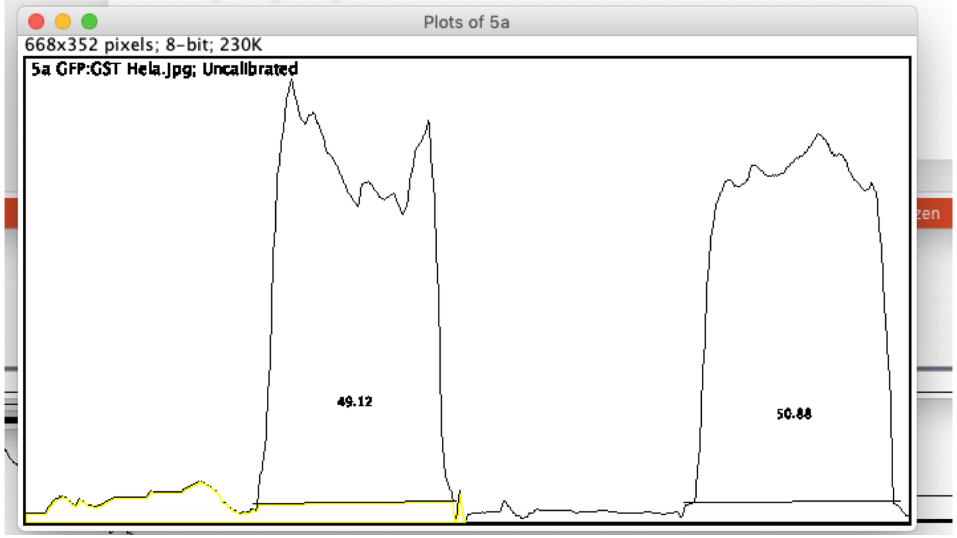

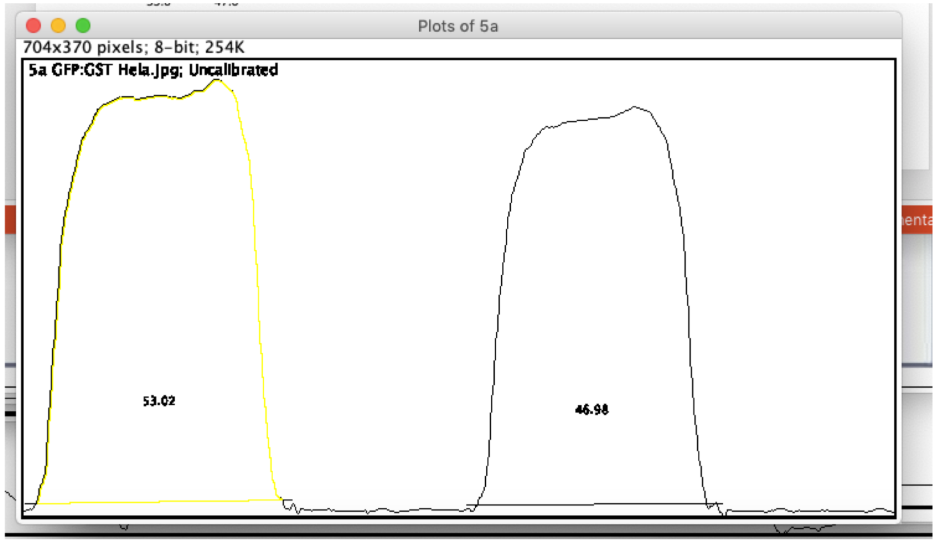

Figure 5b ASPP2

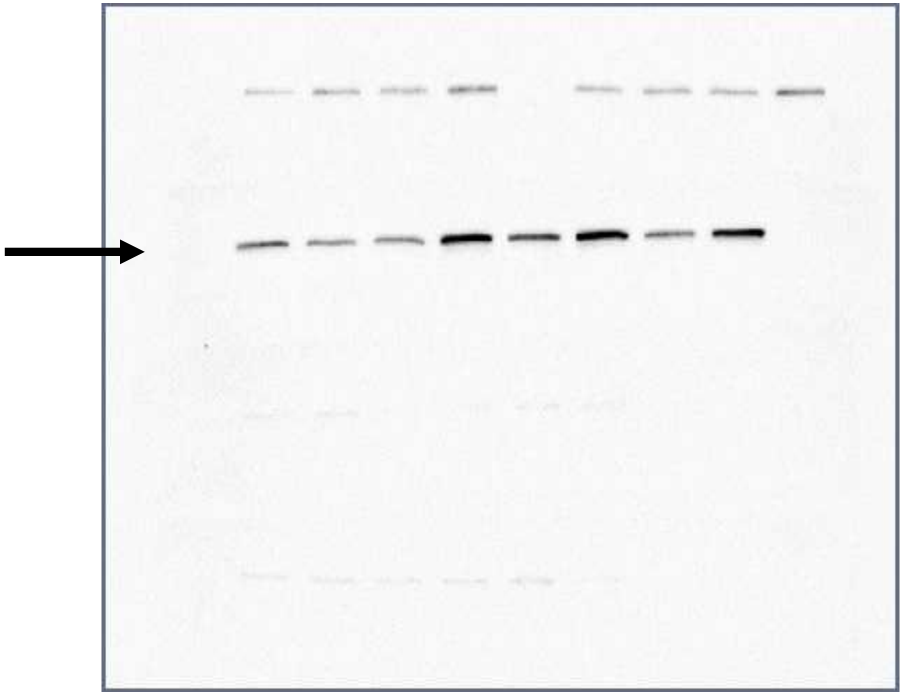

8,8 5.2 6.4 20.8 12.2 21.0 7.6 18.7

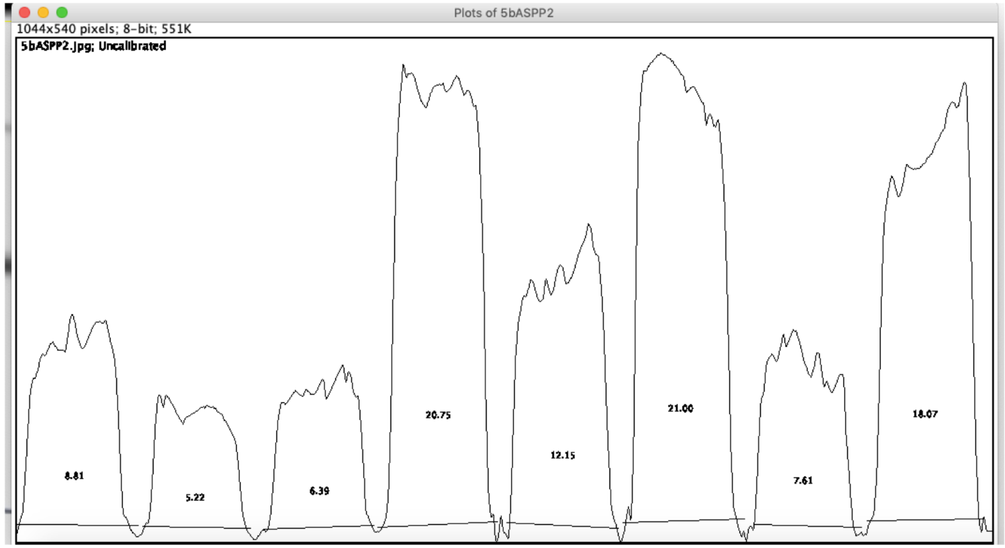

Figure 5b RASSF10

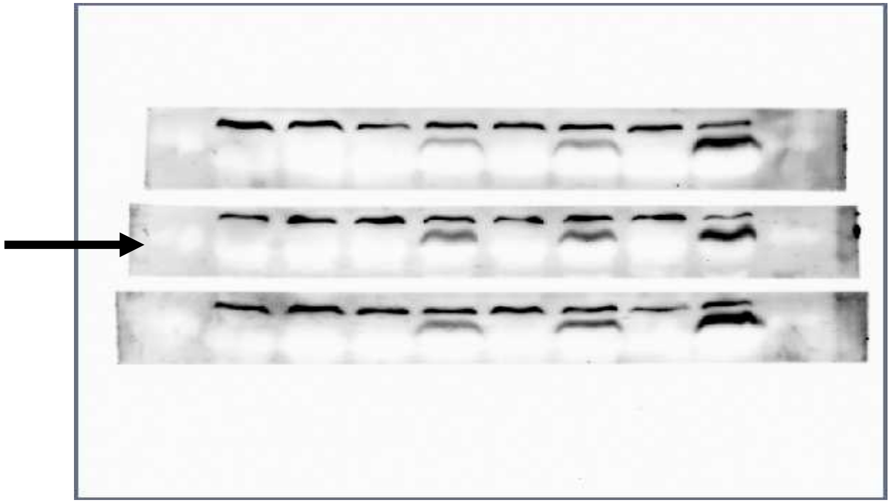

26.6 26.4 47.0

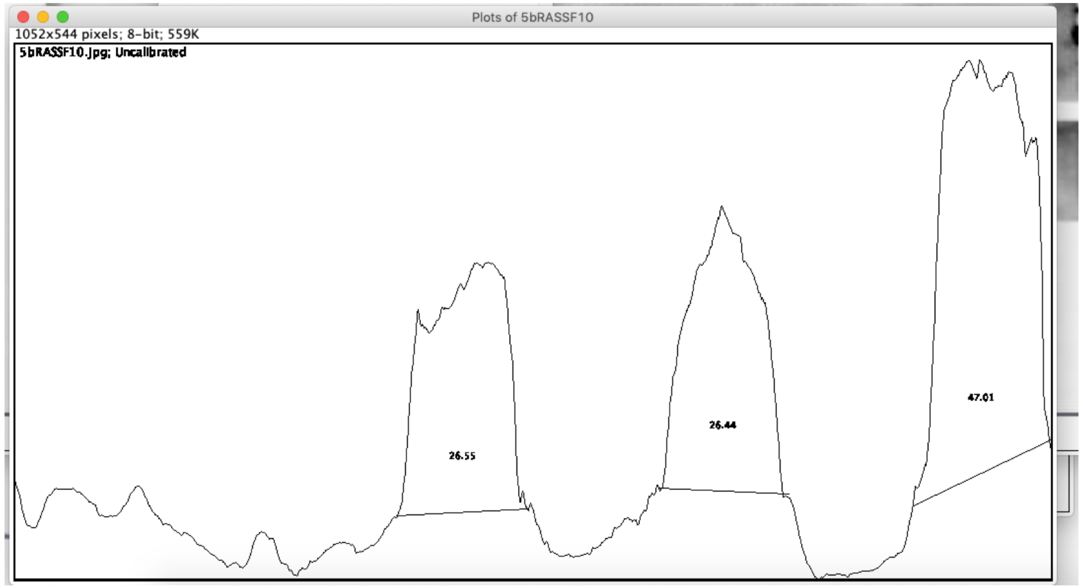

Figure 5b GAPDH

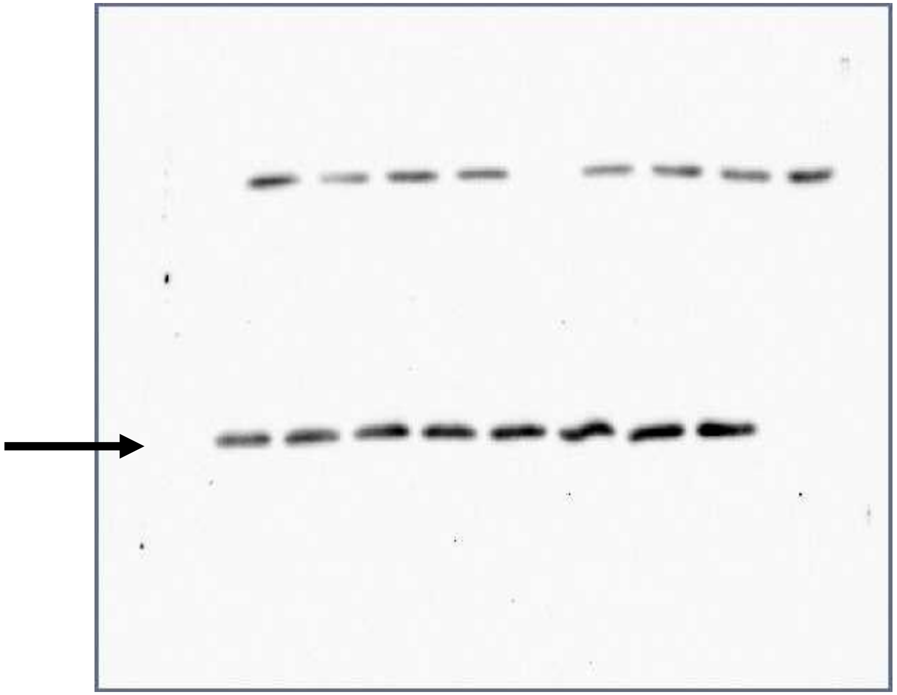

8,9 10.1 11.3 11.7 13.0 14.3 15.2 15.5

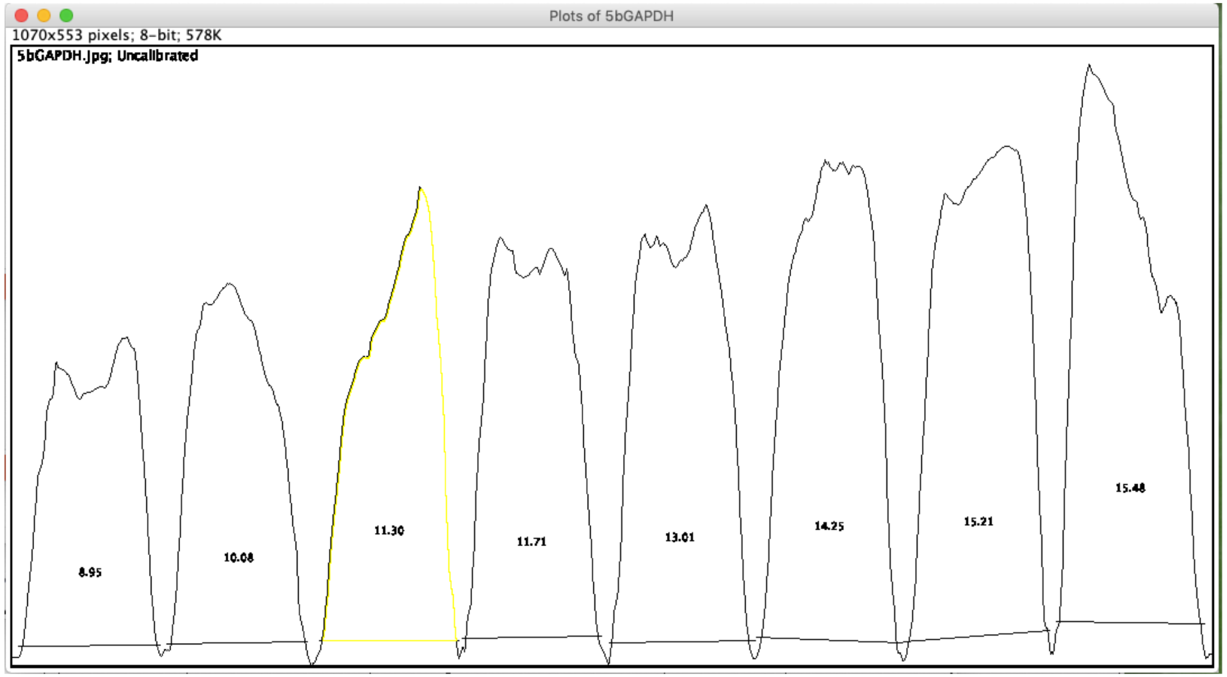

Figure 5d ASPP2

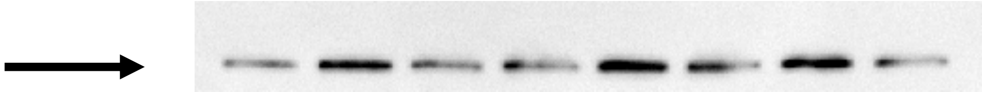

10.4 27.7 13.9 15.5 32.4

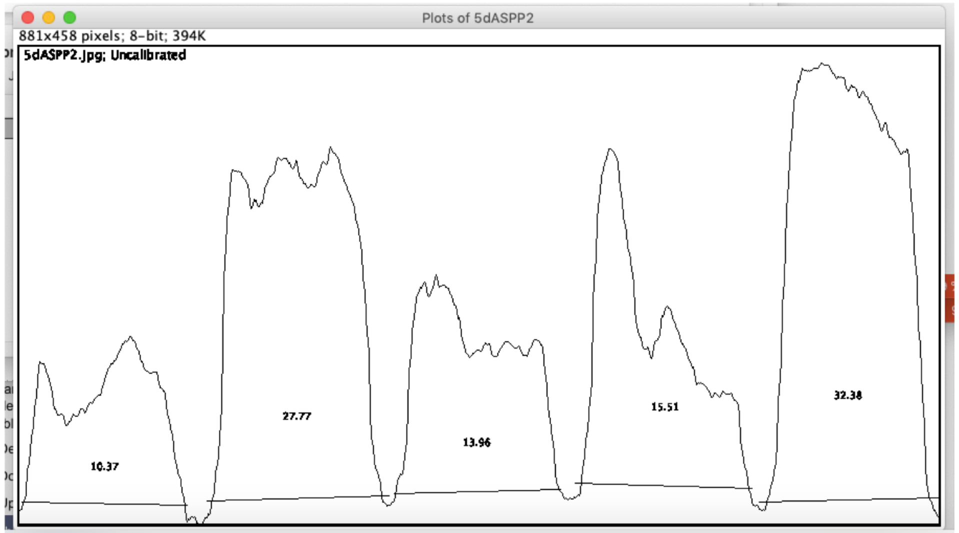

Figure 5d Flag

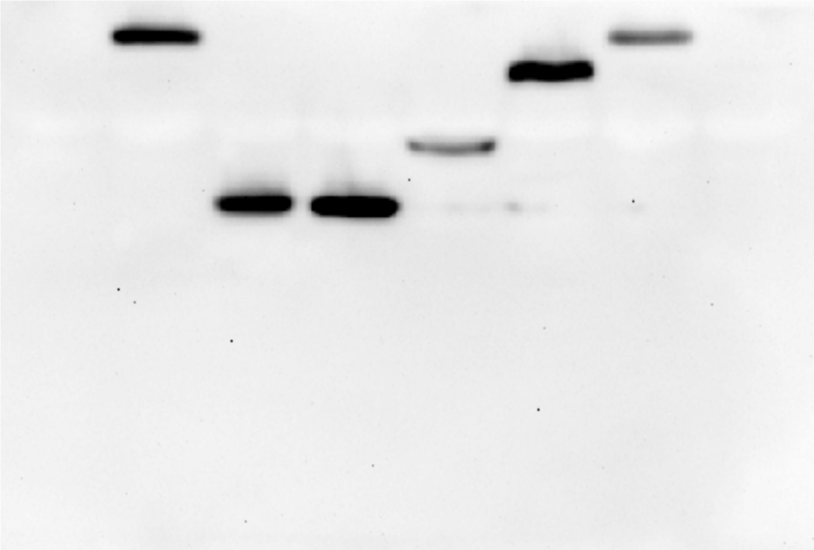

Figure 5d GAPDH

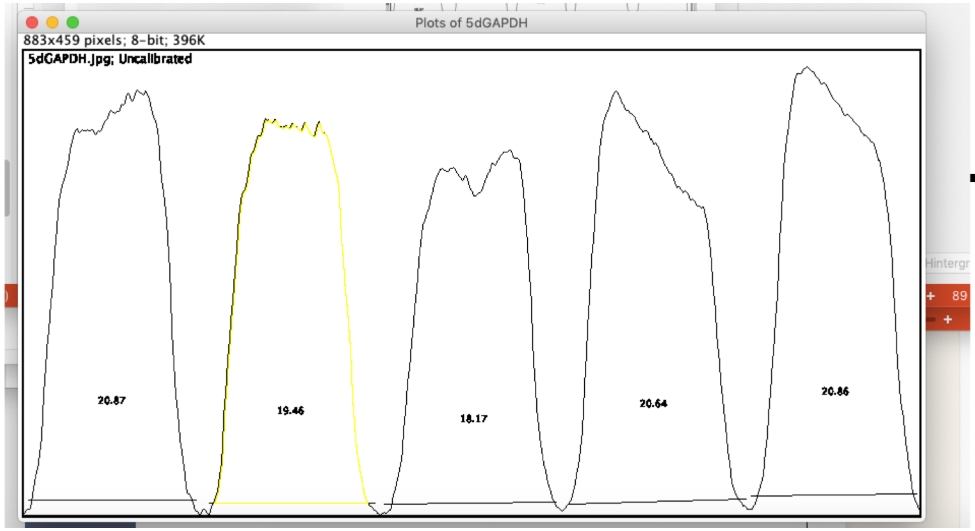

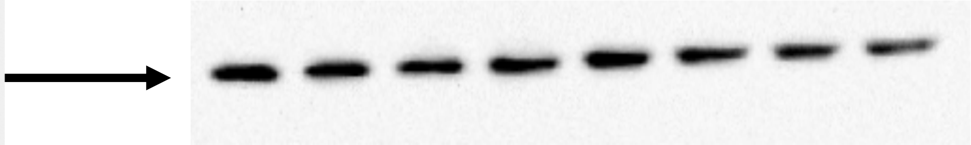

20.9 19.5 18.7 20.6 20.9

Figure 5e ASPP2 upper panel

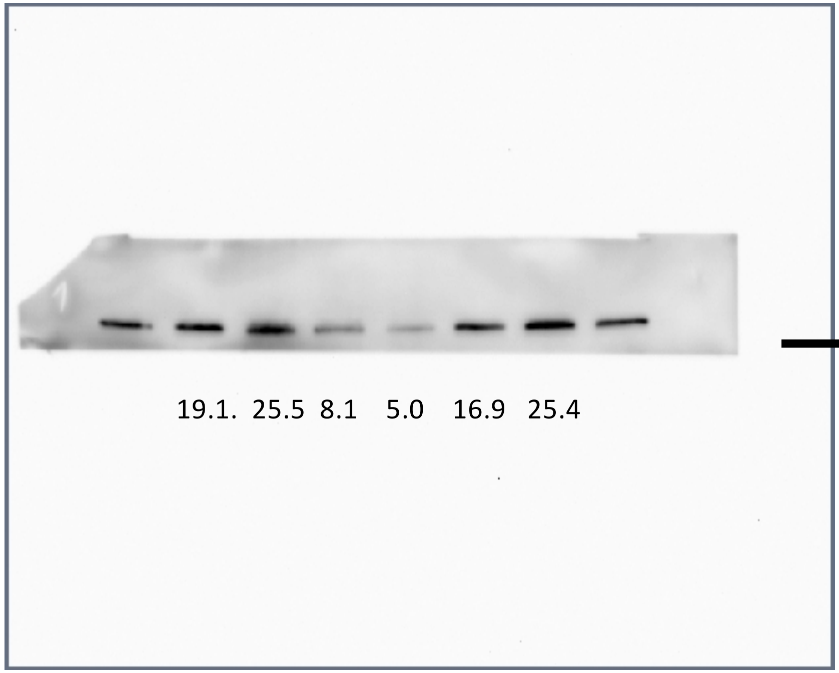

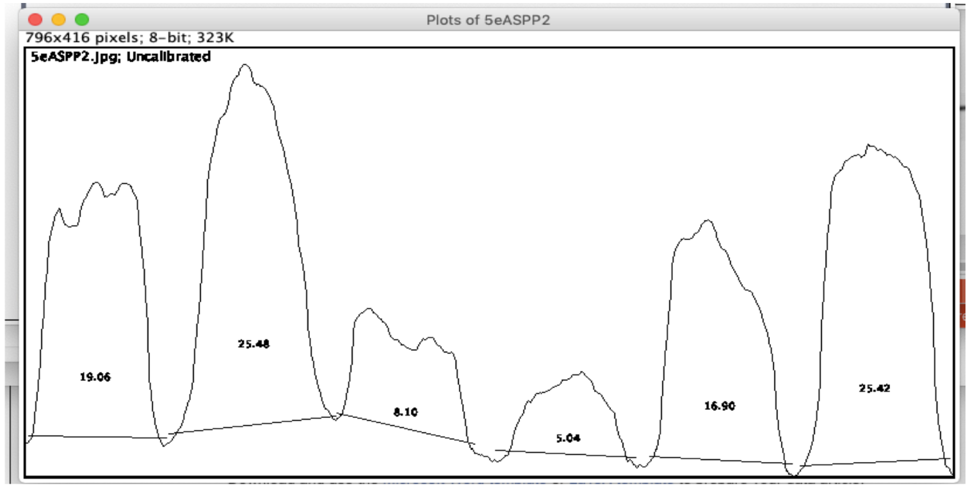

Figure 5e ASPP2 lower panel

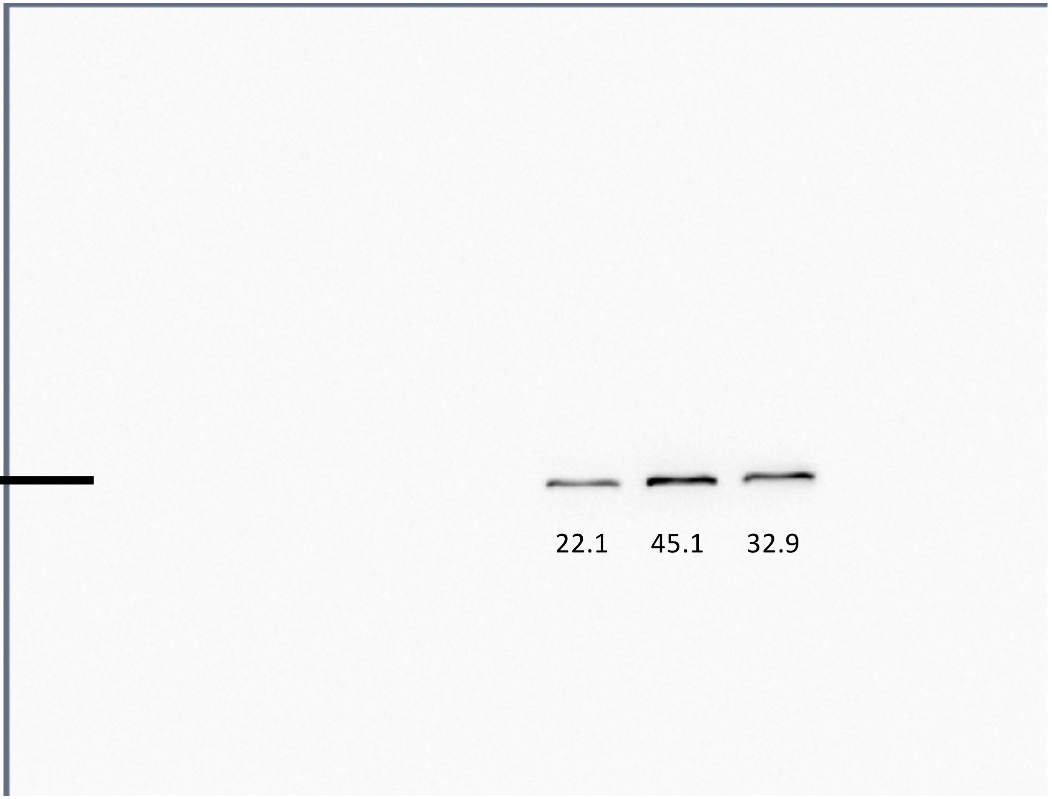

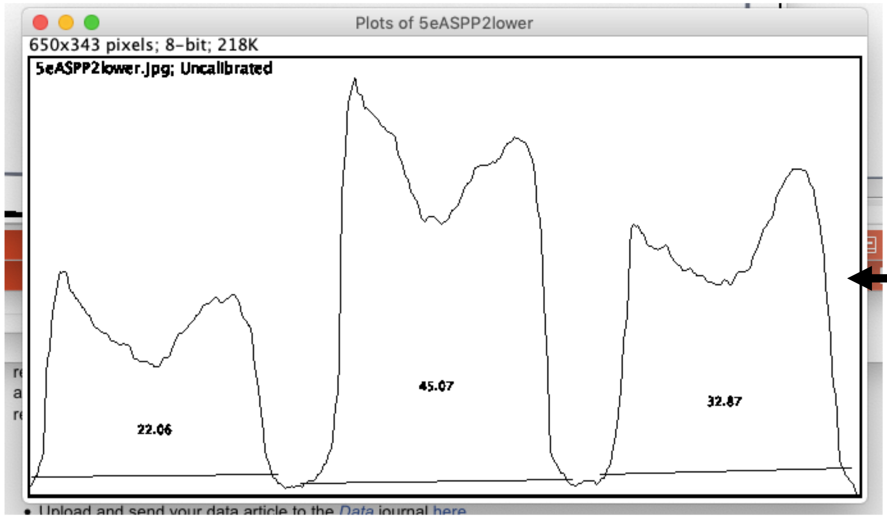

Figure 5e GAPDH upper panel

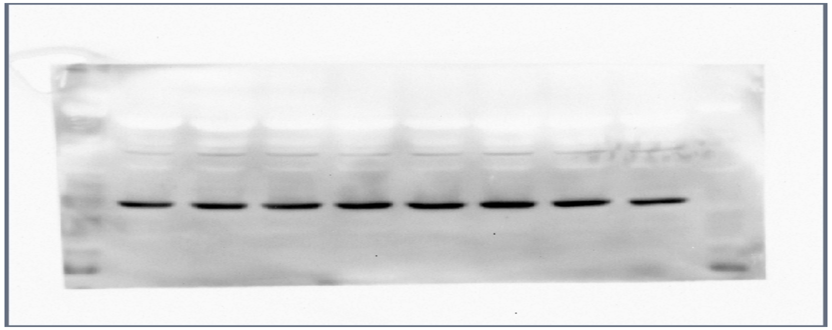

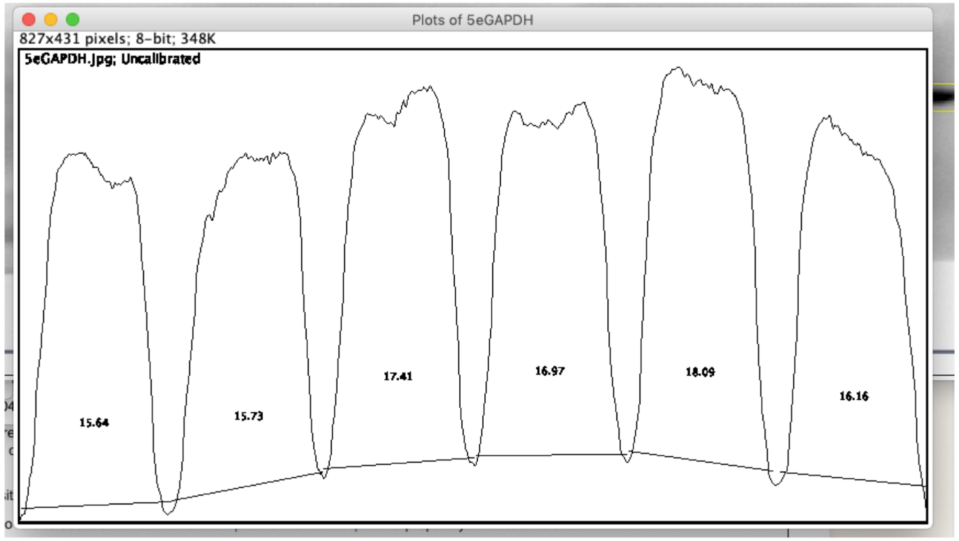

15.6. 15.7 17.4 16.9 18.1 16.2

Figure 5e GAPDH lower panel

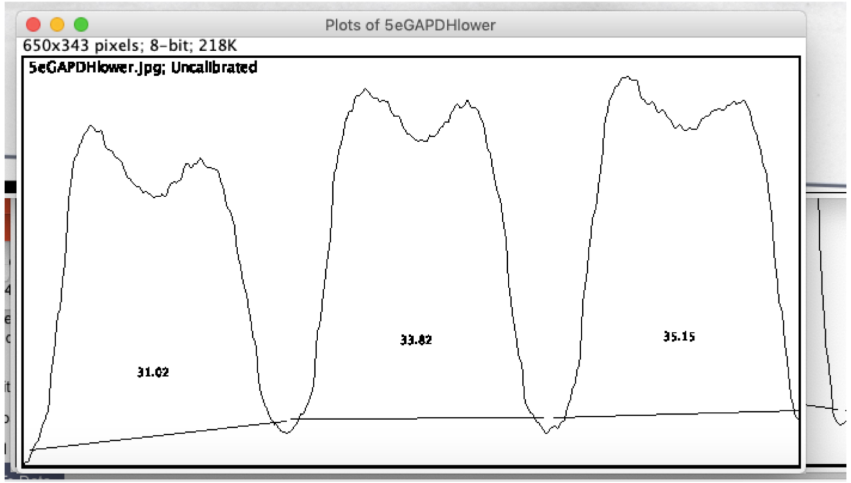

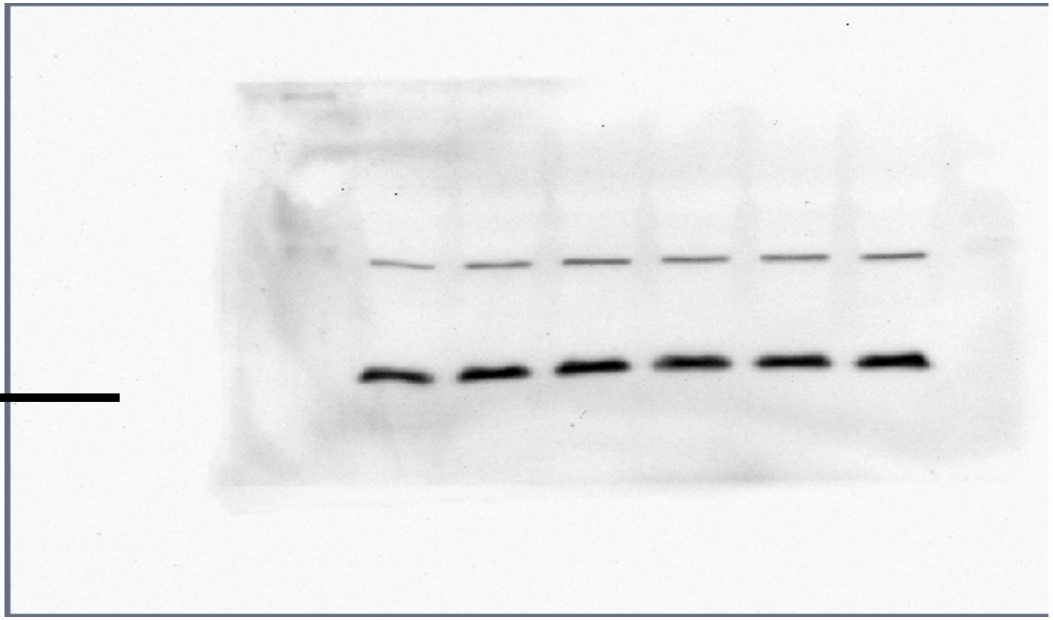

31.0 33.8 35.2

Figure 5e FLAG upper panel

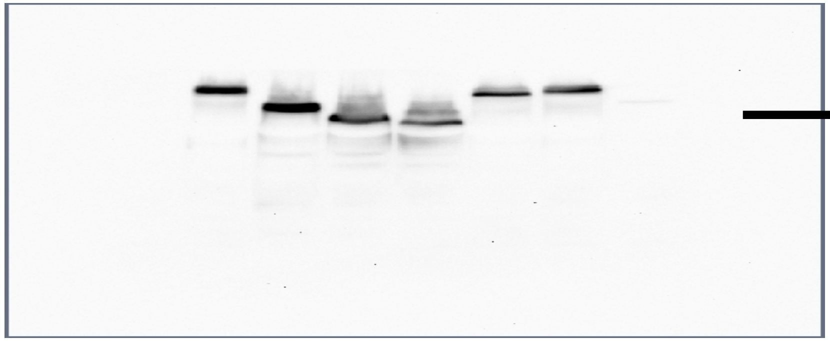

16.1 23.5 21.8 15.9 11.0 11.7

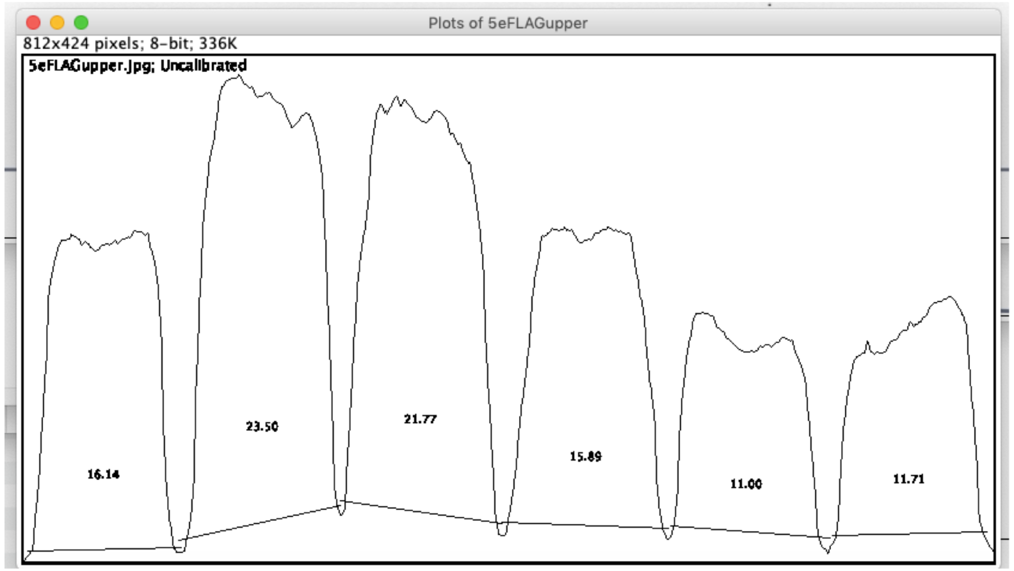

Figure 5e FLAG lower panel

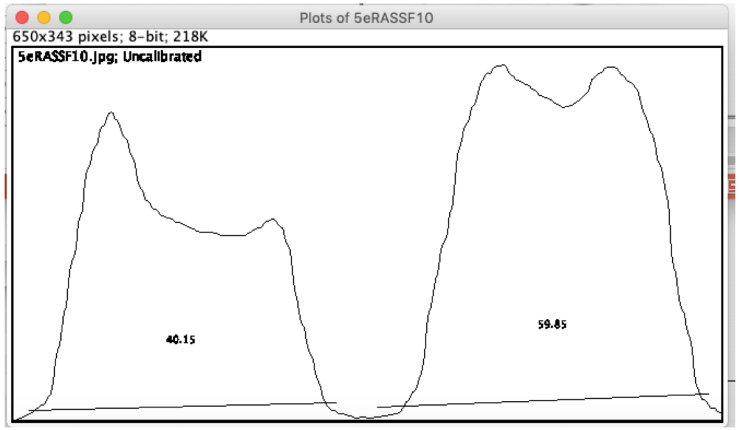

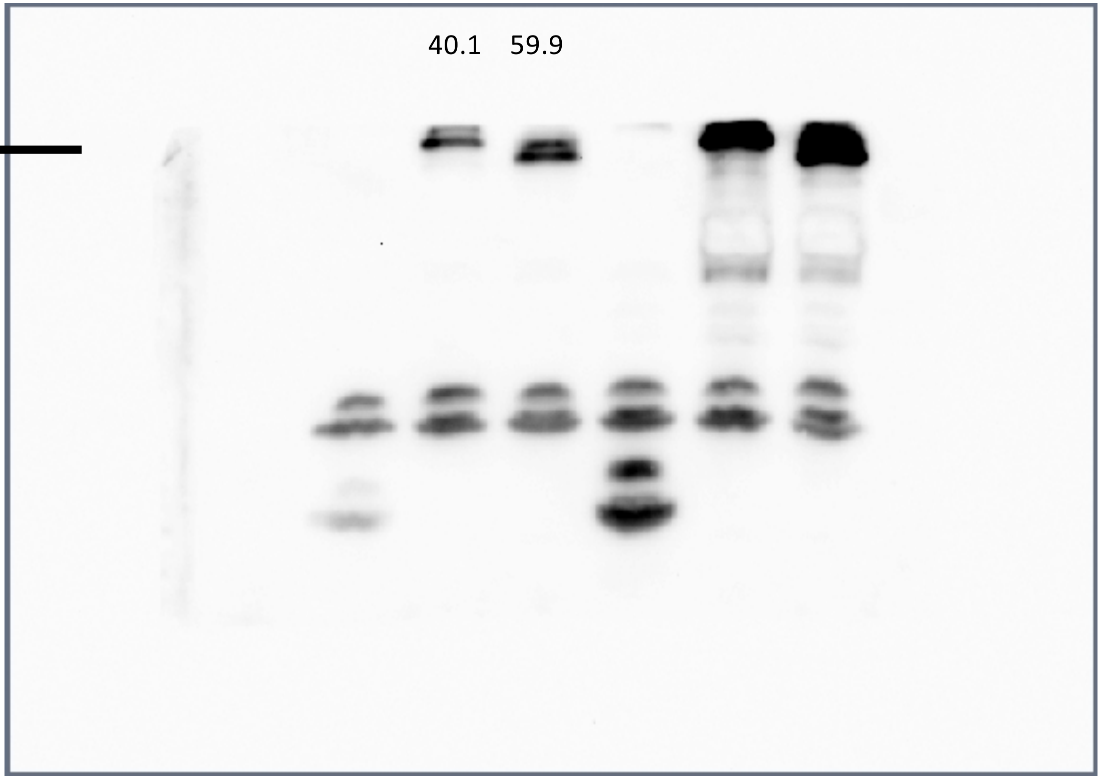

Figure 9 YAP1

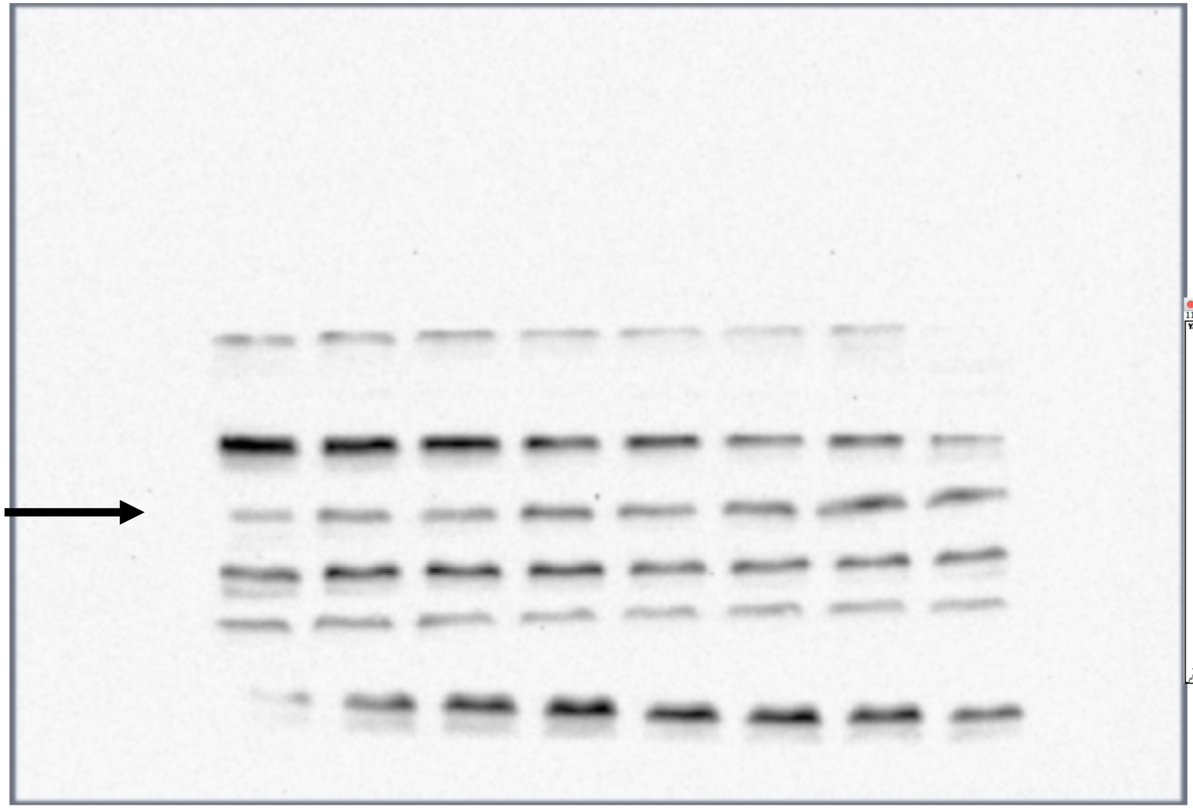

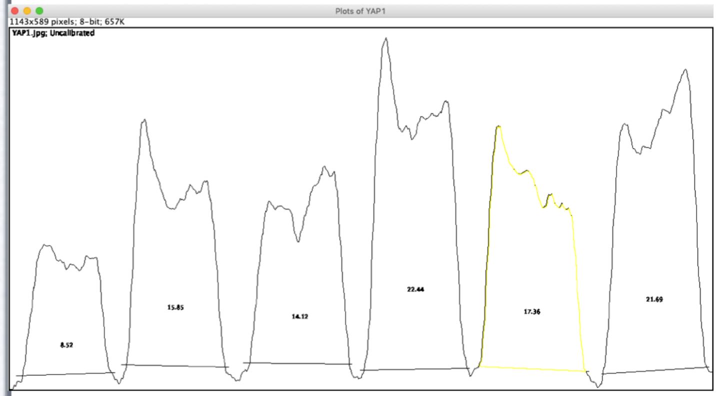

8.52 15.82 14.12 22.44 17.36 21.69

Figure 9 ßCatenin

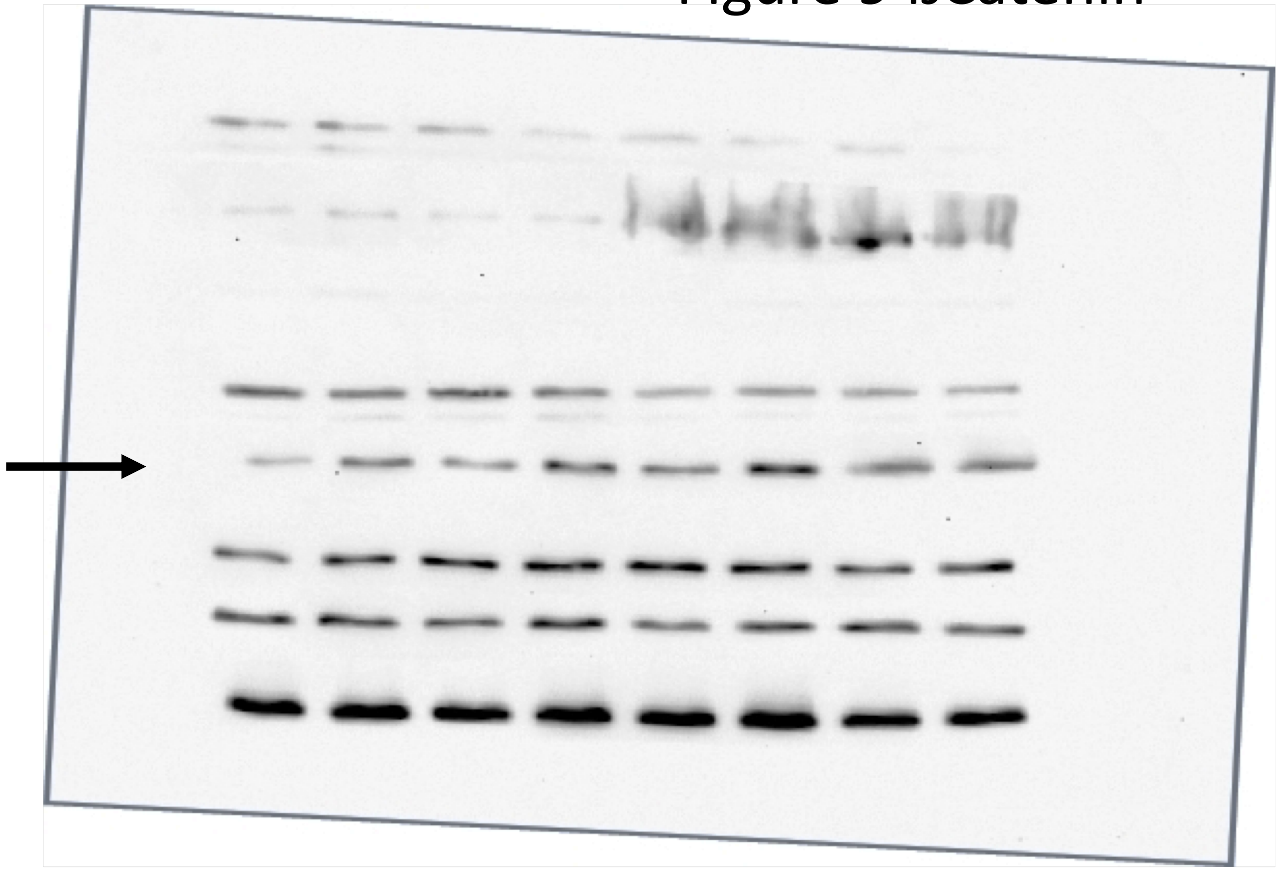

6.1 18.1 8.8 22.8 16.0 27.5

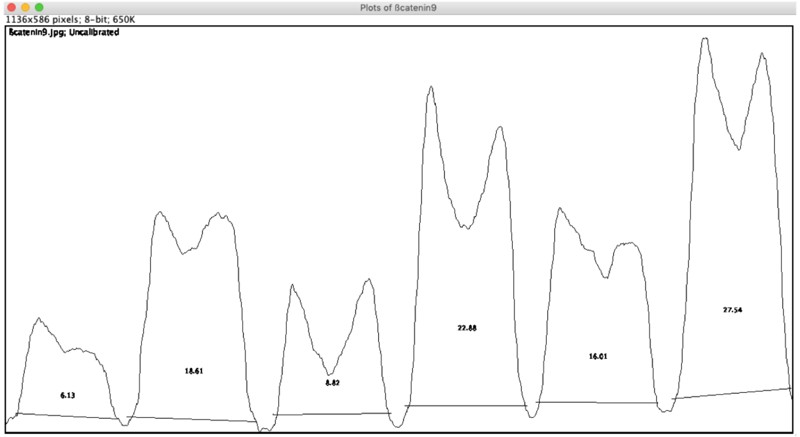

Figure 9 Phospho SMAD2

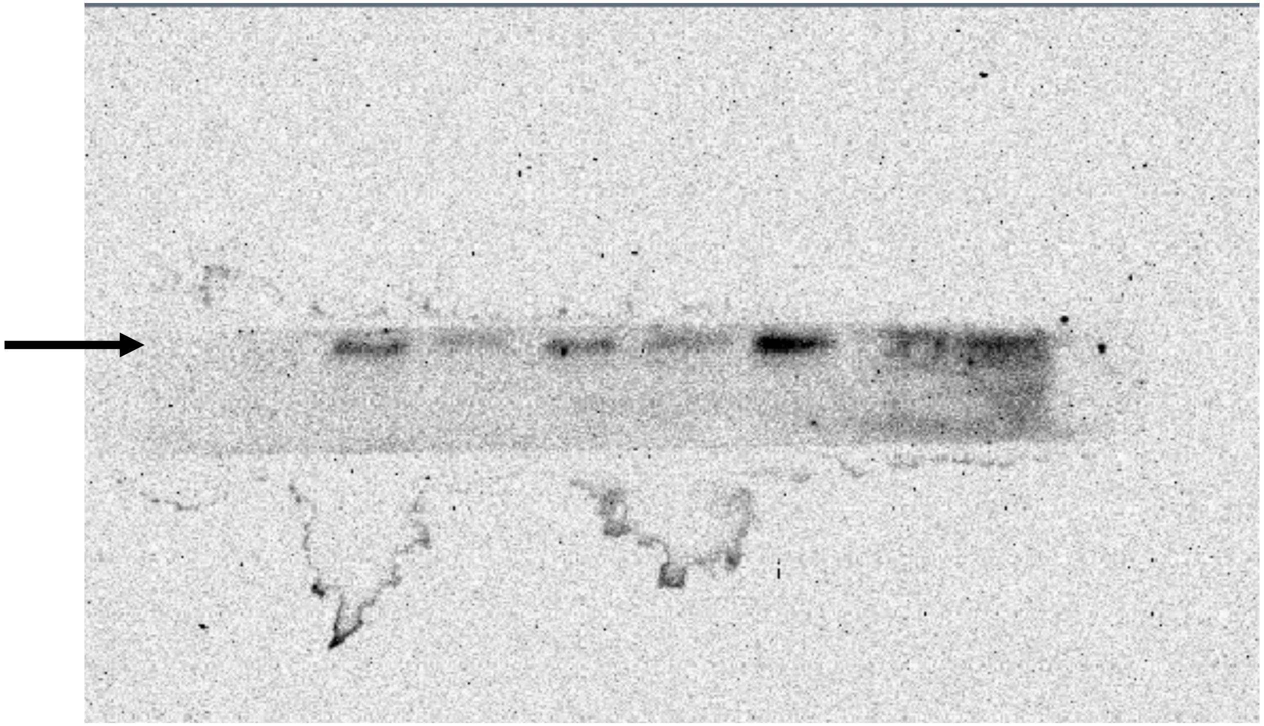

4.1 21.3 10.4 17.5 12.8 33.8

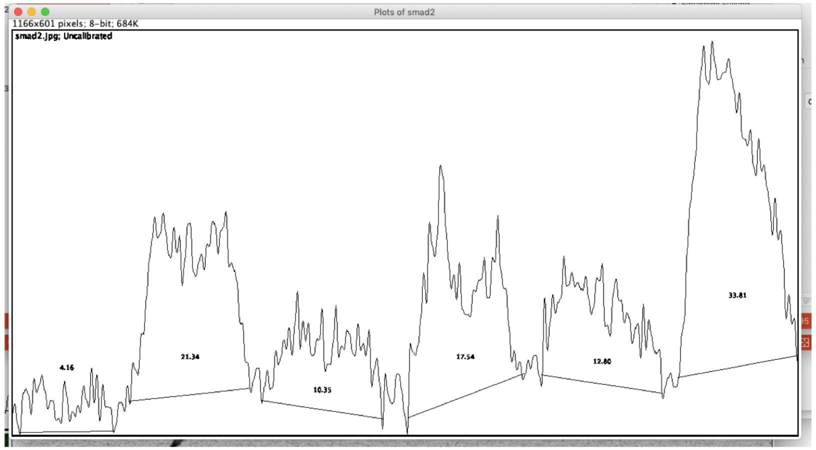

Figure 9 ASPP2

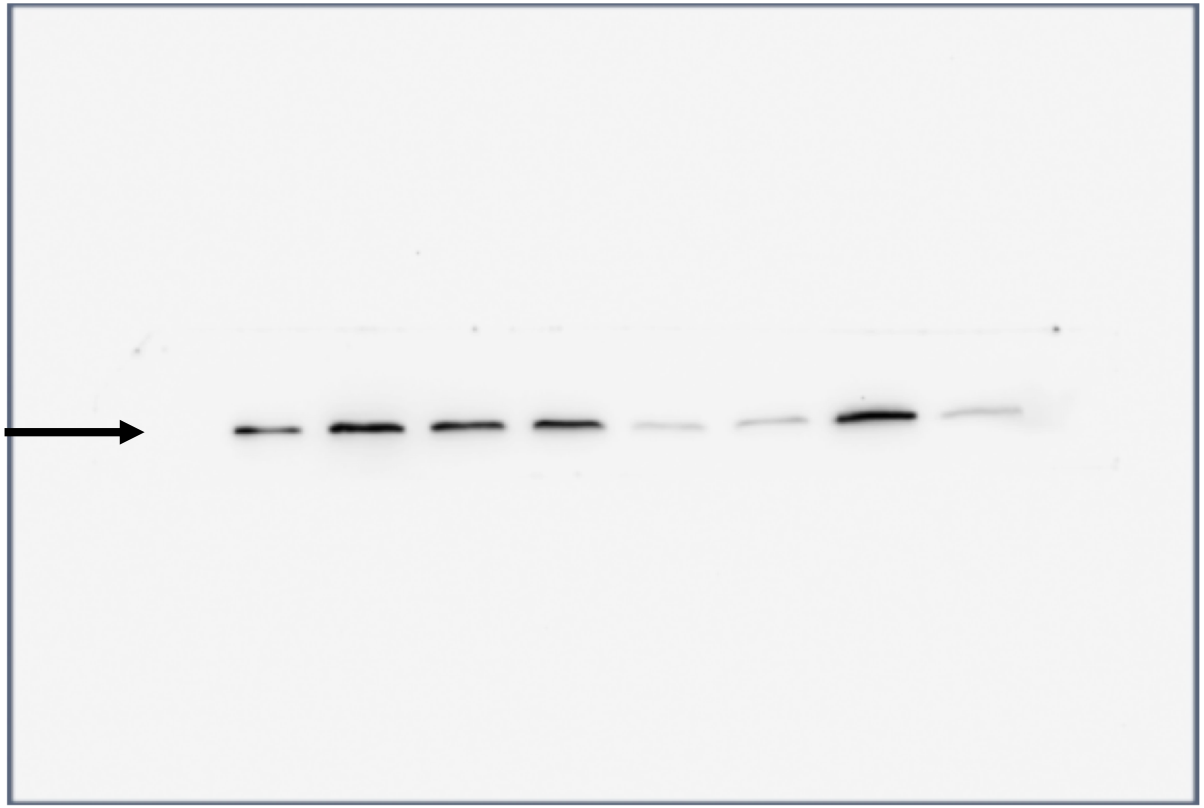

12.3 24.5 18.9 19.4 2.6 3.5

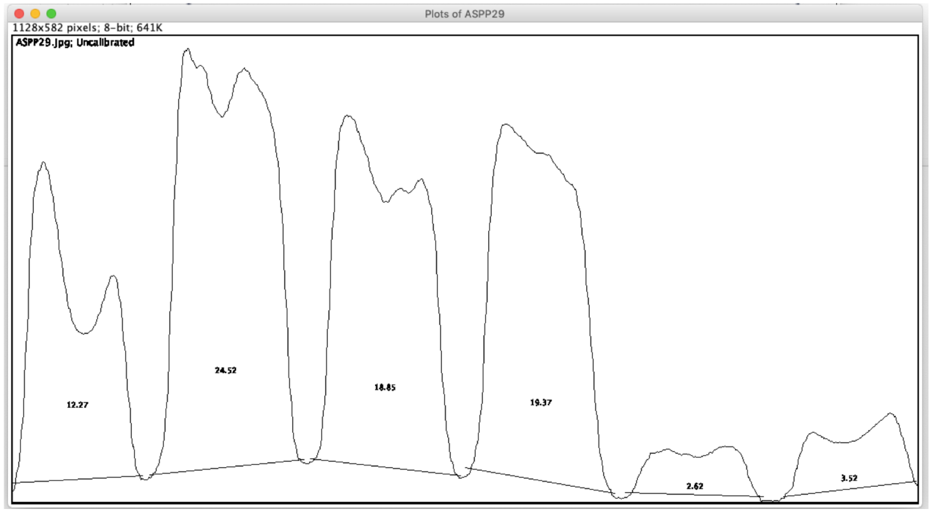

Figure 9 GAPDH

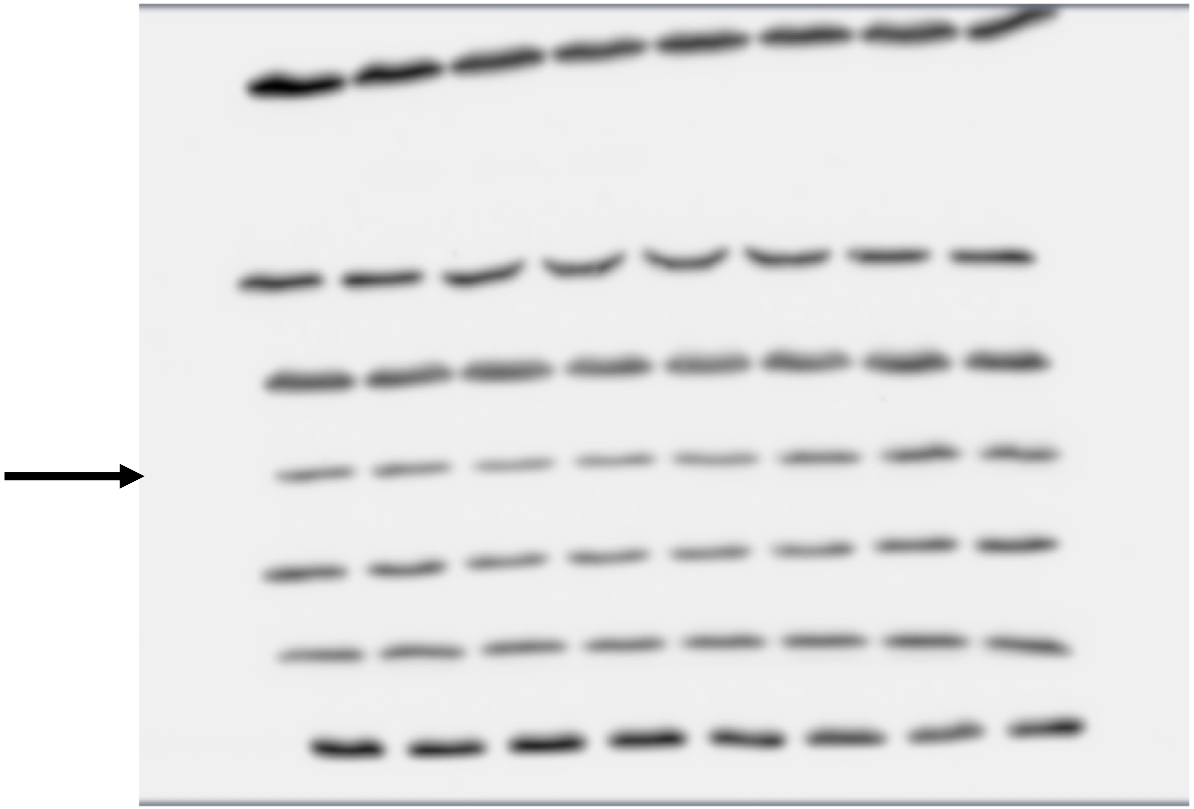

18.0 17.0 14.4 14.3 16.3 19.9

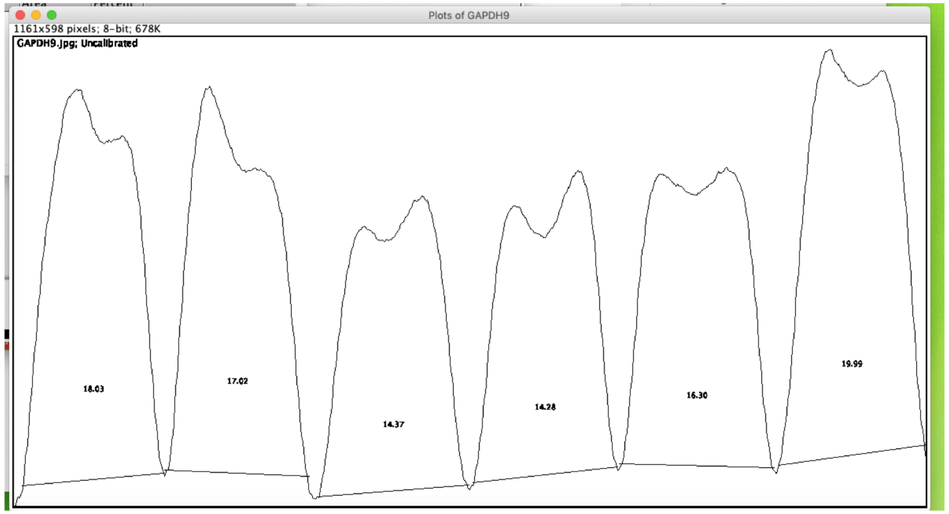

Supplement: Supplementary file 1 [file cancers-11-01976-s001.pdf]
